# Five New Tamarixetin Glycosides from *Astragalus thracicus* Griseb. Including Some Substituted with the Rare 3-Hydroxy-3-methylglutaric Acid and Their Collagenase Inhibitory Effects In Vitro

**DOI:** 10.1021/acsomega.3c09677

**Published:** 2024-04-08

**Authors:** Hristo Vasilev, Karel Šmejkal, Sabina Jusková, Jiri Vaclavik, Jakub Treml

**Affiliations:** † Department of Pharmacognosy, Faculty of Pharmacy, Medical University, 2 Dunav Street, Sofia 1000, Bulgaria; ‡ Department of Natural Drugs, Faculty of Pharmacy, Masaryk University, Palackého tř. 1946/1, Brno 61200, Czech Republic; § Department of Molecular Pharmacy, Faculty of Pharmacy, Masaryk University, Palackého tř. 1946/1, Brno 61200, Czech Republic

## Abstract

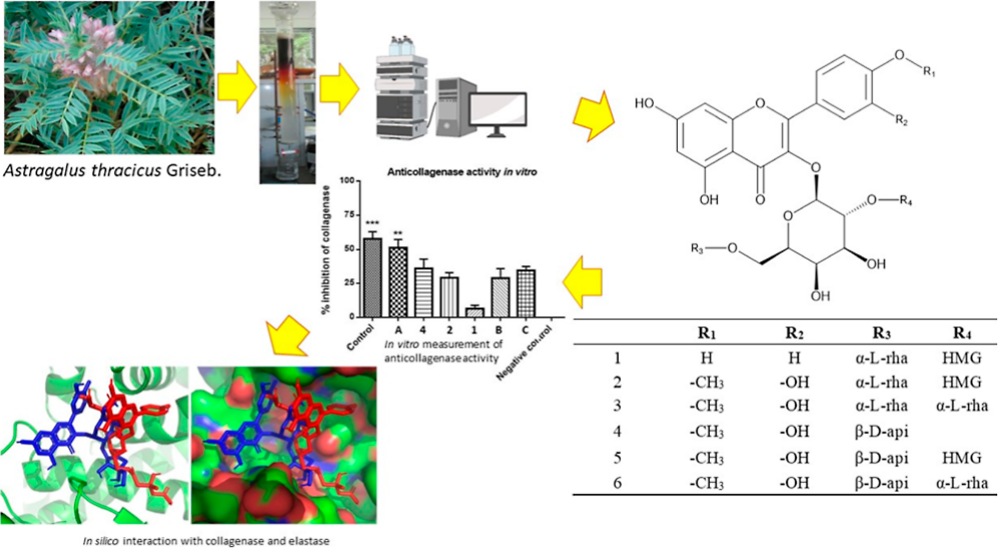

Along with the known kaempferol-3-*O*-α-l-rhamnopyranosyl-(1 → 2)-[6-*O*-(3-hydroxy-3-methylglutaryl)]-β-d-galactopyranoside (**1**), five new flavonoids, containing the rarely isolated aglycon tamarixetin, were isolated from a methanolic extract of the endemic Balkan species *Astragalus thracicus* Griseb. Three of the new compounds are substituted with 3-hydroxy-3-methylglutaryl residue (HMG), untypical for the genus *Astragalus*. The compounds were identified as tamarixetin-3-*O*-α-l-rhamnopyranosyl-(1 → 2)-[6-*O*-(3-hydroxy-3-methylglutaryl)]-β-d-galactopyranoside (**2**), tamarixetin-3-*O*-(2,6-di-*O*-α-l-rhamnopyranosyl)-β-d-galactopyranoside (**3**), tamarixetin 3-*O*-β-d-apiofuranosyl-(1 → 2)-β-d-galactopyranoside (**4**), tamarixetin-3-*O*-β-d-apiofuranosyl-(1 → 2)-[6-*O*-(3-hydroxy-3-methylglutaryl)]-β-d-galactopyranoside (**5**), and tamarixetin-3-*O*-β-d-apiofuranosyl-(1 → 2)-[α-l-rhamnopyranosyl-(1 → 6)]-β-d-galactopyranoside (**6**). Selected compounds from *A. thracicus* were tested to evaluate their anticollagenase activity. The greatest effect was observed for quercetin-3-*O*-β-d-apiofuranosyl-(1 → 2)-β-d-galactopyranoside, possibly due to the presence of an *ortho*-dihydroxy arrangement of flavonoid ring B. The effect on collagenase and elastase was further evaluated also by in silico study, and the test compounds showed some level of in silico interaction.

## Introduction

*Astragalus* genus (Fabaceae) belongs to the largest genera of vascular plants (with around 2,500 species),^1^ distributed mainly in the Northern hemisphere. In Bulgaria, *Astragalus* is represented with 29 native species, classified into 8 subgenera;^2,3^ several Bulgarian *Astragalus* species are endemic and protected by law.^4^

In this paper, we describe the phytochemical study of *Astragalus thracicus* Griseb, a tertiary relict and a Balkan peninsula endemic species. Nowadays, only three habitats of *A. thracicus* are known in Bulgaria: around the cities Sliven, Yambol, and Haskovo, but it also occurs in some limited areas in the Greek and Turkish regions of Thrace. This plant is a xeromorphic shrub with robust roots, densely branched, hairy stems (16–40 cm in height), and leaves that terminate with a spine.^5^

The phytochemical investigations of the *Astragalus* species described the presence of flavonoids, saponins, polysaccharides, and alkaloids. Major groups of described flavonoids are flavones, flavonols, flavanones, isoflavones, and isoflavans, both in the form of aglycones and glycosides.^6,7^ Common aglycons distributed in the *Astragalus* species are kaempferol and quercetin. 3′-*O*-Methoxyquercetin (syn. isorhamnetin) is frequently found, while 4′-*O*-methoxyquercetin (syn. tamarixetin) is rarely found in nature. Tamarixetin is a structural base of only 13 isolated natural compounds according to the PubChem database (July 2023); in genus *Astragalus,* it has been isolated only from *Astragalus miser var. oblongifolius* (Rydb.) Cronq.^8^ and *Astragalus armatus* Willd.^9^ The present study reports five compounds with tamarixetin aglycon; two of them are even further exotic due to their acylation with 3-hydroxy-3-methylglutaric acid (HMGA). The HMG moiety, itself, is considered as a rarely observed substituent in the plant kingdom (less than 50 reported compounds contain it), and HMG-acylated flavonoids were considered as a chemotaxonomic marker only for genus *Rosa* (Rosaceae).^10^ Surprisingly, in genus *Astragalus*, such flavonoids are described in *Astragalus monspessulanus* L.,^11^*Astragalus caprinus* Maire.,^12^*Astragalus gombiformis* Pomel.,^13^*A. spruneri* Boiss.,^14^ and *A. thracicus* Griseb.^15^*A. thracicus* was further investigated for the production of cycloartane saponins.^16^

Collagenase and elastase are enzymes that belong to a group of enzymes called matrix metalloproteinases (MMPs). Collagenase disrupts the collagen network in connective tissues, while elastin breaks elastin fibers. These effects lead to loss of skin elasticity, appearance of wrinkles, and subsequently participate in skin aging.^17^

One of the external factors aggravating skin aging is exposure to UV through sun light. The reactive oxygen species (ROS), generated after the absorption of UV light, damage lipid membranes and DNA directly, promoting wrinkle formation. Indirectly, the ROS increase the expression of MMP enzymes, further worsening the condition.^18,19^ Moreover, skin aging is connected to inflammatory conditions, and thus, the term “inflammaging” was coined.^20^ For example, a pro-inflammatory cytokine interleukin-1β is able to increase expression of collagenase in fibroblasts.^21^

As a part of our ongoing research on natural phenolic compounds with anti-inflammatory activity, we extracted and isolated a series of flavonoids from *A. thracicus*. Consequently, we tested the in vitro anticollagenase activity of selected *Astragalus* flavonoids and analyzed their anticollagenase and antielastase activity in silico.

## Results and Discussion

The structural analysis identified the isolated compounds as kaempferol-3-*O*-α-l-rhamnopyranosyl-(1 → 2)-[6-*O*-(3-hydroxy-3-methylglutaryl)]-β-d-galactopyranoside (**1**),^13^ tamarixetin-3-*O*-α-l-rhamnopyranosyl-(1 → 2)-[6-*O*-(3-hydroxy-3-methylglutaryl)]-β-d-galactopyranoside (**2**), tamarixetin-3-*O*-(2,6-di-*O*-α-l-rhamnopyranosyl)-β-d-galactopyranoside (**3**), tamarixetin 3-*O*-β-d-apiofuranosyl-(1 → 2)-β-d-galactopyranoside (**4**), tamarixetin-3-*O*-β-d-apiofuranosyl-(1 → 2)-[6-*O*-(3-hydroxy-3-methylglutaryl)]-β-d-galactopyranoside (**5**), and tamarixetin-3-*O*-β-d-apiofuranosyl-(1 → 2)-[α-l-rhamnopyranosyl-(1 → 6)]-β-d-galactopyranoside (**6**). Compounds **2**–**6** were isolated from a natural source for the first time. Selected compounds, obtained in sufficient amounts, together with other flavonoid glycosides obtained from *Astragalus* spp. were tested to evaluate the anticollagenase activity. The effect on collagenase and elastase was further evaluated by an in silico study. Since tamarixetin itself is known for its antioxidant and anti-inflammatory effect,^22^ the activities evaluated in this study would make it a promising antiaging natural product.

**Figure 1:**
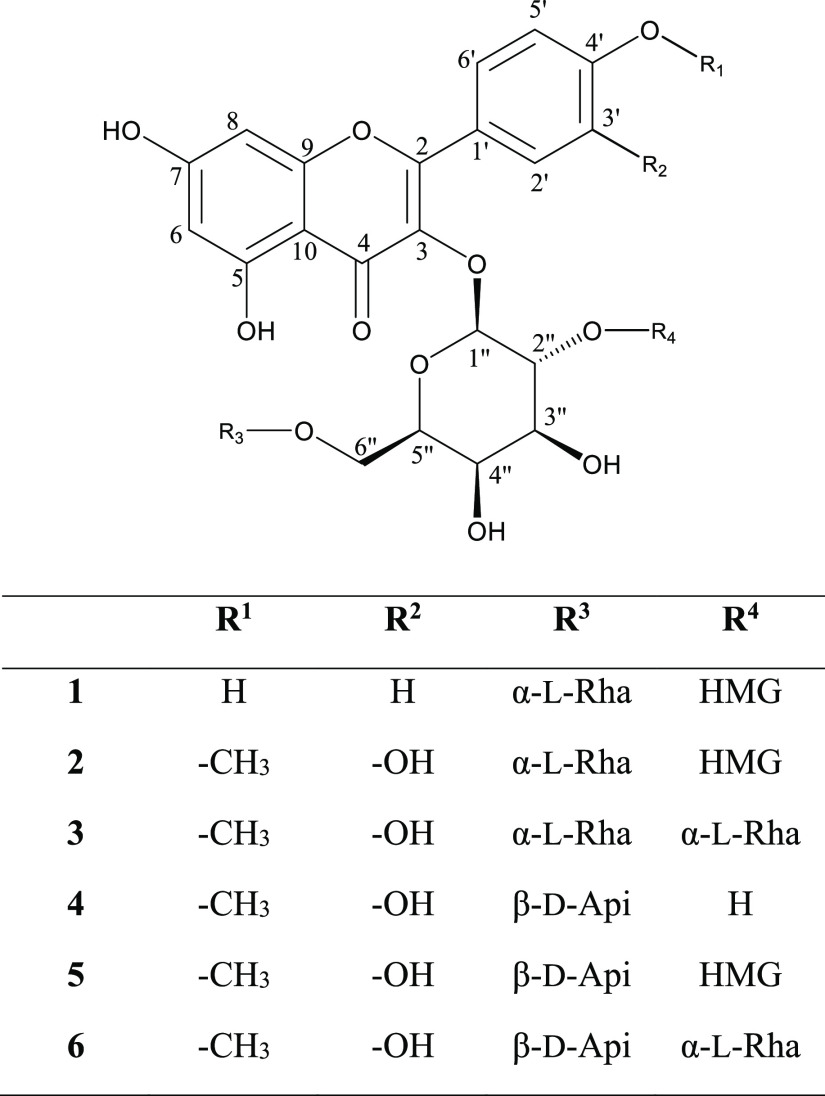
Structures of compounds **1**–**6**.

All compounds **1**–**6** (Figure 1) were isolated from the methanol extract of the aerial parts of *A. thracicus* Griseb. in the form of yellow amorphous powder. Their structures were determined by high-resolution electrospray ionization mass spectrometry (HRESIMS) and nuclear magnetic resonance (NMR) [^1^H, ^13^C, ^1^H-correlation spectroscopy (^1^H–^1^H COSY), heteronuclear single quantum coherence spectroscopy (HSQC), and heteronuclear multiple bond correlation (HMBC) spectral analysis].

The initial stage of analysis gave an idea of the flavonoid character of the compounds from the fluorescence of the spots, observed on thin-layer chromatography (TLC) plates at 253 and 366 nm. According to their UV spectra obtained from high-performance liquid chromatography with photodiode-array detection (HPLC-DAD) (from peak apex; λ_max_ 255 nm, λ_max_ 266 nm, and λ_max_ 353 nm), they were classified to be flavonols.^23^ Additional TLC analysis of the aglycons (after acid hydrolysis) referred to the presence of tamarixetin as aglycon of compounds **2**–**6** and kaempferol of compound **1**, respectively.

The negative HRESIMS of compound **2** showed a molecular ion peak [M – H]^−^ at *m*/*z* 767.2044 (calcd [M – H]^−^*m*/*z* 767.2040), suggesting the molecular formula C_34_H_40_O_20_ (formula weight 768.6694). A characteristic ion at *m*/*z* 623.1605, corresponding to [tamarixetin + galactose + rhamnose]^−^ with a mass loss of *m*/*z* 144.0439, was observed in the MS spectrum. Alongside, two important ion peaks were observed as a result of the breaking of the glycosidic bond: *m*/*z* 315.0526, corresponding to the tamarixetin aglycon and *m*/*z* 300.0273, corresponding to [tamarixetin –CH_3_]^−^, i.e., quercetin.

The ^1^H NMR spectrum (Table 1) showed signals following the ^1^H NMR tamarixetin pattern:^24^ five aromatic protons δ_H_ 6.18 (1H, d, *J* = 2.39 Hz, H-6) and δ_H_ 6.39 (1H, d, *J* = 2.39 Hz, H-8) (ring A), δ_H_ 7.64 (1H, d, *J* = 2.22 Hz, H-2′), δ_H_ 7.04 (1H, d, *J* = 8.67 Hz, H-5′), and δ_H_ 7.71 (1H, dd, *J* = 2.22; 8.67 Hz, H-6′) (ring B). The methoxy group at C-4′ was confirmed by a singlet at δ_H_ 3.92 (δ_C_ 54.9). Additionally, two anomeric proton signals at δ_H_ 5.66 (1H, d, *J* = 7.78 Hz) and δ_H_ 5.20 (1H, d, *J* = 1.47 Hz), two methyl groups at δ_H_ 0.95 (3H, d, *J* = 6.19 Hz) and δ_H_ 1.14 (3H, s), and six methylene protons δ_H_ 4.13 (1H, dd) and δ_H_ 4.17 (1H, dd), δ_H_ 2.41 (1H, d, *J* = 14.18 Hz) and δ_H_ 2.50 (1H, d, *J* = 14.49 Hz), δ_H_ 2.37 (1H, d, *J* = 15.20 Hz) and δ_H_ 2.44 (1H, d, *J* = 15.20 Hz) were observed.

**Table 1:** ^1^H and ^13^C NMR Chemical Shifts (δ_C_ in ppm) of Compounds **1–3** at 303 K

1			2		3	
position	δ_C_, type	δ_H_ (*J* in Hz)	δ_C_, type	δ_H_ (*J* in Hz)	δ_C_, type	δ_H_ (*J* in Hz)
**2**	157.2, C		156.7, C		156.7, C	
**3**	133.0, C		133.4, C	–OH	133.5, C	–OH
**4**	178.00, C		177.9, C		177.9, C	
**5**	161.7, C		161.7, C	–OH	161.7, C	–OH
**6**	98.4, CH	6.18, d (2.05)	98.3, CH	6.18, d (2.39)	98.4, CH	6.17, d (2.17)
**7**	164.2, C		164.3, C	–OH	164.5, C	–OH
**8**	93.3, CH	6.39, d (2.11)	93.2, CH	6.39, d (2.39)	93.2, CH	6.36, d (2.07)
**9**	157.0, C		156.9, C		156.6, C	
**10**	104.5, C		104.8, C		104.5, C	
**1′**	121.6, C		123.1, C		123.2, C	
**2′**	130.8, CH	8.06, m (8.76)	115.4, CH	7.64, d (2.22)	115.5, CH	7.64, d (2.20)
**3′**	114.8, CH	6.88, m (8.76)	145.7, CH	–OH	146.7, CH	–OH
**4′**	159.9, C	–OH	150.0, C	-OCH_3_	150.0, C	-OCH_3_
**5′**	114.8, CH	6.88, m (8.76)	110.7, CH	7.04, d (8.67)	110.7, CH	7.03, d (8.69)
**6′**	130.7, CH	8.06, m (8.76)	121.7 CH	7.71, dd (2.22; 8.67)	121.7 CH	7.71, dd (2.24; 8.62)
**–OCH**_**3**_			54.9, CH_3_	3.92 s	54.9, CH_3_	3.92, s
**3-***O***-gal**						
**1″**	99.1, CH	5.64, d (7.76)	99.4, CH	5.66, d (7.78)	99.7, CH	5.63, d (7.79)
**2″**	76.1, CH	3.92, dd	76.0, CH	3.95, dd	76.0, CH	3.94, dd
**3″**	74.1, CH	3.70 dd	74.1, CH	3.71 dd	74.3, CH	3.70 dd
**4″**	69.3, CH	3.77 dd	69.4, CH	3.81 dd	70.9, CH	3.78 dd
**5″**	73.0, CH	3.71 d *	73.0, CH	3.73 d*	73.9, CH	3.64 dt
**6″**	63.1, CH_2_	4.11 dd	63.0, CH_2_	4.13 dd	65.7, CH_2_	3.45 dd
		4.17 dd		4.17 dd		3.73 dd
**Rha (1** → **2**″**)**						
**1‴**	101.2, CH	5.20, d (1.60)	101.2, CH	5.20, d (1.47)	101.1, CH	5.20, d (1.62)
**2‴**	71.0, CH	3.97, dd	71.0, CH	3.98, dd	71.0, CH	3.98, dd
**3‴**	70.9, CH	3.71 dd	70.9, CH	3.78 dd	69.4, CH	3.79, dd
**4‴**	72.7, CH	3.33, pt (9.57)	72.7, CH	3.33, pt (9.57)	72.6, CH	3.32, pt (9.30)
**5‴**	68.4, CH	4.03, dq	68.4, CH	4.03, dq	68.4, CH	4.04, dq
**6‴**	16.1, CH_3_	0.97 d (6.23)	15.9, CH_3_	0.95 d (6.19)	16.0, CH_3_	0.94, d (6.23)
**HMG (1** → **6**″**)**						
**1**	170.7, C		170.7, C			
**2**	44.3, CH2	2.40, d (14.94)	44.8, CH_2_	2.41, d (14.18)		
		2.50, d (14.94)		2.50, d (14.49)		
**3**	69.1, C		69.1, C			
**4**	44.8, CH2	2.37, d (15.27)	44.3, CH_2_	2.37, d (15.20)		
		2.44, d (15.23)		2.44, d (15.20)		
**5**	173.6, C		173.8, C			
**3′**	26.2, CH_3_	1.14, s	26.2, CH_3_	1.14, s		
**Rha (1** → **6**″**)**						
**1′‴**					100.4, CH	4.52, d (1.67)
**2′‴**					70.7, CH	3.56, dd
**3′‴**					70.9, CH	3.49 dd
**4′‴**					72.4, CH	3.26, pt (9.50)
**5′‴**					68.3, CH	3.51, dq
**6′‴**					16.5, CH_3_	1.16 d (6.40)

^13^C and HSQC spectra of **2** showed the signals of 34 carbons, including three carbonyl carbons at δ_C_ 170.7, 173.8, and 177.9 ppm; ten nonprotonated carbon atoms at δ_C_ 69.1, 104.8, 123.1, 133.4, 145.7, 150.0, 156.7, 156.9, 161.7, and 164.3 ppm; 15 methines at δ_C_ 68.4, 69.4, 70.9, 71.0, 72.7, 73.0, 74.1, 76.0, 93.2, 98.3, 99.4, 101.2, 110.7, 115.4, and 121.7 ppm; three methylenes at δ_C_ 44.3, 44.8, and 63.0 ppm, and three methyl carbons δ_C_ at 15.9, 26.2, and 54.9 ppm. Five signals δ_C_ 133.4 (C-3), δ_C_ 161.7 (C-5), δ_C_ 164.3 (C-7), δ_C_ 145.7 (C-3′), and δ_C_ 150.0 (C-4′), corresponding to hydroxylated carbon atoms, were found in the ^13^C spectrum. A strongly shifted signal at δ_C_ 177.9 characterized the carbonyl group at C-4.

Analysis of the HMBC spectrum showed the following correlations: proton H-2′ strongly coupling with C-2, C-3′, C-4′, and C-6′ and a weak one with C-1′. Proton H-5′ showed strong coupling correlation to C-1′, C-3′, and C-4′ and a weak coupling correlation to C-2′. Proton H-6′ showed strong coupling with C-2, C-2′, and C-4′. Furthermore, H-6 showed a strong coupling correlation to C-5, C-7, C-8, and C-10. Proton H-8 showed a strong coupling correlation to C-6, C-7, C-9, and C-10 and a weak one to C-4. The singlet at δ_H_ 3.92, integrating for three protons, comprising a methyl group, showed a strong HMBC correlation to δ_C_ 150.0, was assigned as C-4′. A weak interaction signal was observed between galactose anomeric proton δ_H_ 5.66 and C-3 (δ_C_ 133.4) due to the free rotation of this bond. Based on the value of the chemical shift δ_C_ 133.4, we can conclude that the glycosylation of the tamarixetin aglycon occurs at this position, making the compound a 3-*O*-glycoside.

The overall MS and NMR evaluation of compound **2** showed tamarixetin aglycon substituted at the 3-*O*-position with one hexose, one deoxyhexose, and a 3-hydroxy-3-methylglutaroyl (HMG) group. Acid hydrolysis of **2** confirmed the presence of β-d-galactopyranose and α-l-rhamnopyranose. The second anomeric proton δ_H_ 5.20 showed HMBC correlation with δ_C_ 76.0 (C-2″), confirming the (1 → 2) connection. Finally, the two methylenes at δ_C_ 44.3 and δ_C_ 44.8, two carboxyl carbons at δ_C_ 170.7 and δ_C_ 173.8, one quaternary carbon atom at δ_C_ 69.1, and a methyl group at δ_C_ 26.2 belonging to a nonsugar substituent were determined to form a 3-hydroxy-3-methylglutaryl residue. HMBC confirmation came from the signal of carbonyl C-1 of HMG, correlating to the H-6a″/6b″ of the galactose. This clearly confirms the (1 → 6) connection between the HMG residue and the galactose. Compound **2** was characterized as tamarixetin-3-*O*-α-l-rhamnopyranosyl-(1 → 2)-[6-*O*-(3-hydroxy-3-methylglutaryl)]-β-d-galactopyranoside, representing a new natural product.

The negative HRESIMS of **1** gave a molecular ion peak [M – H]^−^ at *m*/*z* 737.1937 (calcd [M −H]^−^ 737.1935), suggesting the molecular formula C_33_H_38_O_19_ (formula weight 738.6434). A characteristic ion *m*/*z* 593.1485, corresponding to a mass loss of *m*/*z* 144.0451 giving rise to [kaempferol + galactose + rhamnose]^−^, was present. Alongside, two important ion peaks were observed after breaking of the glycosidic connection: *m*/*z* 284.0337, corresponding to the kaempferol itself and *m*/*z* 469.7081, corresponding to the sugars.

The NMR spectrum of compound **1** (Table 1) showed a very similar pattern to that of **2**, i.e., two sugar units (β-d-galactose and α-l-rhamnose) acylated with 3-hydroxy-3-methylglutaric acid. The observed differences were in the signals assigned from ring B: instead of a methoxy group at C-4′ (compound **2**), just a hydroxy group was observed in **1**, and instead of a hydroxylated C-3′ in **2**, in **1**, it is simply protonated. These differences make the structure of ring B symmetric, which results in the presence of the AA′BB′ system,^25^ characteristic of *p*-substituted aromatics.

After complete analysis of the spectra, compound **1** was characterized as kaempferol-3-*O*-α-l-rhamnopyranosyl-(1 → 2)-[6-*O*-(3-hydroxy-3-methylglutaryl)]-β-d-galactopyranoside. This compound was previously obtained from *A. gombiformis*,^13^ and it was identified as the content compound of *A. thracicus* for the first time.

In accordance with an initial TLC check-up and comparison with standards after acid hydrolysis, compound **3** was shown to be composed of tamarixetin aglycon and two sugars: galactose and rhamnose. The observed difference between compounds **2** and **3** is an HMG residue, bonded to the C-6″ position in **2** and a rhamnosyl moiety, found on the same C-6″ position in compound **3** (see Table 1). Characteristic signals for the (1 → 6) connected rhamnose are anomeric proton δ_H_ 4.52, (d, *J* = 1.67 Hz), bonded to δ_C_ 100.4, and its methyl radical at C-6**′‴** (δ_H_ 1.16; δ_C_ 16.5). The coupling constant of the anomeric proton was 1.67 Hz, which corresponds to the α-form. After complete assignment of signals, compound **3** was identified as tamarixetin-3-*O*-(2,6-di-*O*-α-l-rhamnopyranosyl)-β-d-galactopyranoside, which to the best of our knowledge is a new natural product. This was confirmed by HRESIMS, where we found a molecular ion [M – H]^−^*m*/*z* 769.2196 (calcd [M – H]^−^ 769.2196), corresponding to molecular formula C_34_H_42_O_20_ (formula weight 770.6852). Loss of *m*/*z* 14.0150 led to [M – H–CH_3_]^−^*m*/*z* 755.2045. In parallel, signals at *m*/*z* 315.0532 and *m*/*z* 300.0296 confirmed the same loss, which corresponded to the cleavage of methyl from C-4′ in the aglycon tamarixetin. An ion with *m*/*z* 623.1665 was detected, corresponding to [tamarixetin + galactose + rhamnose]^−^ after a loss of *m*/*z* 146.0538 equal to a loss of the rhamnose residue.

The initial TLC check-up of compounds **4**–**6** after acid hydrolysis showed the presence of tamarixetin, galactose, and apiose in compounds **4** and **5** and additional rhamnose for compound **6**. Compounds **4**, **5**, and **6** showed very similar structures according to the analysis of HRESIMS and NMR (^1^H, ^13^C, COSY, HSQC, and HMBC, Table 2). Compound **4**, determined as tamarixetin 3-*O*-β-d-apiofuranosyl-(1 → 2)-β-d-galactopyranoside, gave a structural base for building up compounds **5** and **6**. Compound **5** showed a 3-hydroxy-3-methylglutaryl moiety, attached to the hydroxyl on the C-6″ position, while compound **6** showed an α-l-rhamnosyl moiety on the same position, instead. Due to the close similarities between these three compounds, their spectral characteristics will be discussed in parallel.

**Table 2:** ^1^H and ^13^C NMR Chemical Shifts (δ_C_ in ppm) of Compounds **4–6** at 303 K

4			5 (in DMSO-*d*_6_)			6
**position**	**δ**_**C**_**, type**	**δ**_**H**_**(*J* in Hz)**	**δ**_**C**_**, type**	**δ**_**H**_**(*J* in Hz)**	**δ**_**C**_**, type**	**δ**_**H**_**(*J* in Hz)**
**2**	156.4, C		156.6, C		156.6, C	
**3**	133.9, C		133.8, C		133.8, C	
**4**	178.1, C		177.5, C		178.6, C	
**5**	161.8, C		161.6, C		161.7, C	
**6**	98.4, CH	6.16, d (1.72)	99.3, CH	6.16, d (2.05)	98.4, CH	6.18, d (2.08)
**7**	164.5, C		165.4, C		164.5, C	
**8**	93.2, CH	6.36, d (1.57)	94.0, CH	6.36, d (2.11)	93.3, CH	6.37, d (2.18)
**9**	156.9, C		156.3, C		157.0, C	
**10**	104.4, C		103.9, C		104.4, C	
**1′**	123.1, C		123.1, C		123.1, C	
**2′**	115.3, CH	7.65, d (2.10)	115.4, CH	7.47, d (2.18)	115.4, CH	7.65, d (2.26)
**3′**	145.1, CH	–OH	146.7, CH	–OH	145.7, CH	–OH
**4′**	150.1, C	–OCH_3_	150.4, C	–OCH_3_	150.1, C	–OCH_3_
**5′**	110.7, CH	7.02, d (8.67)	111.7, CH	6.94, d (8.76)	110.7, CH	7.04, d (8.65)
**6′**	121.8 CH	7.77, dd (2.10; 8.62)	122.2 CH	7.79, dd (2.21; 8.58)	121.9 CH	7.76, dd (2.23; 8.59)
**-OCH**_**3**_	55.0, CH_3_	3.92, s	55.9, CH_3_	3.83, s	54.9, CH_3_	3.92, s
**3-***O***-gal**						
**1″**	99.9, CH	5.46, d (7.39)	99.4, CH	5.45, d (7.74)	100.1, CH	5.38, d (7.71)
**2″**	75.5, CH	3.96, dd	75.0, CH	3.74, dd	75.4, CH	3.93, dd
**3″**	73.9, CH	3.68 dd	72.9, CH	3.58, dd	73.7, CH	3.65, dd
**4″**	69.2, CH	3.83, dd	69.1, CH	3.58, dd	69.2, CH	3.75, dd
**5″**	75.5, CH	3.46 t*	73.8, CH	3.58, dd	73.9, CH	3.59, dd
**6″**	60.6, CH_2_	3.54 dd	63.6, CH_2_	3.87, dd	65.7, CH_2_	3.40, dd
		3.60 dd		3.90, dd		3.69, dd
**Api (1** → **2**″**)**						
**1‴**	109.3, CH	5.45, d (1.67)	109.3, CH	5.29, d (1.49)	109.3, CH	5.44, d (1.70)
**2‴**	76.7, CH	4.08, d (1.65)	76.5, CH	3.80, d (1.40)	76.6, CH	4.04, d (1.60)
**3‴**	79.5, C	–OH	79.7, C	–OH	79.5, C	–OH
**4‴**	74.0, CH_2_	4.05, d (9.63)	74.4, CH_2_	3.51, d (9.19)	74.0, CH_2_	3.68, d (9.60)
		3.70, d (9.53)		3.85, d (9.70)		4.05, d (9.60)
**5‴**	64.7, CH_2_	3.73, d (11.46)	64.8, CH_2_	3.38, d (11.24)	64.7, CH_2_	3.62, d (11.47)
		3.63, d (11.46)		3.49, d (11.24)		3.73, d (11.47)
**HMG (1** → **6**″**)**						
**1**			170.6, C			
**2**			46.5, CH_2_	1.99, d (15.16)		
				2.13, d (15.30)		
**3**			69.2, C			
**4**			46.7, CH_2_	2.15, d (13.60)		
				2.24, d (13.70)		
**5**			175.9, C			
**3′**			27.7, CH_3_	0.92, s		
**Rha (1** → **6**″**)**						
**1′‴**					100.4, CH	4.50, d (1.71)
**2′‴**					70.7, CH	3.55, dd
**3′‴**					70.9, CH	3.48, dd
**4′‴**					72.4, CH	3.25, pt (9.49)
**5′‴**					68.2, CH	3.49, dq
**6′‴**					16.5, CH_3_	1.15, d (6.23)

The HRESIMS spectrum of compound **4** showed molecular ion [M – H]^−^*m*/*z* 609.1459 (calcd [M – H]^−^ 609.1461; formula weight 610.5175), suggesting the molecular formula C_27_H_30_O_16_. A split between galactose and apiose (*m*/*z* loss of 132.0366) resulted in the peak observed at *m*/*z* 477.1034, and cleavage of bond between aglycon and sugars (−3–*O*– bond) was confirmed by ion *m*/*z* 294.8727 [galactose + apiose] and *m*/*z* 315.0534 [tamarixetin], which was further cleaved to [tamarixetin –CH_3_]^−^*m*/*z* 300.0298 [*syn*. quercetin]^−^. The HRESIMS spectrum of compound **5** showed a molecular ion with [M – H]^−^*m*/*z* 753.1886 (calcd [M – H]^−^ 753.1884; formula weight 754.6428 for C_33_H_38_O_20_). A characteristic ion *m*/*z* 609.1399, corresponding to [tamarixetin + galactose + apiose]^−^, was observed in the MS spectrum with a mass loss of *m*/*z* 144.0463 due to the cleavage of the HMG moiety. Three important ion peaks were observed: *m*/*z* = 438.9658 corresponding to the sugars, *m*/*z* = 315.0526 corresponding to tamarixetin aglycon, and *m*/*z* = 300.0273 corresponding to [tamarixetin – CH_3_]^−^. Compound **6** showed [M – H]^−^*m*/*z* 755.2093 (calcd [M – H]^−^ 755.2040; formula weight 756.6587 for C_33_H_40_O_20_). Peaks at *m*/*z* 623.1665 resulting from the loss of the apiose (*m*/*z* 132.0428) and *m*/*z* 593.9035 resulting from rhamnose residue loss (*m*/*z* 161.3058) were significant. The peaks corresponding to the aglycon (*m*/*z* 315.0532) and demethylated aglycon (*m*/*z* 300.0295) can be easily found in the spectrum.

All three aglycons followed the same ^1^H and ^13^C NMR pattern for tamarixetin, as it was described above (data shown in Table 2). All the spectral characteristics lead us to the conclusion that these compounds are tamarixetin-3-*O*-glycosides. It is important to note that some of the values of compound **5** slightly differed from others due to the DMSO being used as the solvent in the NMR measurements.

Finally, compounds **4**, **5**, and **6** were identified as tamarixetin 3-*O*-β-d-apiofuranosyl-(1 → 2)-β-d-galactopyranoside (**4**), tamarixetin-3-*O*-β-d-apiofuranosyl-(1 → 2)-[6-*O*-(3-hydroxy-3-methylglutaryl)]-β-d-galactopyranoside (**5**), and tamarixetin-3-*O*-β-d-apiofuranosyl-(1 → 2)-[α-l-rhamnopyranosyl-(1 → 6)]-β-d-galactopyranoside (**6**), all newly identified natural products.

**Figure 2:**
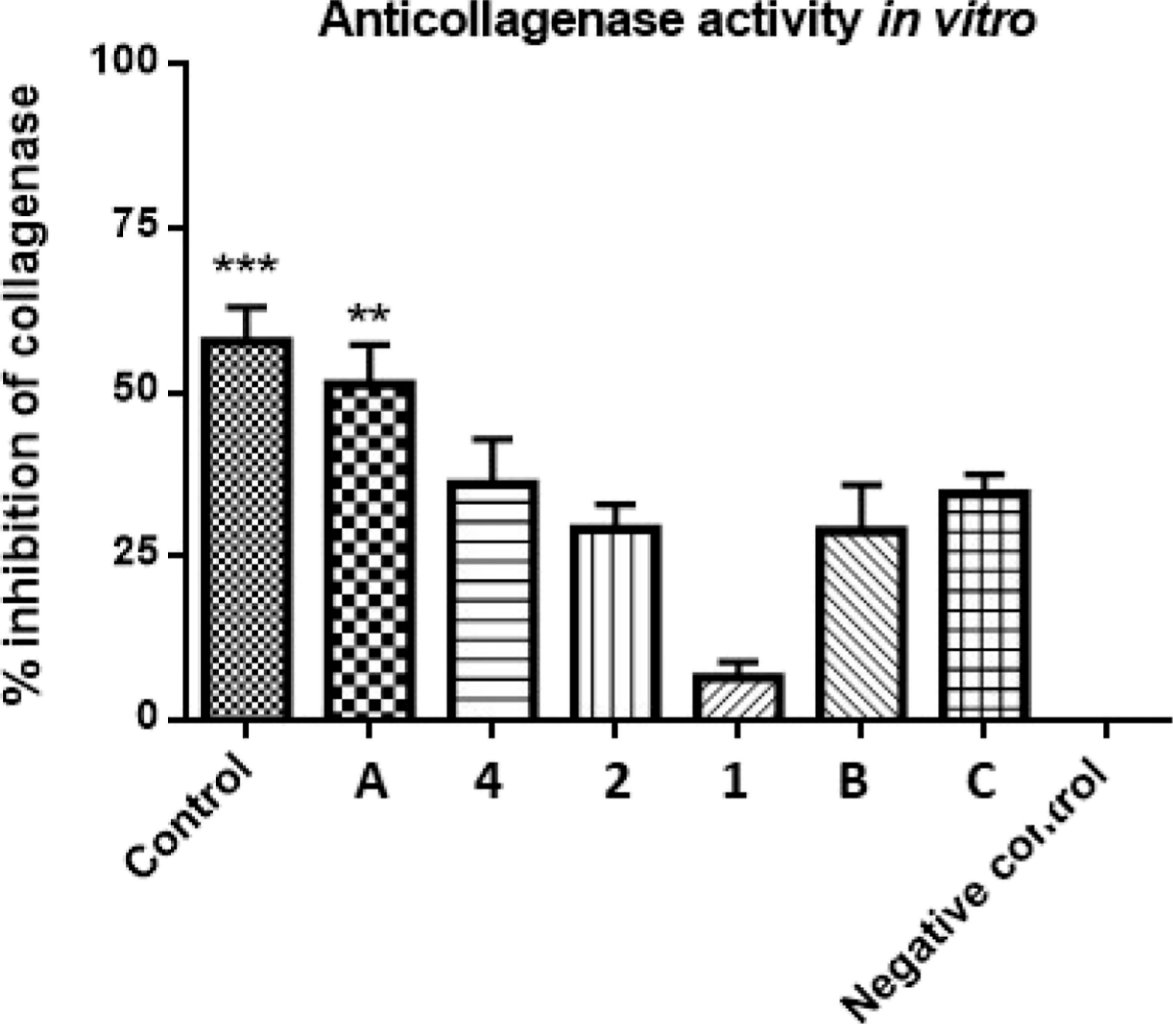
In vitro measurement of anticollagenase activity of compounds **1**, **2** and **4**, and kaempferol-3-*O*-β-d-apiofuranosyl-(1 → 2)-β-d-galactopyranoside (**C**), quercetin-3-*O*-β-d-apiofuranosyl-(1 → 2)-β-d-galactopyranoside (**A**), and kaempferol 3-*O*-[β-d-glucopyranosyl-(1 → 4)-α-l-rhamnopyranosyl-(1 → 6)-(2″-3-hydroxy-3-methylgutaroyl)-(β-d-galactopyranoside)] (**B**).^15^ Values of *p* lower than 0.05 were considered statistically significant; *** indicates a significant difference *p* < 0.001; control—chlorogenic acid; negative control (NC)—DMSO.

Compounds **1**, **2**, and **4**, together with kaempferol-3-*O*-β-d-apiofuranosyl-(1 → 2)-β-d-galactopyranoside, quercetin-3-*O*-β-d-apiofuranosyl-(1 → 2)-β-d-galactopyranoside, and kaempferol 3-*O*-[β-d-glucopyranosyl-(1 → 4)-α-l-rhamnopyranosyl-(1 → 6)-(2″-3-hydroxy-3-methylgutaroyl)-(β-d-galactopyranoside)],^15^ were further tested for their anticollagenase activity in an in vitro experiment (Figure 2). Chlorogenic acid was used as a positive control, showing 57.8 ± 4.5% collagenase inhibition. The greatest effect was shown for quercetin-3-*O*-β-d-apiofuranosyl-(1 → 2)-β-d-galactopyranoside, possibly due to the presence of an *ortho*-dihydroxy arrangement of flavonoid ring B. Compound **4** showed activity measured as 36.0 ± 6.9%; compound **2** showed slightly lower values (29.3 ± 3.7%). Both compounds share the same aglycon, i.e., tamarixetin. A significantly lower degree of inhibition was observed in the measurement of the kaempferol glycoside (**1**), which equals 6.6 ± 2.0% anticollagenase activity. Data for **3**, **5**, and **6** are not available due to the insufficient quantity of test compounds for the assay.

Furthermore, to elucidate the effects on collagenase and additionally on elastase, we performed docking experiments in silico. Both collagenase and elastase are proteolytic enzymes. Since their substrates are also large molecules, the active site must be on the surface of the enzyme. This opens field for a wider variety of molecules to interact with the active site and thus compete with binding of the substrate.

The studied small molecules have a planar structure of aglycon and possess one or more sugar units with a high number of hydroxyl groups that can effectively form hydrogen bonds with a protein surface. Due to the molecular weight, number of hydrogen donors and acceptors, and hydrophilicity, the tested substances violate Lipinski’s rule of five peroral drugs.

**Figure 3:**
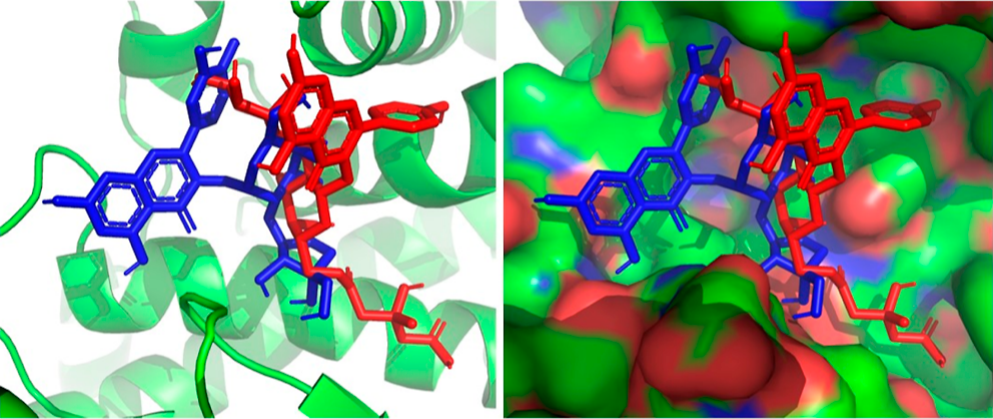
Structure overlay of compounds **1** (red) and **A** (blue) used in pdb entry 2Y6I (collagenase, http://www.rcsb.org) in target receptor pocket. The image elucidates the docking results of possible effective blocking of the active site by all tested compounds.

All tested substances showed a strong affinity to the target structures (Figure 3) and can act as a potential nonspecific competitive inhibitor. The binding energy for all structures was (Δ*G* < −6.4 kcal/mol) for collagenase and similarly (Δ*G* < −6.6 kcal/mol) for elastase. Computed values are shown in Tables S1 and S2 (Supporting Information). Similar docking results of isolated phenolic compounds were reported by Deniz et al.^26^ Differences in activity can be explained by the possible stronger binding to the enzyme surface outside the active binding site. All simulated substances violate Lipinski’s rule of five peroral drug for the molecular weight, number of hydrogen donors and acceptors, and lipophilicity. Even the tested compounds are not very suitable for oral administration due to violating Lipinski’s rule of five and possessing glycosidic bonds, which can be broken by hydrolytic enzymes. They still can be used effectively for topical administration or can be used as a template for a new group of effective compounds for accelerating the surface tissue healing process by blocking the tissue breaking process catalyzed by elastase or collagenase.

The group of enzymes assigned as collagenases catalyzes the cleavage breaking of the peptide bonds in collagen. Flavonoids, and especially the flavonols, may prevent collagen breakdown by inhibiting collagenase in inflamed skin as well as photoaged skin.^27^ There are numerous reports showing the plant extracts inhibiting collagenase, with flavonoids being assigned to be responsible for the activity.^28−30^ Also, isolated flavonoids showed the inhibitory effects on collagenase and elastase, given the connections with their possible anti-inflammatory and antiaging activities.^31−33^ For example, Shaji et al. documented the ability of tamarixetin to inhibit matrix metalloproteinase-9 (also known as 92 kDa type IV collagenase) via inhibition of the nuclear factor κB pathway.^34^ Further, inhibition of this cellular pathway leads to a decrease of expression of proinflammatory cytokines. Moreover, Rice-Evans et al. showed that tamarixetin is a potent antioxidant.^35^

The reported activity of tamarixetin derivatives showed that combined antioxidant, anti-inflammatory, and anticollagenase activity can be advantageously tested for topical administration and possible tissue regeneration and antiaging effect.

## Conclusions

The chromatographic separation of the extract from *A. thracicus* led to the isolation of several tamarixetin derivatives. Three of the new compounds were substituted with the untypical for the genus *Astragalus* 3-hydroxy-3-methylglutaryl residue (HMG). Selected compounds from *A. thracicus* showed anticollagenase activity, and the effect on collagenase and elastase was further proved by in silico study.

## Experimental Section

### General Experimental Procedures

NMR spectra were recorded using an Agilent DD2 600 MHz NMR instrument with 1D and 2D pulse sequences to obtain ^1^H, ^13^C, ^1^H–^1^H COSY, HMBC and HSQC, TOCSY, and NOESY spectra. The spectra were further processed with the software MestReNova 12.0.

HRESIMS spectra were recorded with the LC/FTMS system, containing an Orbitrap mass spectrometer and ESI ion source (Thermo Fisher, Scientific, Inc., Bremen, Germany), used in the mode of ultrahigh resolution (100,000) and UPLC system (Accela, Thermo Fisher, Scientific, Inc. Bremen, Germany). LC conditions: column Hypersil Gold C18 (50 × 2.1 mm, 1.9 μm; Thermo Fisher, Scientific, USA), column block temperature 30 °C, flow rate 0.3 mL/min; and mobile phase 0.1% HCOOH in MeOH (A) and 10 mM ammonium formate in 0.1% HCOOH (B). Gradient elution was as follows: starting conditions A 50%, 0.5 min 50% A, in 5.5 min 100% A, in 6.0 min 100% A, 6.1 min 50% A, and in 10.0 min 50% of A. ESI setup (negative mode): the capillary voltage 4.6 kV, temperature 250 °C, and N_2_ as nebulization and drying gas (50 and 5 L/min, respectively). The software used for the evaluation was Qual Browser; Thermo Xcalibur 3.1.66.10.

HPLC was done on an Agilent 1100 Series (degasser G1322A, quaternary pump G1311A, autosampler ALS G1313A, column compartment G1316, DAD G1315B, loop 20 μL, DAD 200–900 nm) with a Kinetex PFP 100A column (250 mm × 4.6 mm I.D., 5 μm; Phenomenex, CA, USA). The flow rate was 1 mL/min, with acetonitrile (A) and 0.2% HCOOH (B), from 10% A to 100% of A in 36th min, flowing by 100% A and reconditioning of the column.

Semipreparative HPLC was carried out using the Dionex UltiMate 3000 system (Pump Dionex UltiMate 3000 UPLC + Focused, Dionex UltiMate 3000 RS VariableWavelength Detector, fraction collector Dionex UltiMate 3000 with 6 positions, LCO 101 ECOM column oven, constant temperature 40 °C, autosampler Dionex UltiMate 3000, loop 100 μL), column Ascentis RP-AMIDE (250 mm × 10 mm, 5 μm; Supelco, PA, USA), and a flow rate of 5 mL/min. TLC was carried out on precoated silica gel plates (Kieselgel G, F_254_, 60, Merck, Darmstadt, Germany) with three different solvent systems: **S1** for glycosides EtOAc/MeOH/H_2_O (100:13.5:10, v/v/v), **S2** for aglycons CHCl_3_/MeOH (96:4, v/v), and **S3** for sugars EtOH/NH_3_/H_2_O (80:4:16, v/v). Spots of aglycons were visualized under UV light (366 nm) by spraying with NTS/PEG reagent, while sugars were visualized by spraying with aniline-phthalate solution and heated for 5 min at 105 °C. Column chromatography (CC) was performed using Diaion HP-20 (Ø = 80 mm, height 70 cm ∼700 g; Supelco, PA, USA), and silica gel (40–63 μm, Ø = 35 mm, height 60 cm; Sigma-Aldrich, St. Louis, MO, USA).

The chemicals used for the evaluation of bioactivity were as follows: collagenase from *Clostridium histolyticum* (0.8 mg/mL) dissolved in 50 mM Tricine buffer (Sigma-Aldrich), FALGPA (*N*-(3-[2-furyl]acryloyl)-Leu-Gly-Pro-Ala), chlorogenic acid used as a positive control, and DMSO (Sigma-Aldrich) used as an NC.

### Plant Material

The aerial plant parts from *A. thracicus* Griseb., in the blossoming stage, were collected in June 2013 from the habitat, located on Bakadzhitsite hills, close to Yambol (Google Maps coordinates: 42.452016 N, 26.663550 E). Fresh plant material was quickly dried in a shaded place at room temperature. The specimens are deposited in the Department of Pharmacognosy, Faculty of Pharmacy at the Medical University Sofia and in the Herbarium of Institute of Biodiversity and Ecosystem Research at the Bulgarian Academy of Sciences (SOM) with ref no. SOM001363 (Supporting Information, Figure S1).

### Extraction and Isolation

Plant material, used further in this study, consisted of 1100 g of well-dried and ground aerial parts from *A. thracicus*. The plant material was exhaustively extracted with 80% methanol under reflux (20 × 1.25 L) and evaporated to dryness under conditions of reduced pressure (Supporting Information, Figure S2). Crude extract was dissolved in water and defatted via liquid–liquid partitioning with CH_2_Cl_2_. The defatted water residue (63.5 g) was subjected to CC at atmospheric pressure on 700 g of Diaion HP-20 sorbent with a mobile phase H_2_O-MeOH with gradient increasing content of methanol (0 → 100%, v/v). 105 fractions of 500 mL were collected. The content of the fractions was individually monitored via TLC and HPLC, resulting in 6 combined fractions (A → F). Fraction C (eluted with 30% MeOH in distilled water) was subjected to CC on silica gel.

Rechromatography of fraction C: fraction C (weight 4.00 g) was separated on a silica gel column with a step-gradient of chloroform: methanol: water (v/v/v) in different ratios (9:1:0.1 → 8:2:0.2 → 7:3:0.3 → 6:4:0.4 →5:5:0.5). Thirty fractions were collected, each 125 mL. After TLC and HPLC analysis, the fractions were combined as follows: 1–2 (C_1_), 3–5 (C_2_—720 mg), 6–8 (C_3_—300 mg), 9–11 (C_4_—570 mg), 12–22 (C_5_—210 mg), 23–30 (C_6_—640 mg).

Fraction **C**_**2**_ was rechromatographed over silica gel chloroform: methanol: water (v/v/v) with step-gradient ratio 9:1:0.1 → 8:2:0.2 → 7:3:0.3 → 6:4:0.4 → 5:5:0.5. Twenty-one fractions were collected, 125 mL each, and combined as follows: 4–5 (C_2B_—125 mg), 6–7 (C_2C_—70 mg), 8–11 (C_2D_—155 mg), 12–13 (C_2E_—183 mg). Purification of **C**_**2B**_ fraction: semipreparative HPLC (gradient of 21–26% acetonitrile and 0.2% HCOOH for 25 min) and subsequent prep-TLC of the fifth collected peak (**5 C**_**2B**_) resulted in compound **4** (20 mg). Semipreparative HPLC was applied to **C**_**2D**_ fraction (gradient of 19–25% acetonitrile and 0.2% HCOOH for 25 min) gave pure compound **6** (3.8 mg). Under the same conditions, compound **3** (4.9 mg) was obtained from fraction **C**_**2E**_. Fraction **C**_**3**_ was applied to semipreparative HPLC (gradient of 23–23.5% acetonitrile and 0.2% HCOOH for 25 min), and after subsequent prep-TLC of the fifth collected peak (**5 C**_**3**_), compound **5** (40.6 mg) was obtained. Purification process of fraction **C**_**4**_ continued with semipreparative HPLC (gradient of 23–25% acetonitrile and 0.2% HCOOH for 25 min), resulting in pure compounds **1** (13 mg) and **2** (12 mg). Purity of compounds varied between 87 and 97.5% by HPLC.

### Anticollagenase Activity In Vitro

Reaction mixture (total volume 150 μL): 125 μL of collagenase from *Hathewayahistolytica*(formerly *C. histolyticum* (0.8 mg/mL) dissolved in 50 mM tricine buffer (Sigma-Aldrich) was mixed with 12.5 μL of tested sample (0.8 mg/mL) dissolved in DMSO. After 15 min of incubation at 25 °C, 12.5 μL of FALGPA (*N*-(3-[2-furyl]acryloyl)-Leu-Gly-Pro-Ala) (9.6 mM) dissolved in 50 mM Tricine buffer (Sigma-Aldrich) was added. The absorbance (λ 340 nm, 25 °C) of the tested sample (A sample) started to be measured exactly 1 min after the addition of the FALGPA and continued for 15 min, using a microplate reader (Synergy HT) in 96-well microtiter plates. The experiment was done in quadruplicate. Chlorogenic acid was used as a positive control and DMSO as an NC (A control). The blank solution (A blank) consisted of tricine buffer and tested sample or DMSO (in the case of a NC). The collagenase inhibitory activity was calculated according to the formula
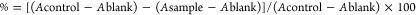


Statistical analysis was carried out using GraphPad Prism 6.01 software. Results are expressed as the mean ± standard deviation. Statistical analyses were performed using the nonparametric (*n* < 30) Kruskal–Wallis analysis, together with Dunn’s posthoc test. Values of p less than 0.05 were considered statistically significant; *n* = number of repetitions. ** indicates a significant difference *p* < 0.01; ***p < 0.001 vs NC - DMSO. Values are mean ± SD; *n* = 4.

### Molecular Docking

The ligand–protein interactions of collagenase and elastase enzyme were performed with AutoDockVina.^36^ Molecular dynamics used for receptor energy minimization were performed with NAMD using CUDA background.^37^ VMD GUI was used for protein preparation for molecular dynamics simulation for graphical evaluation of results.^38^

The three-dimensional (3D) structures of ligands were modeled using Marvin Sketch (https://www.chemaxon.com)^39^ and its minimized 3D structure exported as the PDB file. The 3D structure of receptors was downloaded from the RCSB protein database (http://www.rcsb.org). Collagenase PDB ID 2Y6I and elastase PDB ID 1BRU were selected for the in silico experiment.^40^

The procedure described previously^41^ was used for a protein-energy minimization calculation. NAMD software package with GPU background was applied for the acceleration of the computation process. Atom coordinates of receptor proteins from the crystallographic experiment were obtained from the RCSB Protein Data Bank with ID 2Y6I and 1BRU for Porcine pancreatic elastase and *C. histolyticum* collagenase type IA, respectively. For both protein chains, the energy was minimized by using molecular dynamics with the NAMD software package. The simulated system was neutralized, and the isosmotic environment was simulated with NaCl at a concentration of 0.15 mol/L. Minimization used 5000 steps.^41^ After minimization, all heteroatoms were removed from the file, and the proteins were subjected to docking using a PyRx docking tool with AutoDockVina with the exhaustiveness set to 50.

## Supporting Information Available

^1^H, ^13^C, COSY, HMBC, HSQC, HRESIMS, and HPLC spectra for compounds **1**–**6** and additional tables and figures (PDF)

## References

[ref1] Li X.; Qu L.; Dong Y.; Han L.; Liu E.; Fang S.; Zhang Y.; Wang T. A review of recent research progress on the Astragalus genus. Molecules 2014, 19 (11), 18850–18880. 10.3390/molecules191118850. 25407722 PMC6270929

[ref2] Tutin T. G. Flora Europaea; Cambridge University Press, 1968; Vol. 2.

[ref3] Platikanov S.; Nikolov S.; Pavlova D.; Evstatieva L.; Popov S. Volatiles from four Astragalus species: Phenological changes and their chemotaxonomical application. Z. Naturforsch., C: J. Biosci. 2005, 60 (7–8), 591–599. 10.1515/znc-2005-7-814. 16163835

[ref4] Petrova A.; Vladimirov V. Red List of Bulgarian vascular plants. Phytol. Balc. 2009, 15 (1), 63–94.

[ref5] Stoeva M. Red Data Book of the Republic of Bulgaria - Digital edition. Astracantha thracica: Red Data Book of Bulgaria. 2023, http://e-ecodb.bas.bg/rdb/en/vol1/Astthrac.html (accessed Nov 14, 2023).

[ref6] Pistelli L. F. Secondary metabolites of genus Astragalus: Structure and biological activity. In Studies in Natural Products Chemistry; Atta-ur-Rahman, Ed.; Bioactive Natural Products (Part H); Elsevier, 2002; Vol. 27, pp 443–545.

[ref7] Krasteva I.; Bratkov V.; Shkondrov A.; Zdraveva P. Flavonoids from the genus Astragalus: Phytochemistry and biological activity. Pharmacogn. Rev. 2016, 10 (19), 11–32. 10.4103/0973-7847.176550. 27041870 PMC4791984

[ref8] Norris F. A.; Stermitz F. R. 4′-O-methylquercetin 3-glucoside from *Astragalus miser* var. *oblongifolius*. Phytochemistry 1970, 9 (1), 229–230. 10.1016/S0031-9422(00)86635-2.

[ref9] Khalfallah A.; Karioti A.; Berrehal D.; Kabouche A.; Lucci M.; Kabouche Z.; Bilia A. Flavonoid triglycosides from *Astragalus armatus*. Planta Med. 2011, 77 (12), 47 10.1055/s-0031-1282531.

[ref10] Porter E. A.; Van Den Bos A. A.; Kite G. C.; Veitch N. C.; Simmonds M. S. J. Flavonol glycosides acylated with 3-hydroxy-3-methylglutaric acid as systematic characters in Rosa. Phytochemistry 2012, 81, 90–96. 10.1016/j.phytochem.2012.05.006. 22721781

[ref11] Krasteva I.; Bratkov V.; Bucar F.; Kunert O.; Kollroser M.; Kondeva-Burdina M.; Ionkova I. Flavoalkaloids and Flavonoids from *Astragalus monspessulanus*. J. Nat. Prod. 2015, 78 (11), 2565–2571. 10.1021/acs.jnatprod.5b00502. 26558405

[ref12] Semmar N.; Fenet B.; Gluchoff-Fiasson K.; Hasan A.; Jay M. Four New Flavonol Glycosides from the Leaves of *Astragalus caprinus*. J. Nat. Prod. 2002, 65 (4), 576–579. 10.1021/np010328l. 11975505

[ref13] Montoro P.; Teyeb H.; Masullo M.; Mari A.; Douki W.; Piacente S. LC-ESI-MS quali-quantitative determination of phenolic constituents in different parts of wild and cultivated *Astragalus gombiformis*. J. Pharm. Biomed. Anal. 2013, 72, 89–98. 10.1016/j.jpba.2012.09.014. 23146231

[ref14] Shkondrov A.; Krasteva I.; Bucar F.; Kunert O.; Kondeva-Burdina M.; Ionkova I. Flavonoids and saponins from two Bulgarian Astragalus species and their neuroprotective activity. Phytochem. Lett. 2018, 26, 44–49. 10.1016/j.phytol.2018.05.015.

[ref15] Vasilev H.; Šmejkal K.; Gronover C. S.; Choi Y. H.; Prüfer D.; Jankovská D.; Ionkova I. Flavonol glycosides from aerial parts of *Astragalus thracicus* Griseb. Phytochem. Lett. 2021, 41, 119–122. 10.1016/j.phytol.2020.11.012.

[ref16] Enchev P.; Zarev Y.; Michler H.; Ionkova I. Production of rare cycloartane saponins from *Astragalus thracicus* (Griseb) compared to *Astragalus membranaceus* (Fisch.) Bunge - native and biotechnological sources. Pharmacia 2023, 70, 73–77. 10.3897/pharmacia.70.e97782.

[ref17] Younis M. M.; Ayoub I. M.; Mostafa N. M.; El Hassab M. A.; Eldehna W. M.; Al-Rashood S. T.; Eldahshan O. A. GC/MS profiling, anti-collagenase, anti-elastase, anti-tyrosinase and anti-hyaluronidase activities of a *Stenocarpus sinuatus* leaves extract. Plants 2022, 11 (7), 918 10.3390/plants11070918. 35406898 PMC9002779

[ref18] Mukherjee P. K.; Maity N.; Nema N. K.; Sarkar B. K. Bioactive compounds from natural resources against skin aging. Phytomedicine 2011, 19 (1), 64–73. 10.1016/j.phymed.2011.10.003. 22115797

[ref19] Fisher G. J.; Voorhees J. J. Molecular mechanisms of photoaging and its prevention by retinoic acid: ultraviolet irradiation induces MAP kinase signal transduction cascades that induce Ap-1-regulated matrix metalloproteinases that degrade human skin in vivo. J. Invest. Dermatol. Symp. Proc. 1998, 3 (1), 61–68. 10.1038/jidsymp.1998.15. 9732061

[ref20] Pająk J.; Nowicka D.; Szepietowski J. C. Inflammaging and immunosenescence as part of skin aging - a narrative review. Int. J. Mol. Sci. 2023, 24 (9), 7784 10.3390/ijms24097784. 37175491 PMC10178737

[ref21] Vincenti M. P.; Coon C. I.; Lee O.; Brinckerhoff C. E. Regulation of collagenase gene expression by IL–1β requires transcriptional and post-transcriptional mechanisms. Nucleic Acids Res. 1994, 22 (22), 4818–4827. 10.1093/nar/22.22.4818. 7984435 PMC308536

[ref22] Lesjak M.; Beara I.; Simin N.; Pintać D.; Majkić T.; Bekvalac K.; Orčić D.; Mimica-Dukić N. Antioxidant and anti-inflammatory activities of quercetin and its derivatives. J. Funct.Foods 2018, 40, 68–75. 10.1016/j.jff.2017.10.047.

[ref23] Andersen O. M.; Markham K. R., Eds.; Flavonoids: Chemistry, Biochemistry and Applications, 1st ed.; CRC Press: Boca Raton, FL, 2005.

[ref24] Kunert O.; Alperth F.; Pabi E.; Bucar F. Highly oxidized flavones in Artemisia species - structure revisions and improved UHPLC-MS^n^ analysis. Heliyon 2023, 9 (11), e22309 10.1016/j.heliyon.2023.e22309. 38058631 PMC10696001

[ref25] Günther H. 1H-NMR Spectra of the AA′ XX′ and AA′ BB′ Type-Analysis and Classification. Angew. Chem. Int. Ed. 1972, 11 (10), 861–874. 10.1002/anie.197208611.

[ref26] Deniz F. S. S.; Salmas R. E.; Emerce E.; Cankaya I. I. T.; Yusufoglu H. S.; Orhan I. E. Evaluation of collagenase, elastase and tyrosinase inhibitory activities of *Cotinus coggygria* Scop. through *in vitro* and *in silico* approaches. S. Afr J. Bot. 2020, 132, 277–288. 10.1016/j.sajb.2020.05.017.

[ref27] Sin B. Y.; Kim H. P. Inhibition of collagenase by naturally-occurring flavonoids. Arch Pharm. Res. 2005, 28 (10), 1152–1155. 10.1007/BF02972978. 16276971

[ref28] Senol Deniz F. S.; Orhan I. E.; Duman H. Profiling cosmeceutical effects of various herbal extracts through elastase, collagenase, tyrosinase inhibitory and antioxidant assays. Phytochem. Lett. 2021, 45, 171–183. 10.1016/j.phytol.2021.08.019.

[ref29] Mandrone M.; Coqueiro A.; Poli F.; Antognoni F.; Choi Y. Identification of a collagenase-inhibiting flavonoid from *Alchemilla vulgaris* using NMR-based metabolomics. Planta Med. 2018, 84 (12/13), 941–946. 10.1055/a-0630-2079. 29797306

[ref30] Mandrone M.; Lorenzi B.; Venditti A.; Guarcini L.; Bianco A.; Sanna C.; Ballero M.; Poli F.; Antognoni F. Antioxidant and anti-collagenase activity of *Hypericum hircinum* L. Ind. Crop. Prod. 2015, 76, 402–408. 10.1016/j.indcrop.2015.07.012.

[ref31] Feldo M.; Wójciak M.; Ziemlewska A.; Dresler S.; Sowa I. Modulatory effect of diosmin and diosmetin on metalloproteinase activity and inflammatory mediators in human skin fibroblasts treated with lipopolysaccharide. Molecules 2022, 27 (13), 4264 10.3390/molecules27134264. 35807509 PMC9268213

[ref32] Lee K. E.; Bharadwaj S.; Yadava U.; Kang S. G. Computational and *in vitro* investigation of (−)-epicatechin and proanthocyanidin B2 as inhibitors of human matrix metalloproteinase 1. Biomolecules 2020, 10 (10), 1379 10.3390/biom10101379. 32998374 PMC7650666

[ref33] Lu W.; Zhu J.; Zou S.; Li X.; Huang J. The efficient expression of human fibroblast collagenase in *Escherichia coli* and the discovery of flavonoid inhibitors. J. Enzyme Inhib. Med. Chem. 2013, 28 (4), 741–746. 10.3109/14756366.2012.681650. 22524676

[ref34] Shaji S. K.; G D.; Sunilkumar D.; Pandurangan N.; Kumar G. B.; Nair B. G. Nuclear factor-κB plays an important role in Tamarixetin-mediated inhibition of matrix metalloproteinase-9 expression. Eur. J. Pharmacol. 2021, 893, 173808 10.1016/j.ejphar.2020.173808. 33345858

[ref35] Rice-Evans C. A.; Miller N. J.; Paganga G. Structure-antioxidant activity relationships of flavonoids and phenolic acids. Free Radic. Biol. Med. 1996, 20 (7), 933–956. 10.1016/0891-5849(95)02227-9. 8743980

[ref36] Trott O.; Olson A. J. AutoDock Vina: Improving the speed and accuracy of docking with a new scoring function, efficient optimization, and multithreading. J. Comput. Chem. 2010, 31 (2), 455–461. 10.1002/jcc.21334. 19499576 PMC3041641

[ref37] Phillips J. C.; Braun R.; Wang W.; Gumbart J.; Tajkhorshid E.; Villa E.; Chipot C.; Skeel R. D.; Kalé L.; Schulten K. Scalable molecular dynamics with NAMD. J. Comput. Chem. 2005, 26 (16), 1781–1802. 10.1002/jcc.20289. 16222654 PMC2486339

[ref38] Humphrey W.; Dalke A.; Schulten K. VMD: Visual molecular dynamics. J. Mol. Graph. 1996, 14 (1), 33–38. 10.1016/0263-7855(96)00018-5. 8744570

[ref39] Marvin. 2023, https://chemaxon.com/marvin (accessed Nov 14, 2023).

[ref40] Berman H. M.; et al. The Protein Data Bank. Nucleic Acids Res. 2000, 28 (1), 235–242. 10.1093/nar/28.1.235. 10592235 PMC102472

[ref41] Liu Y.; Liu Y.; Chen H.; Yao X.; Xiao Y.; Zeng X.; Zheng Q.; Wei Y.; Song C.; Zhang Y.; et al. Synthetic resveratrol derivatives and their biological activities: A Review. Open J. Med. Chem. 2015, 05 (04), 97–105. 10.4236/ojmc.2015.54006.

