# Supplementary material for: Five New Tamarixetin Glycosides from Astragalus thracicus Griseb. Including Some Substituted with the Rare 3-Hydroxy-3-methylglutaric Acid and Their Collagenase Inhibitory Effects In Vitro
Source: ACS Omega. 2024 Apr 8;9(16):18023–31. doi: 10.1021/acsomega.3c09677 (PMC11044239; doi:10.1021/acsomega.3c09677)
Supplement: Supplementary file 1 — ao3c09677_si_001.pdf [file ao3c09677_si_001.pdf]

Five New Tamarixetin Glycosides from *Astragalus thracicus* Griseb., Including Some Substituted with the Rare 3-Hydroxy-3-Methylglutaric Acid and Their Collagenase Inhibitory Effects *In Vitro*

Hristo Vasilev,[†,‡] Karel Šmejkal,[‡] Sabina Juskova,[‡] Jiri Vaclavik,[‡*] Jakub Treml[§*]*

[†]Department of Pharmacognosy, Faculty of Pharmacy, Medical University, 2 Dunav str.,1000 Sofia, Bulgaria

[‡]Department of Natural Drugs, Faculty of Pharmacy, Masaryk University, Palackého tř. 1946/1, Brno, 61200, Czech Republic

[§]Department of Molecular Pharmacy, Faculty of Pharmacy, Masaryk University, Palackého tř. 1946/1, Brno, 61200, Czech Republic

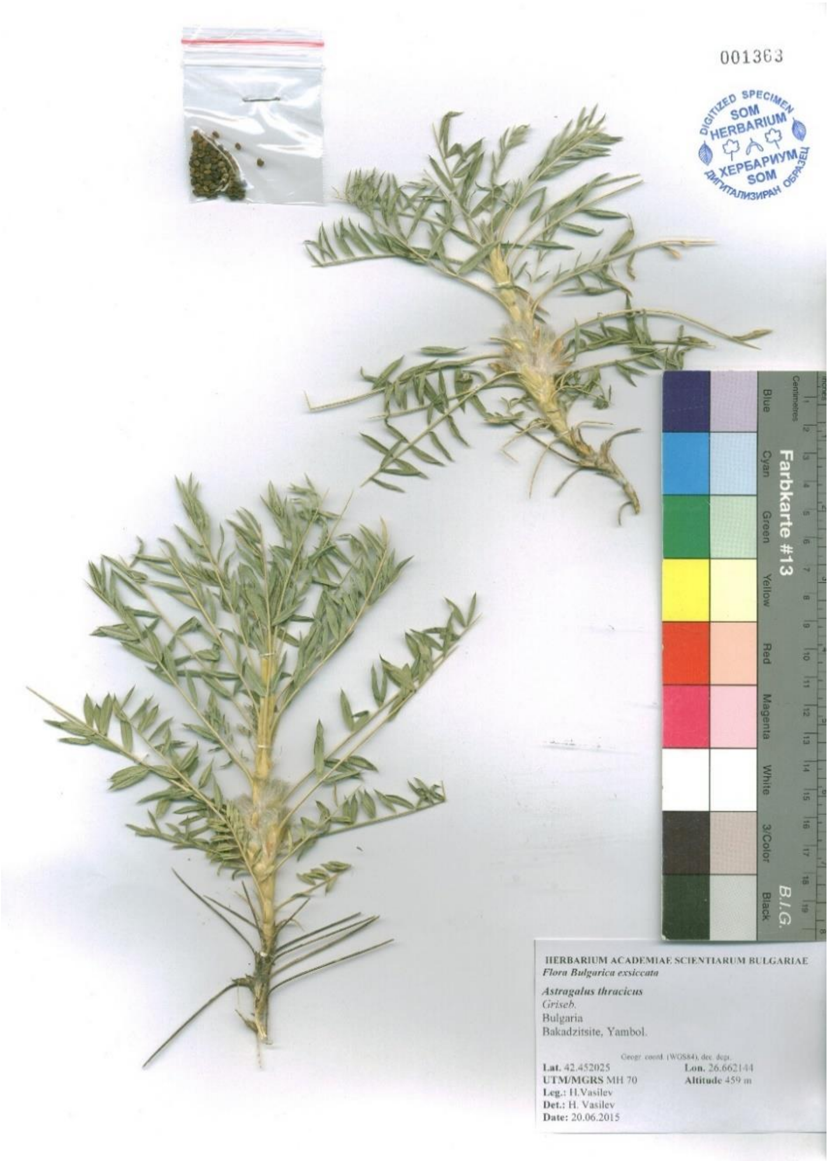

Figure S1: Herbarium specimen of *A. thracicus* Griseb., deposited in the Herbarium of Institute of Biodiversity and Ecosystem Research at the Bulgarian Academy of Sciences (SOM) with Ref № SOM001363

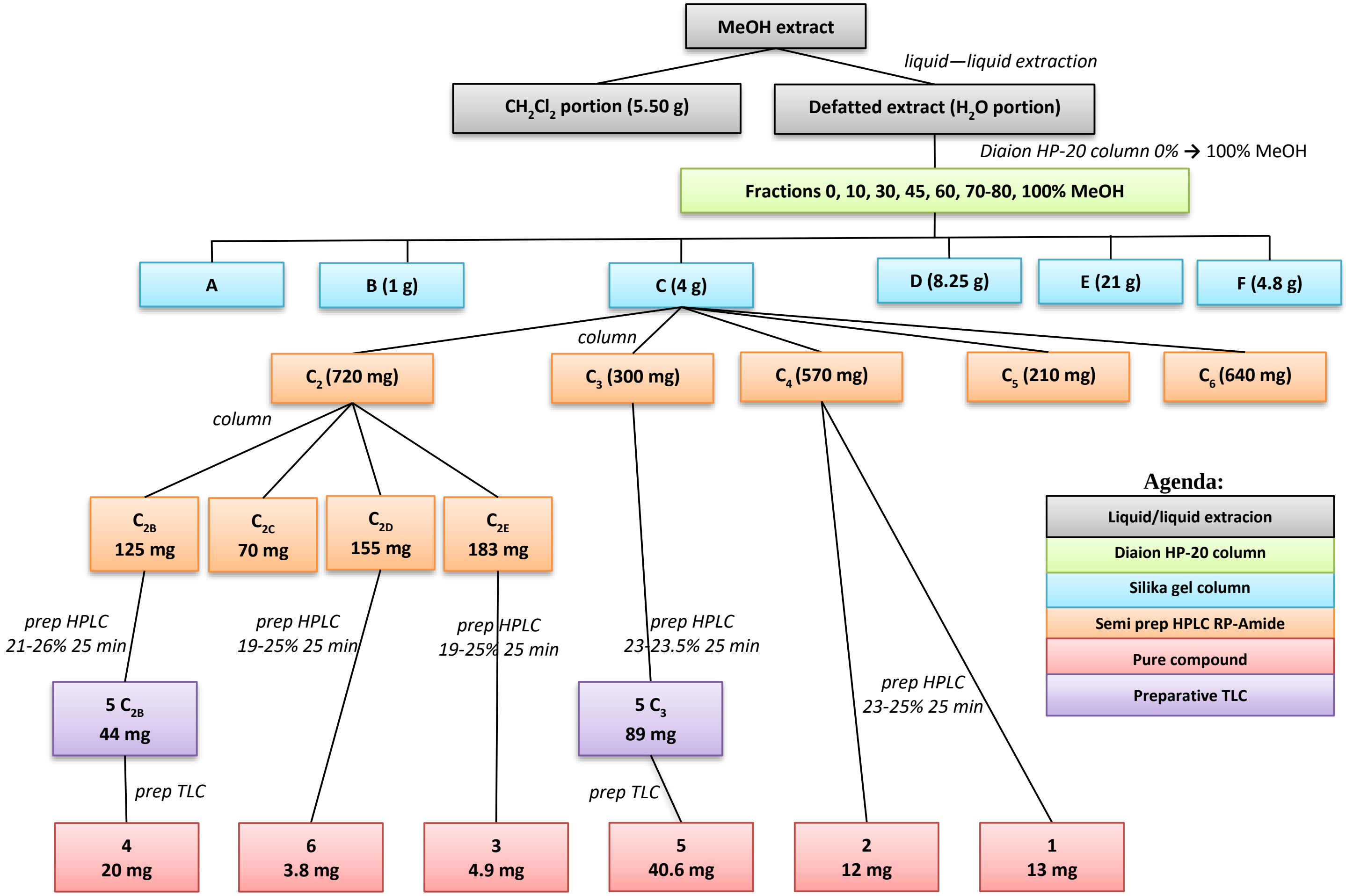

Figure S2: Simplified scheme of separation of the isolated pure compounds from *A. thracicus*

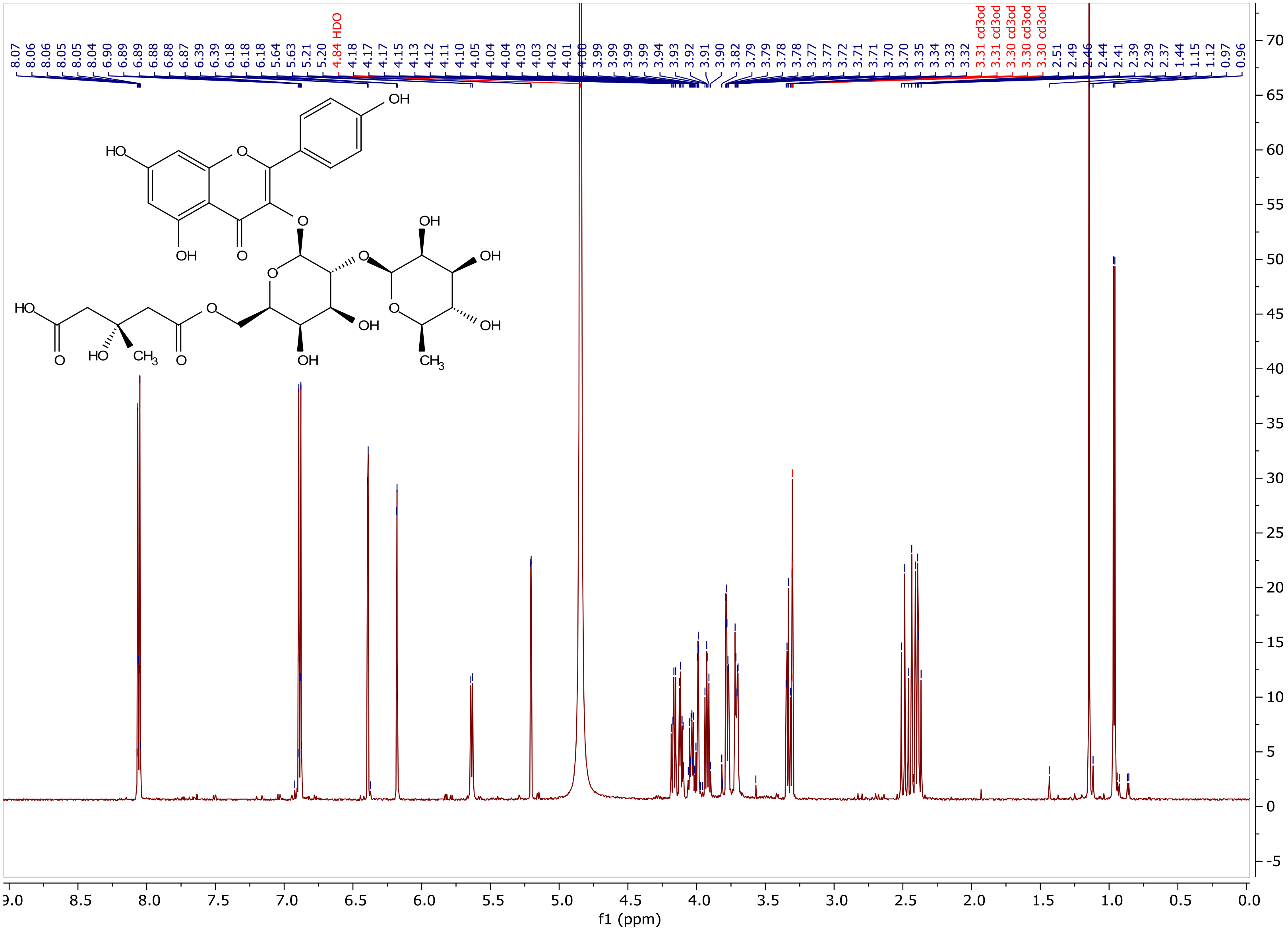

Figure S3: ^{1}H NMR (600 MHz, CD_3OD; 299 °K) spectrum of compound **1**

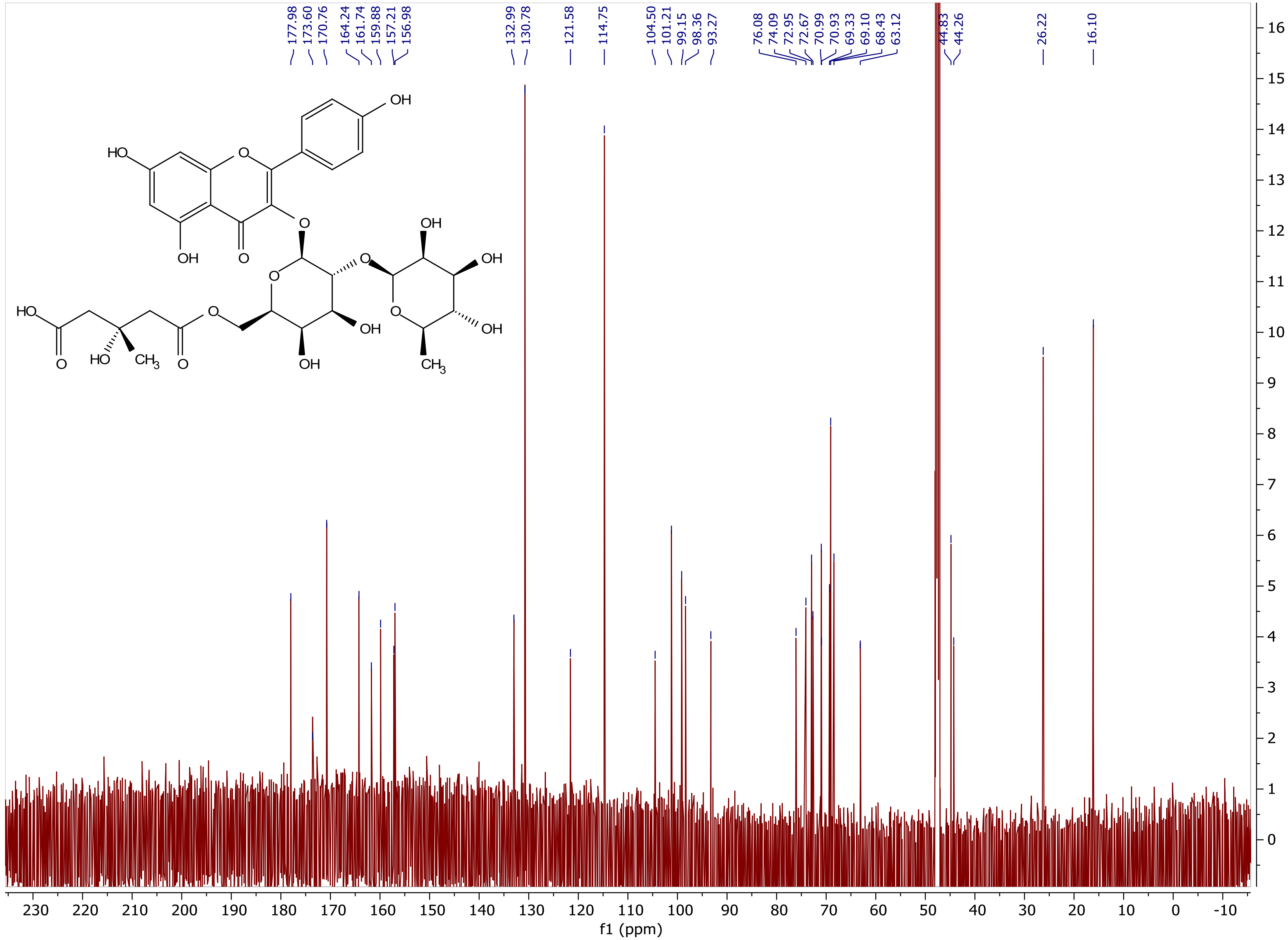

Figure S4: ^{13}C NMR (150 MHz, CD_3OD; 299 °K) spectrum of compound **1**

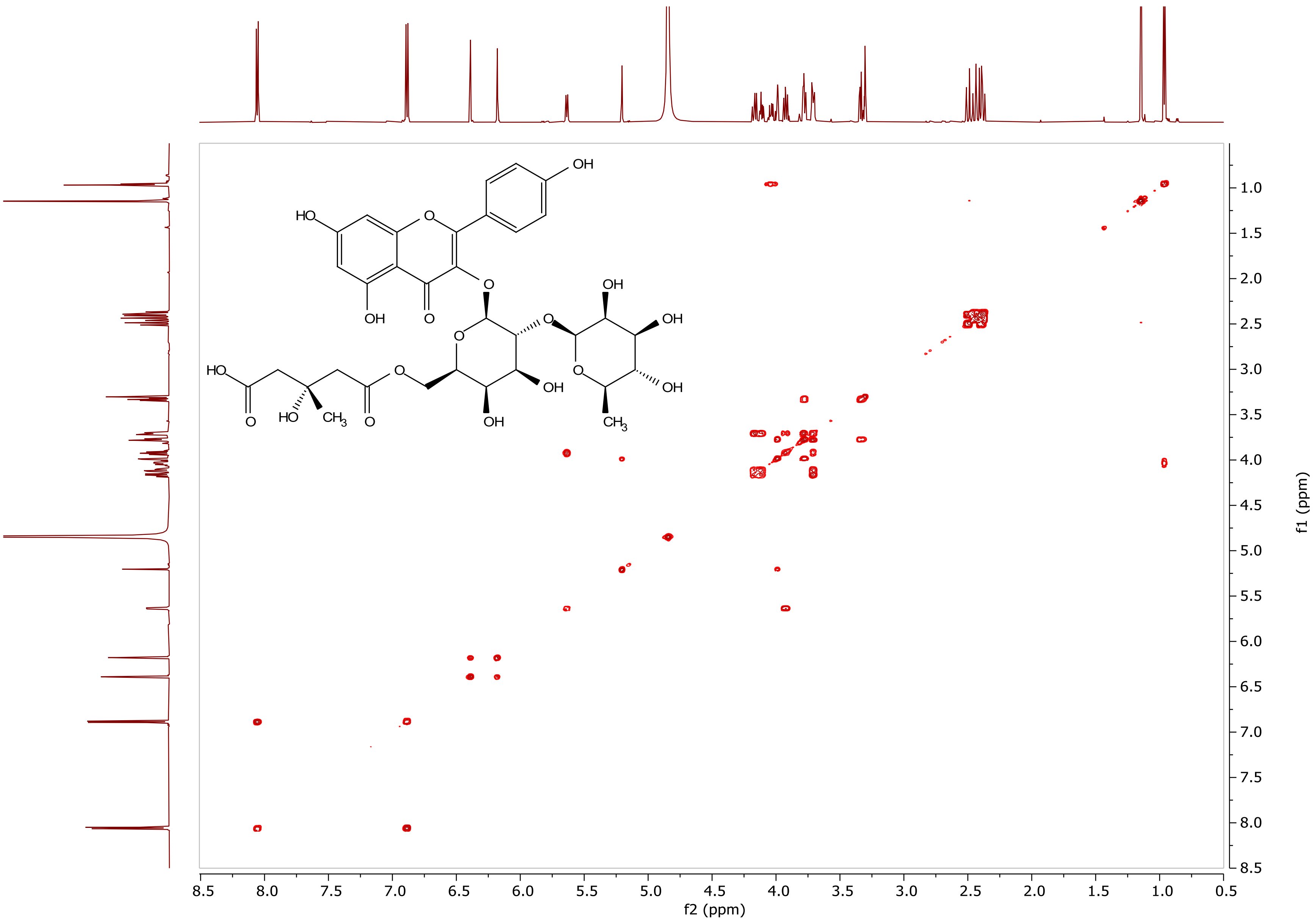

Figure S5: COSY spectrum of compound **1**

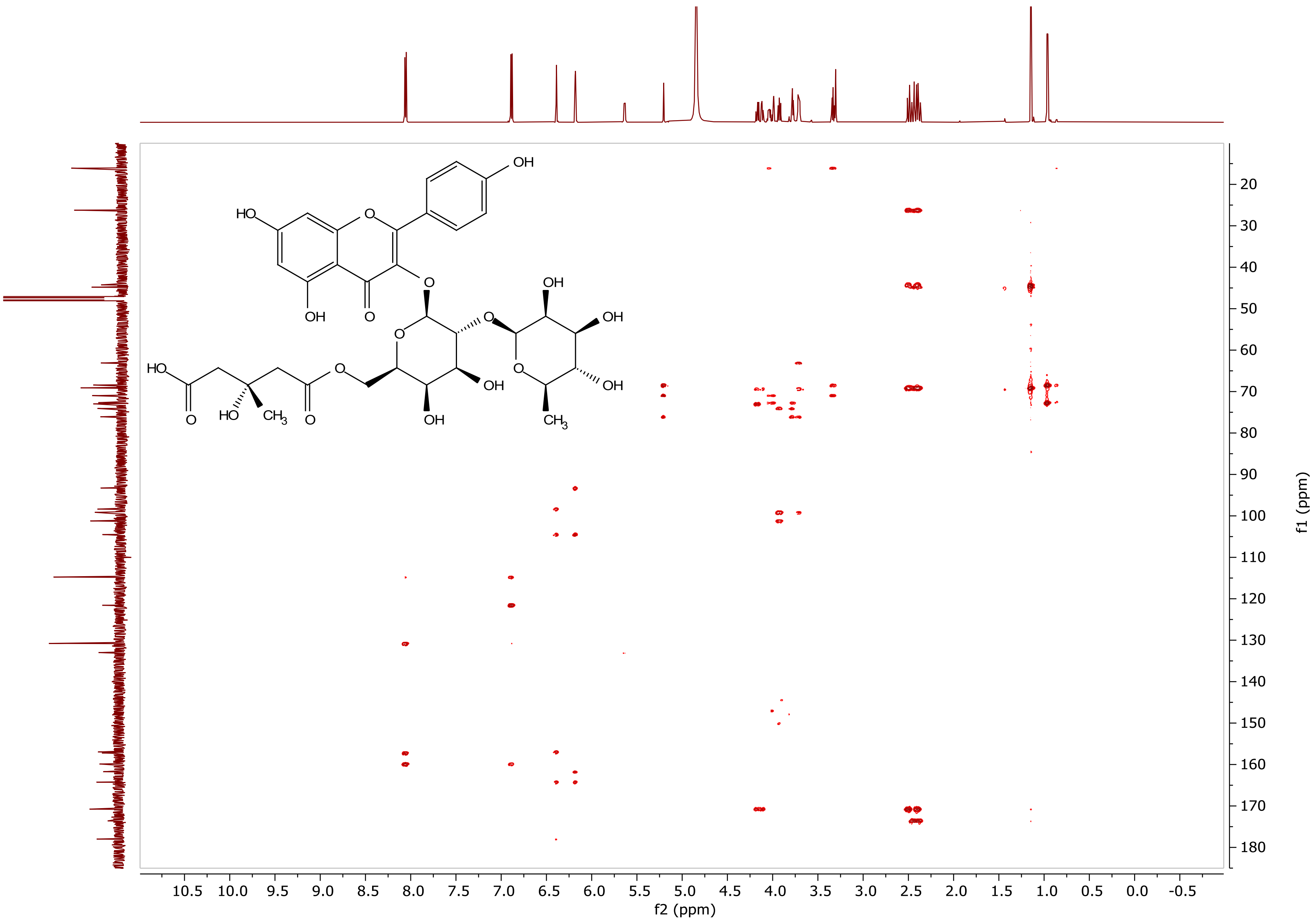

Figure S6: HMBC Spectrum of compound **1**

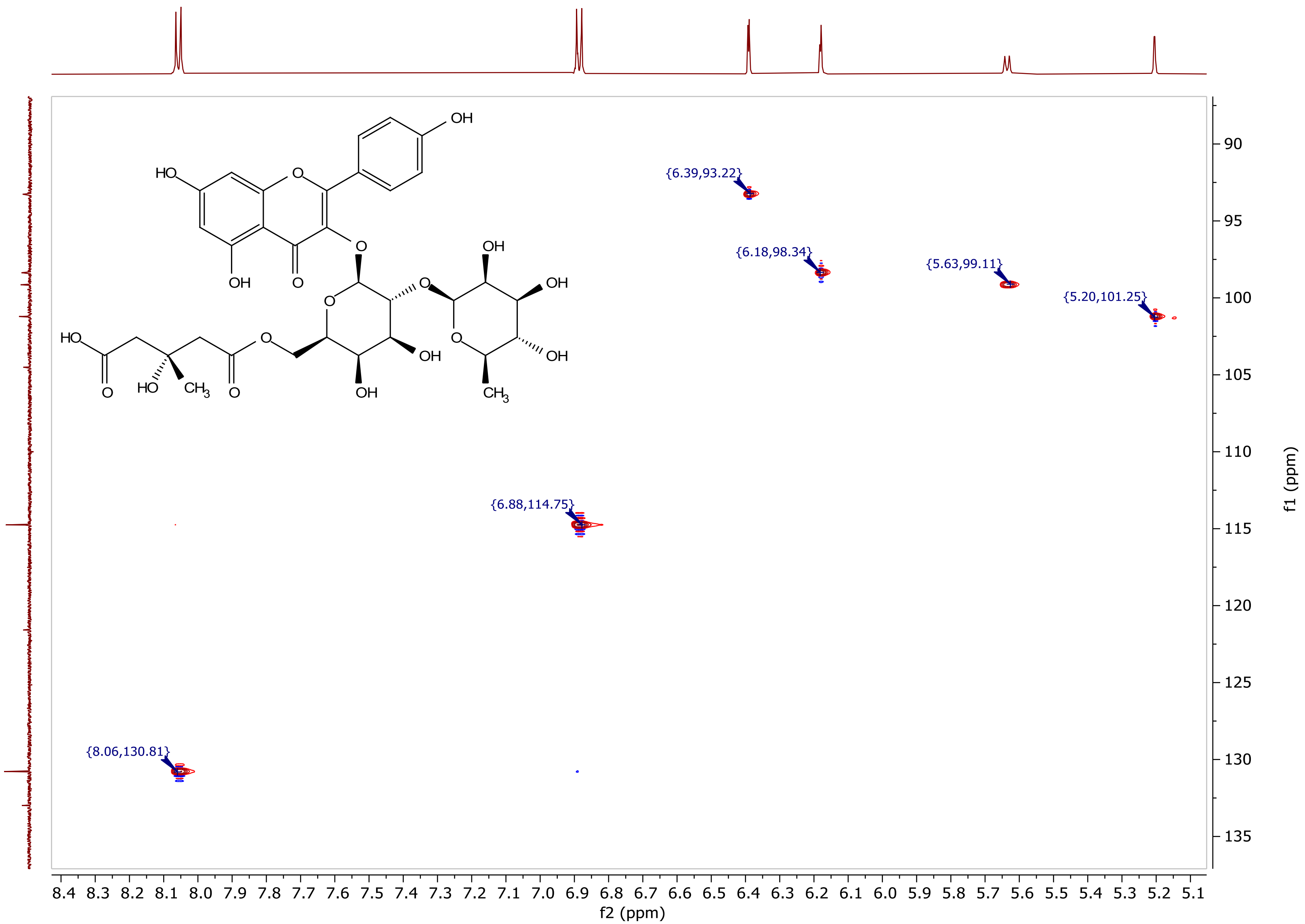

Figure S7: HSQC spectrum of aglycon and anomeric region of compound **1**

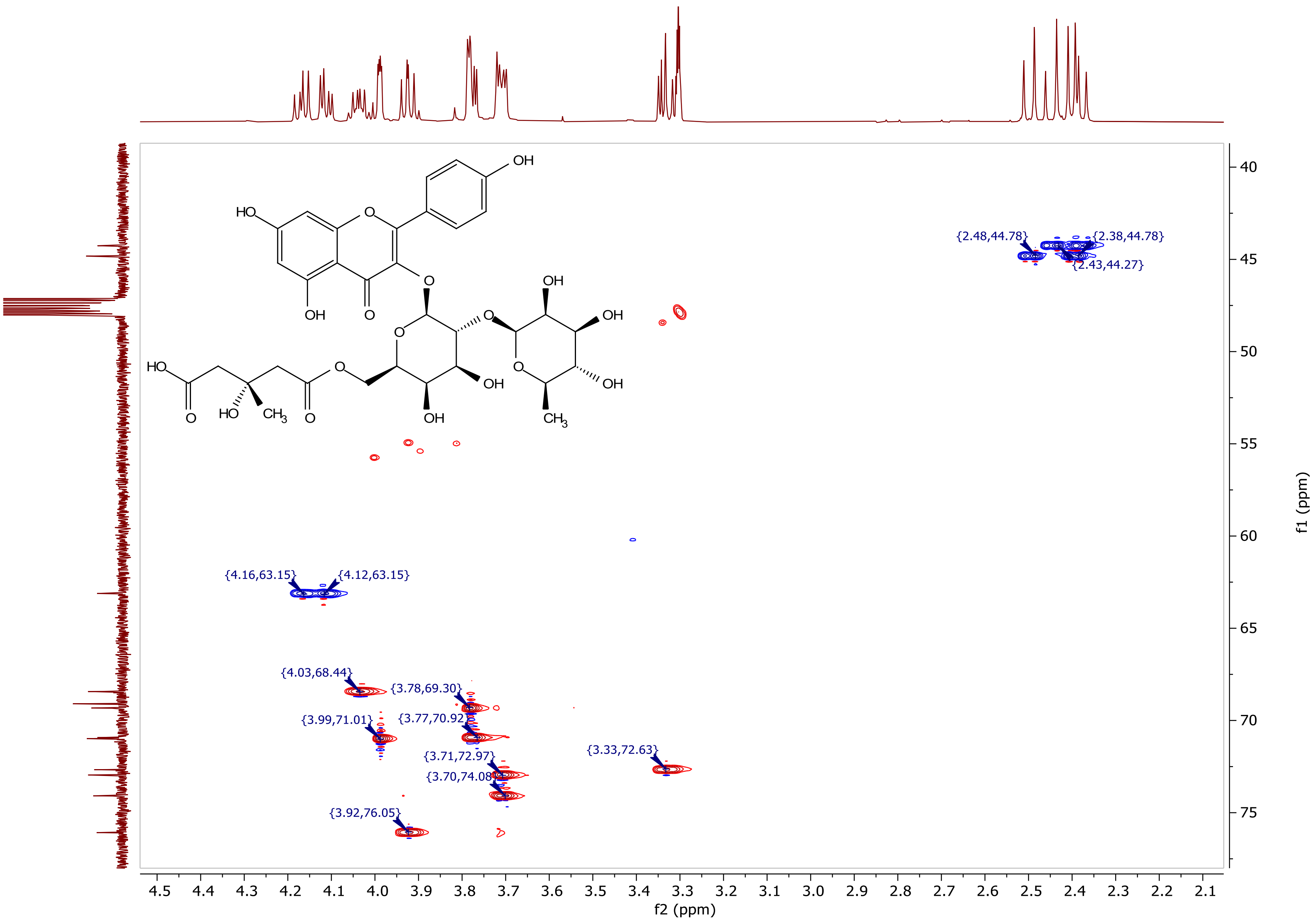

Figure S8: HSQC spectrum of sugars region of compound **1**

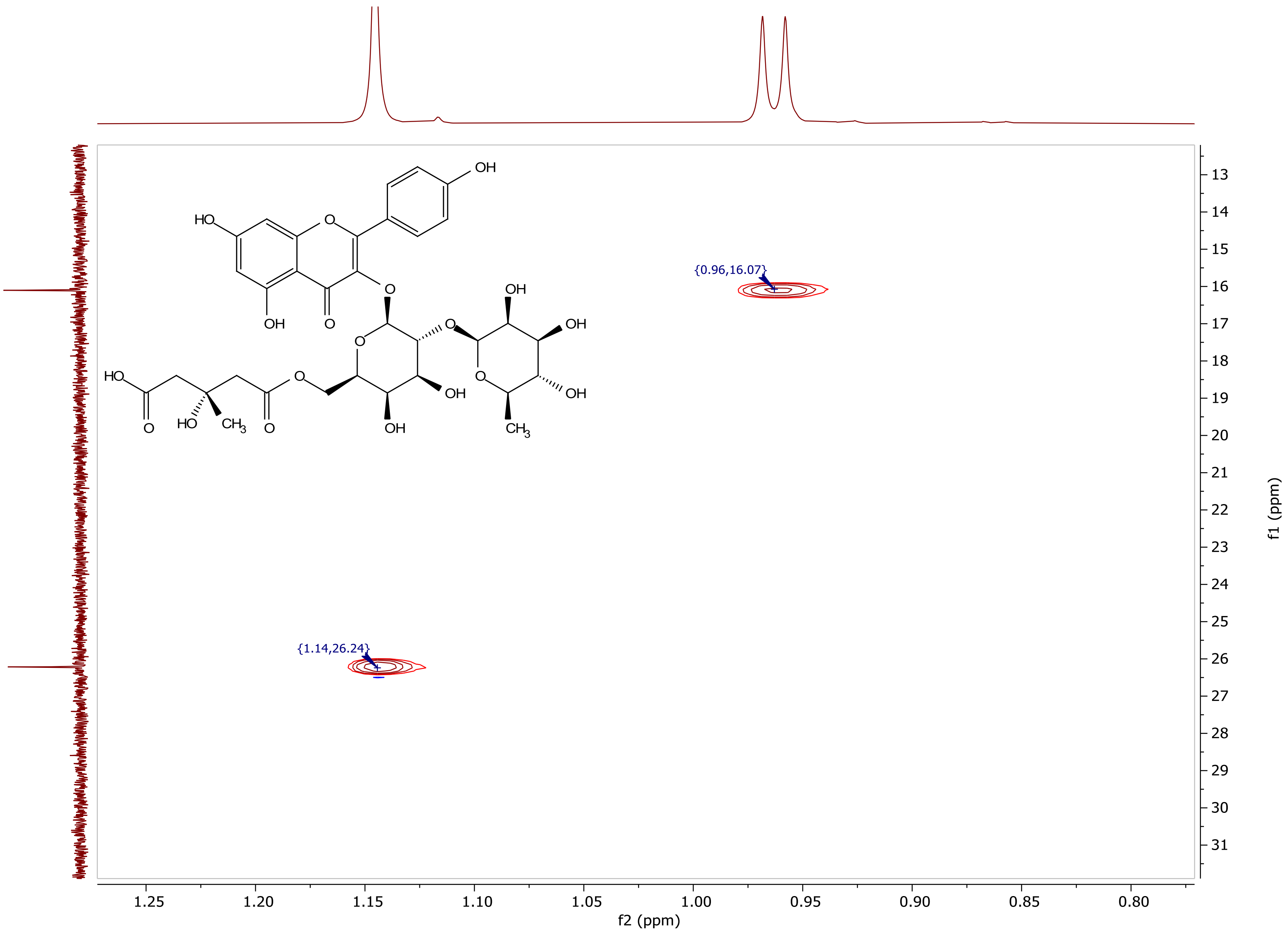

Figure S9: HSQC spectrum of CH_3 at C-4' and CH_3 of rhamnose group found in compound **1**

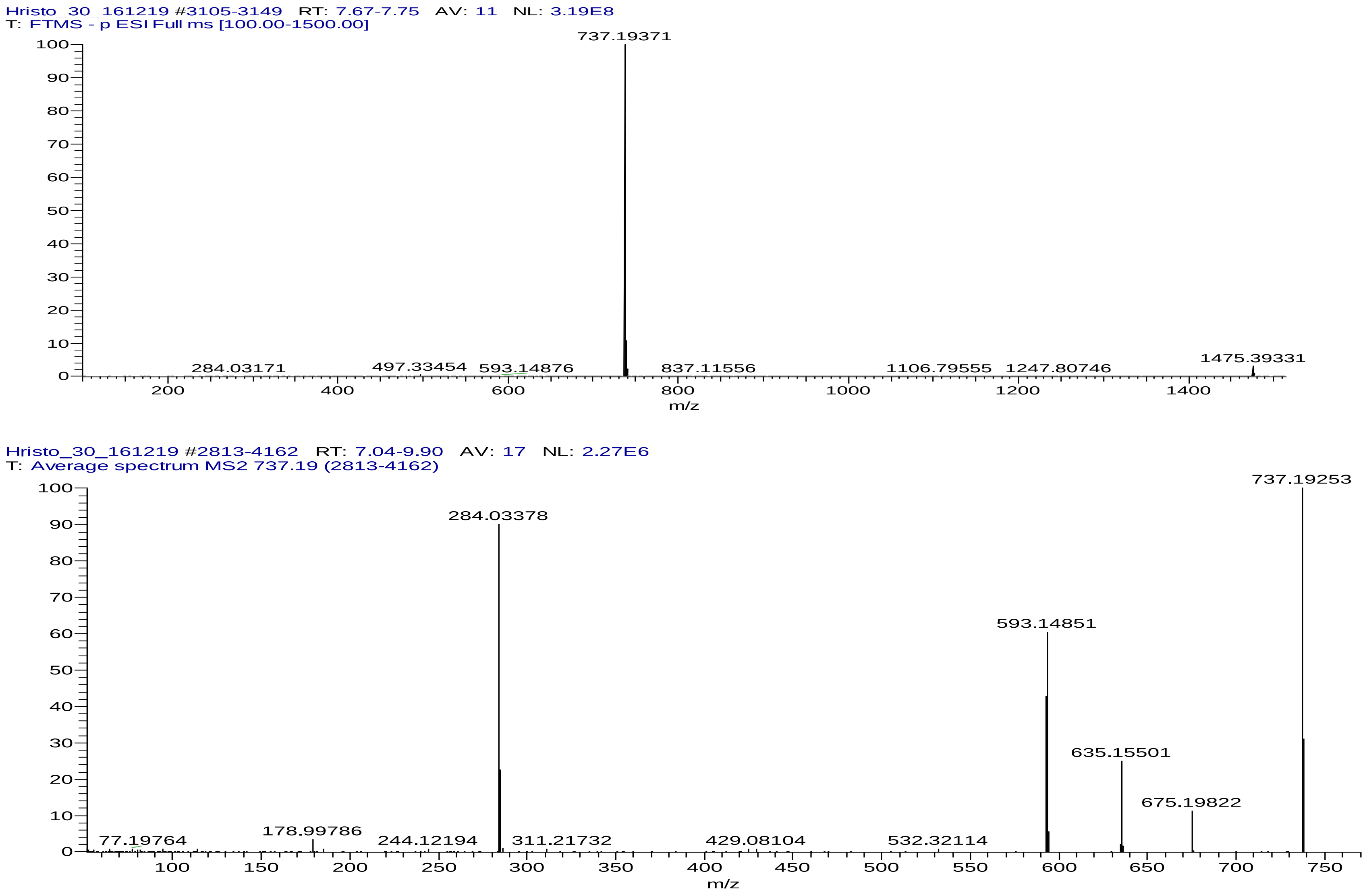

Figure S10: HR-ESI-MS (ESI negative mode) spectrum of compound **1**; up: full-scan, down: MS2 of *m/z* 737.19371.

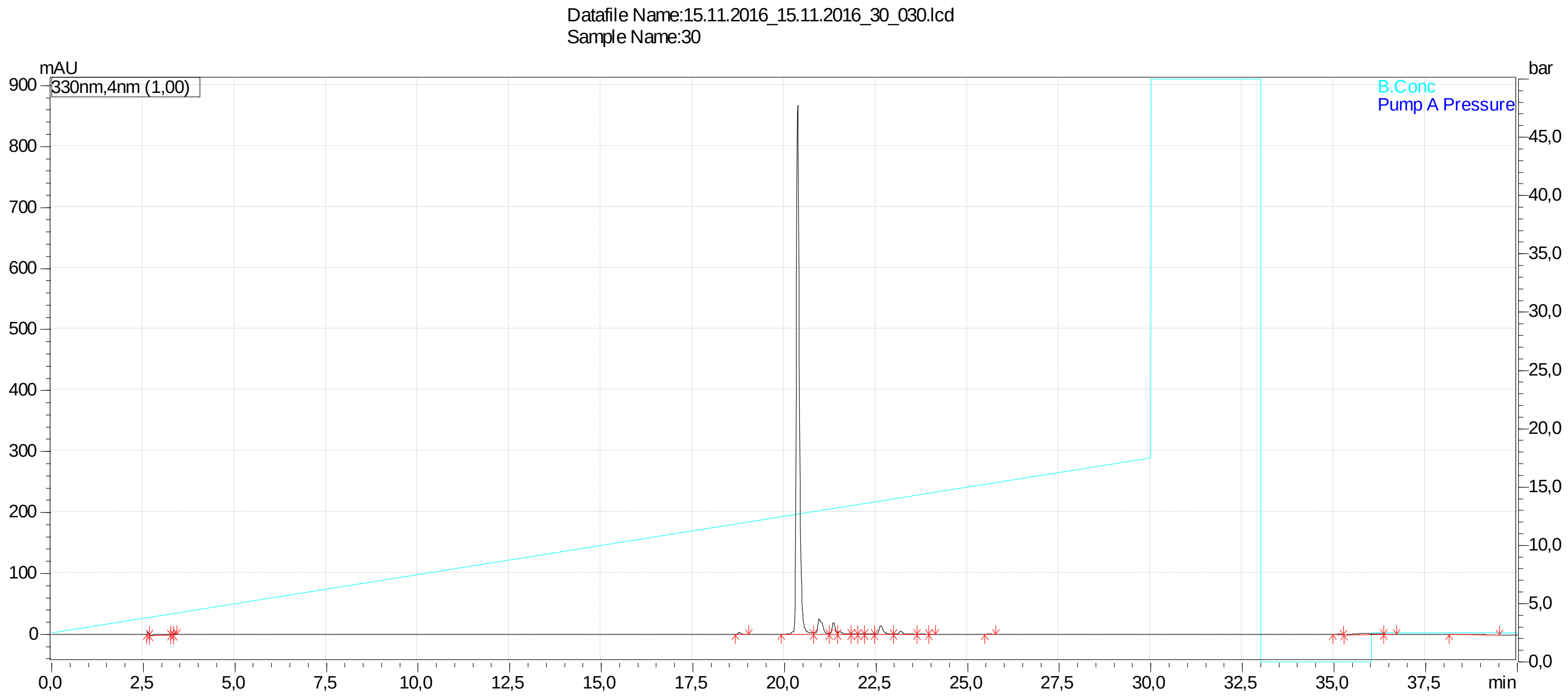

S11: HPLC chromatogram of compound **1**

Gradient 5 → 35%; Solvent A (CH_3CN); Solvent B (H_2O + 0.02% HCOOH);

Column: Kinetex® PFP 100 A, 250 x 4.6 mm I.D., 5 µm (Phenomenex, USA); Flow: 1 ml/min

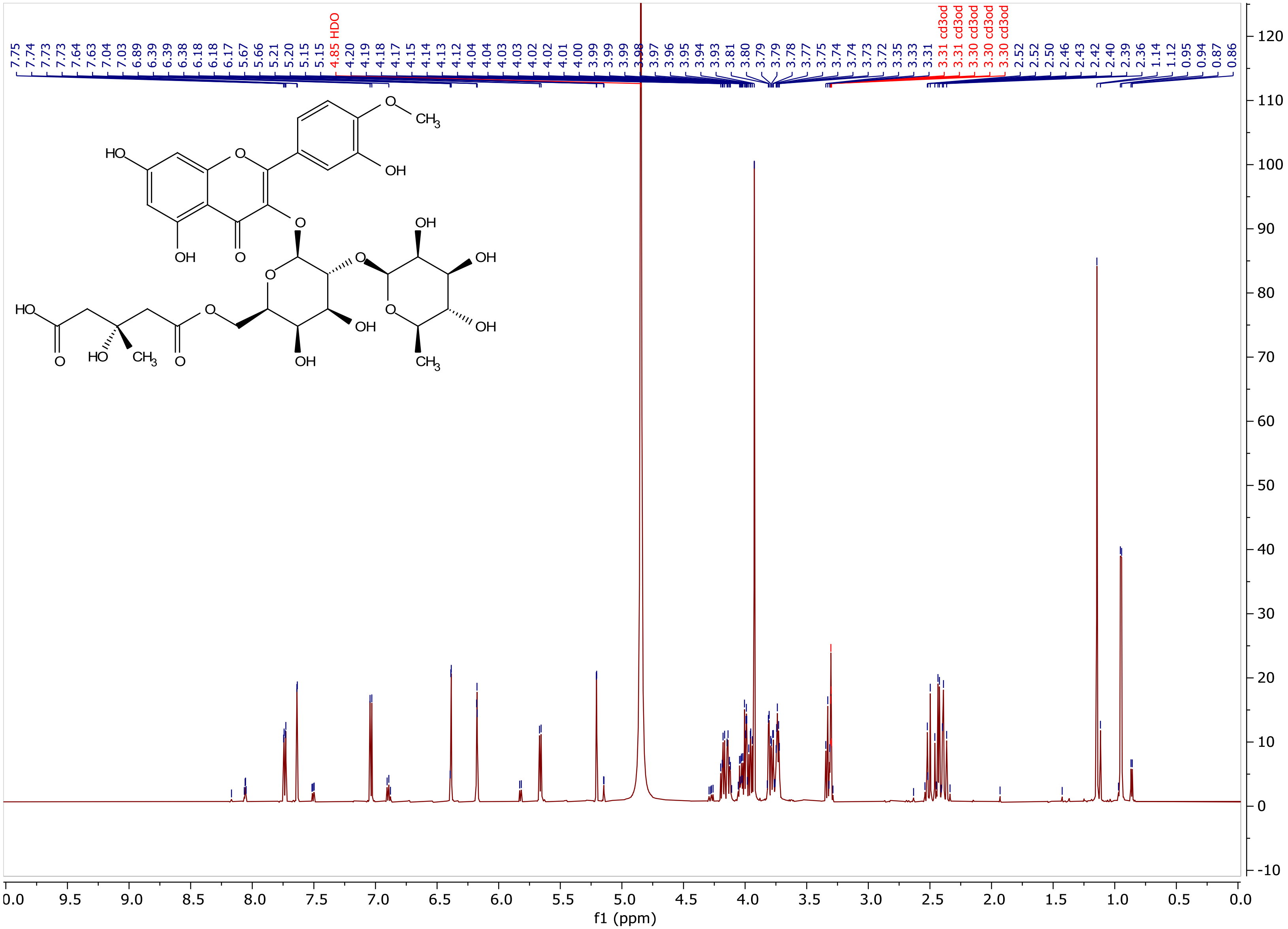

Figure S12: ^{1}H NMR (600 MHz, CD_3OD; 299 °K) spectrum of compound **2**

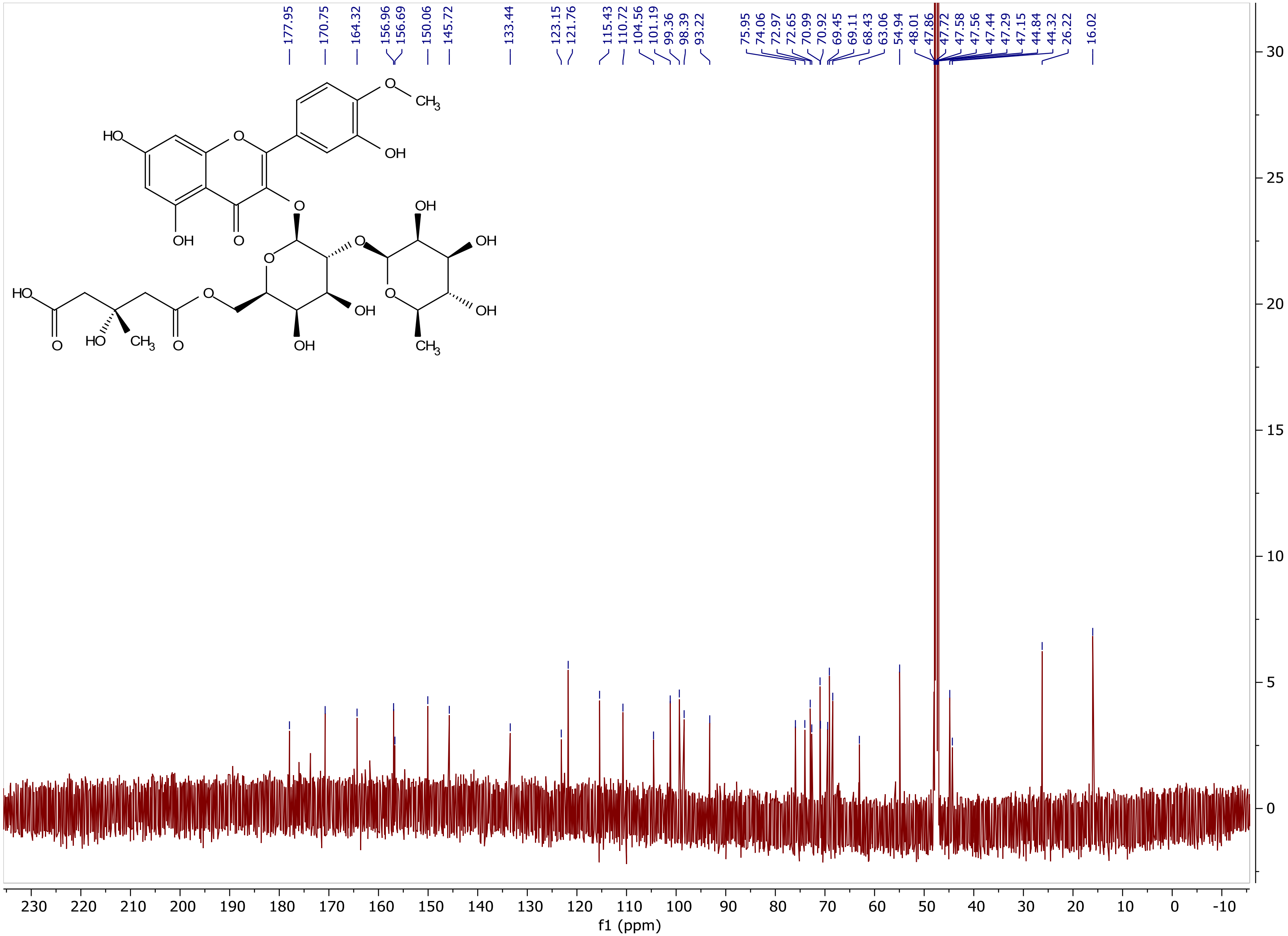

Figure S13: ^{13}C NMR (150 MHz, CD_3OD; 299 °K) spectrum of compound **2**

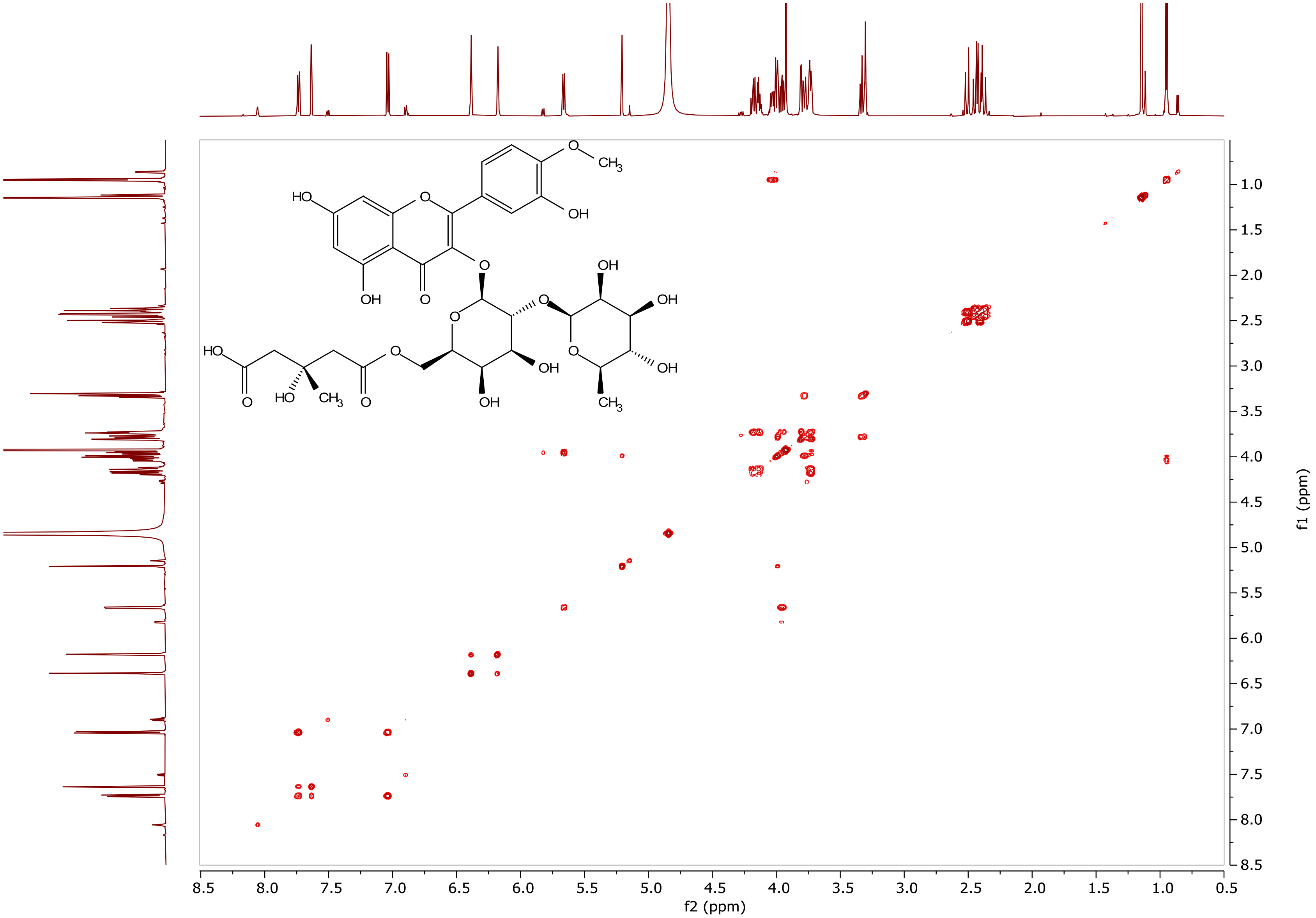

Figure S14: COSY spectrum of compound **2**

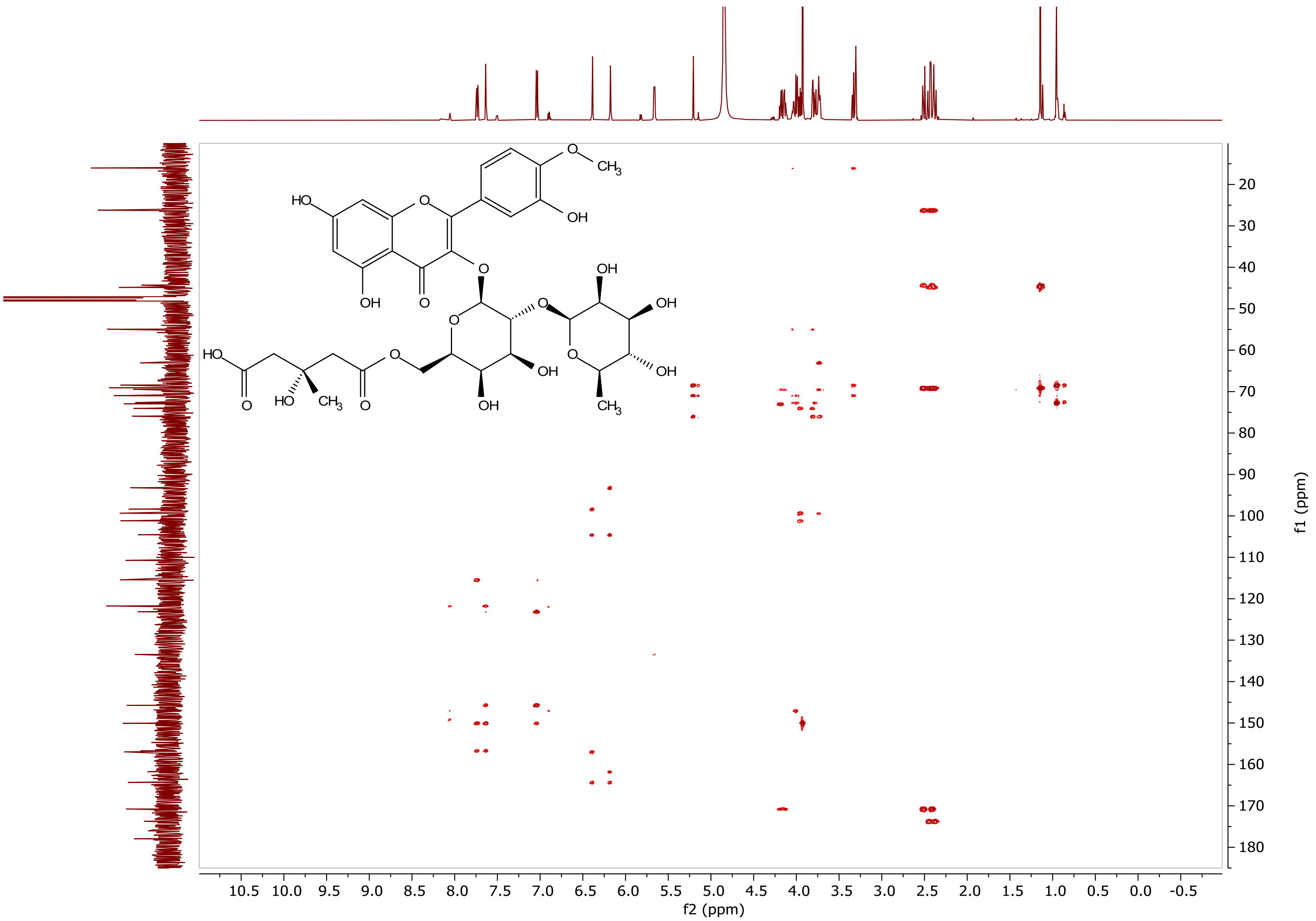

Figure S15: HMBC spectrum of compound **2**

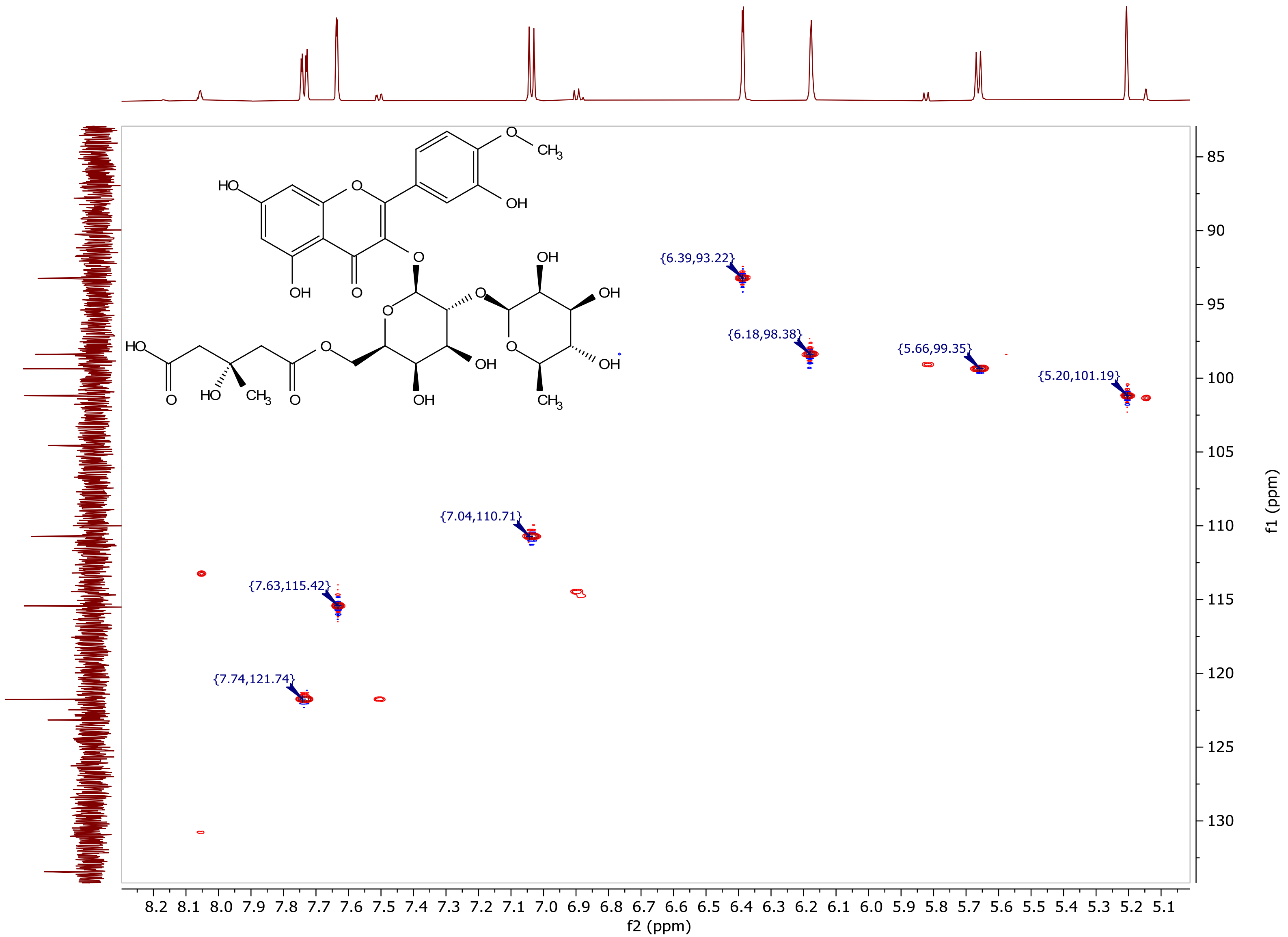

Figure S16: HSQC spectrum of aglycon and anomeric region of compound **2**

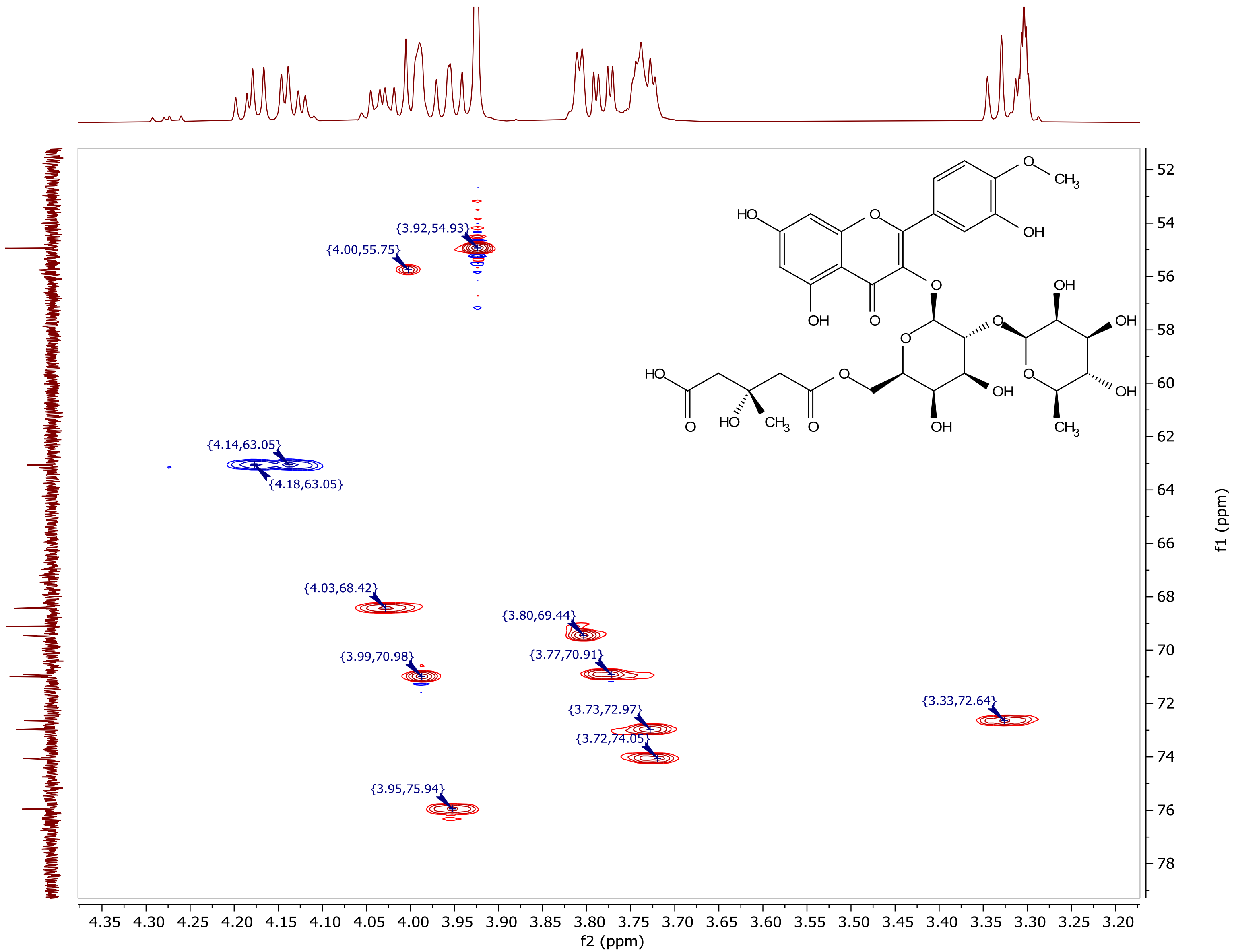

Figure S17: HSQC spectrum of sugars region of compound **2**

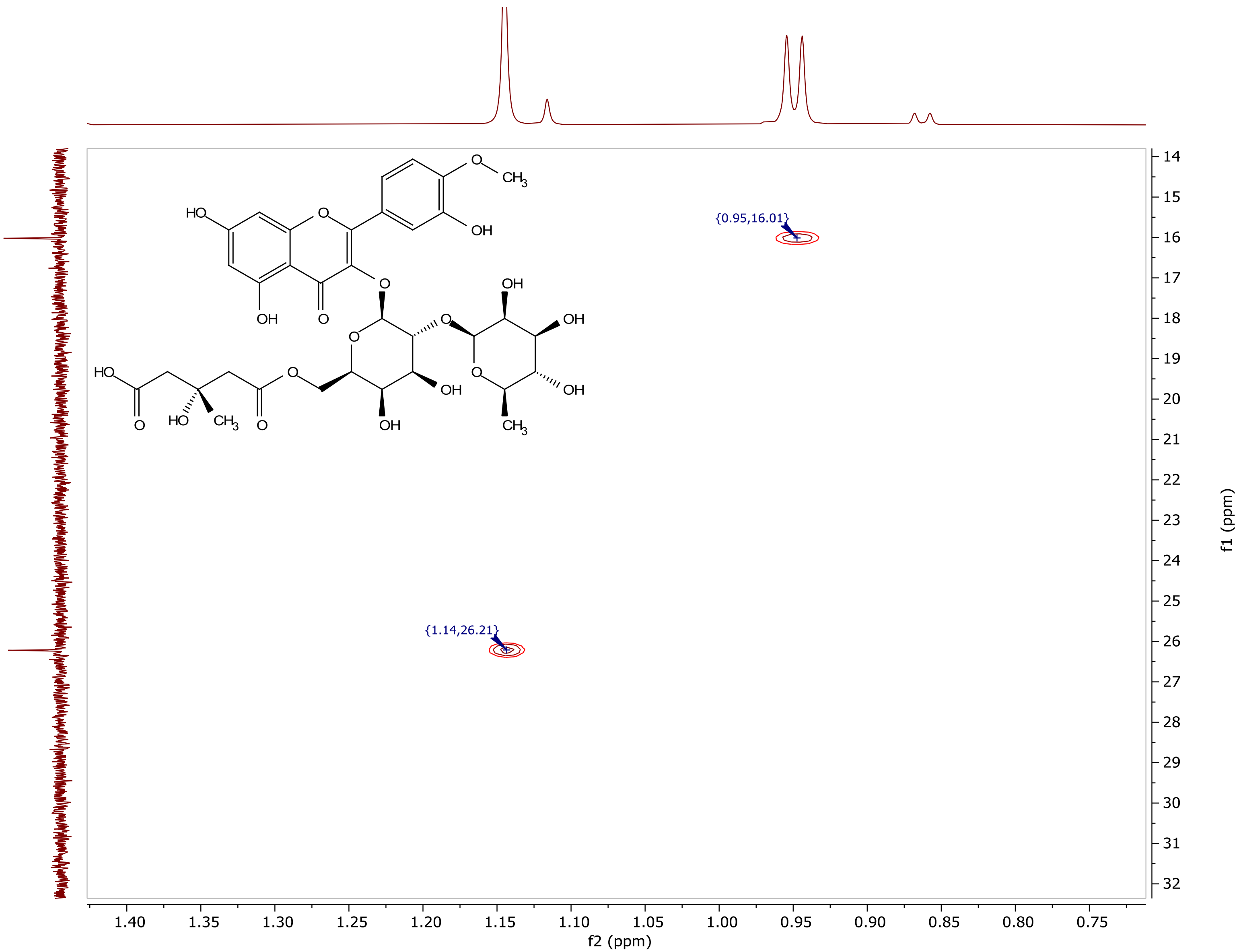

Figure S18: HSQC spectrum of CH_3 at C-4' and CH_3 of rhamnose group found in compound **2**

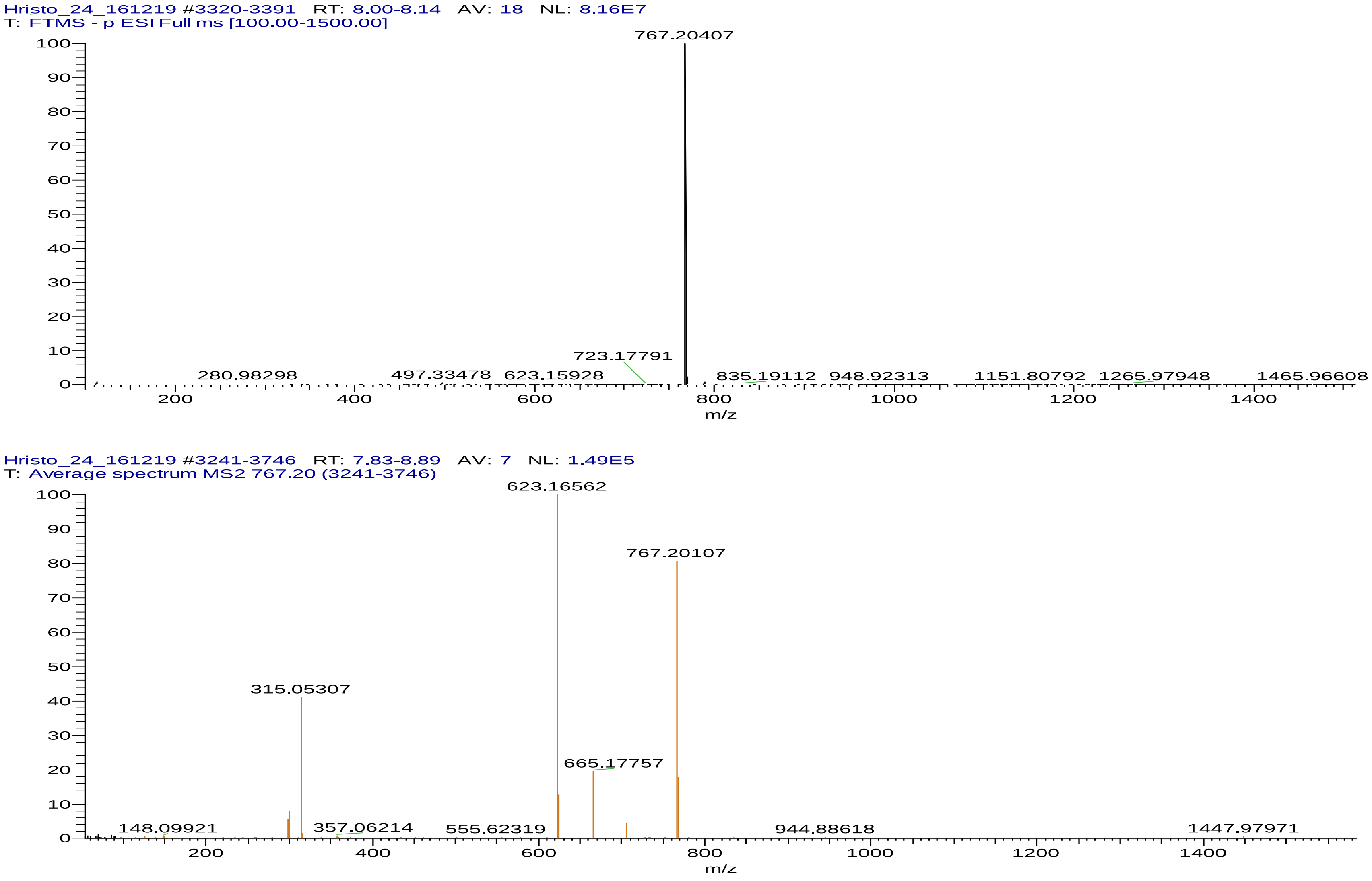

Figure S19: HR-ESI-MS (ESI negative mode) spectrum of compound **2**

Up: full-scan

Down: MS2 of 767.20407 m/z

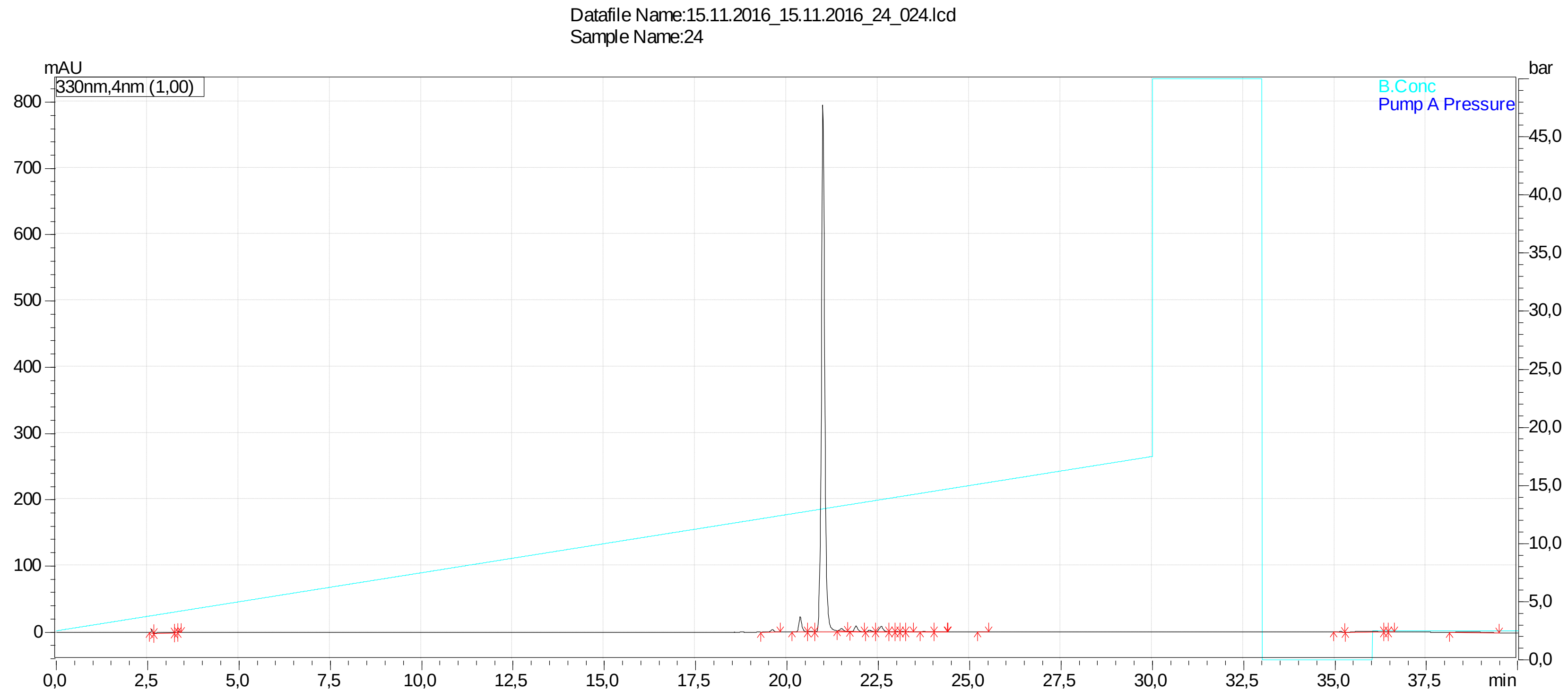

Figure S20: HPLC chromatogram of compound **2**

Gradient 5 → 35%; Solvent A (CH_3CN); Solvent B (H_2O + 0.02% HCOOH);

Column: Kinetex® PFP 100 A, 250 x 4.6 mm I.D., 5 µm (Phenomenex, USA); Flow: 1 ml/min

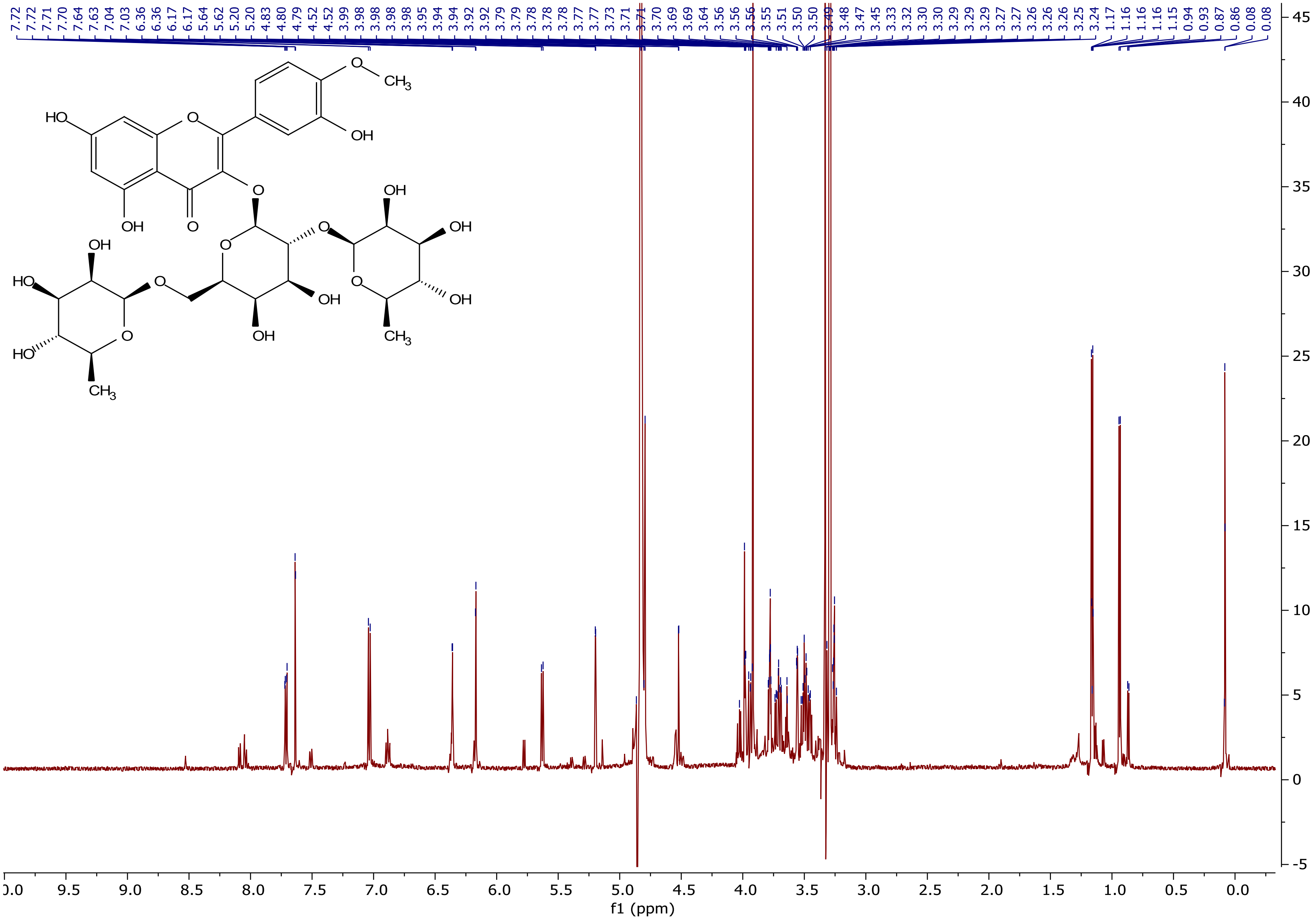

Figure S21: ^{1}H NMR (600 MHz, CD_3OD; 299 °K) spectrum of compound **3**

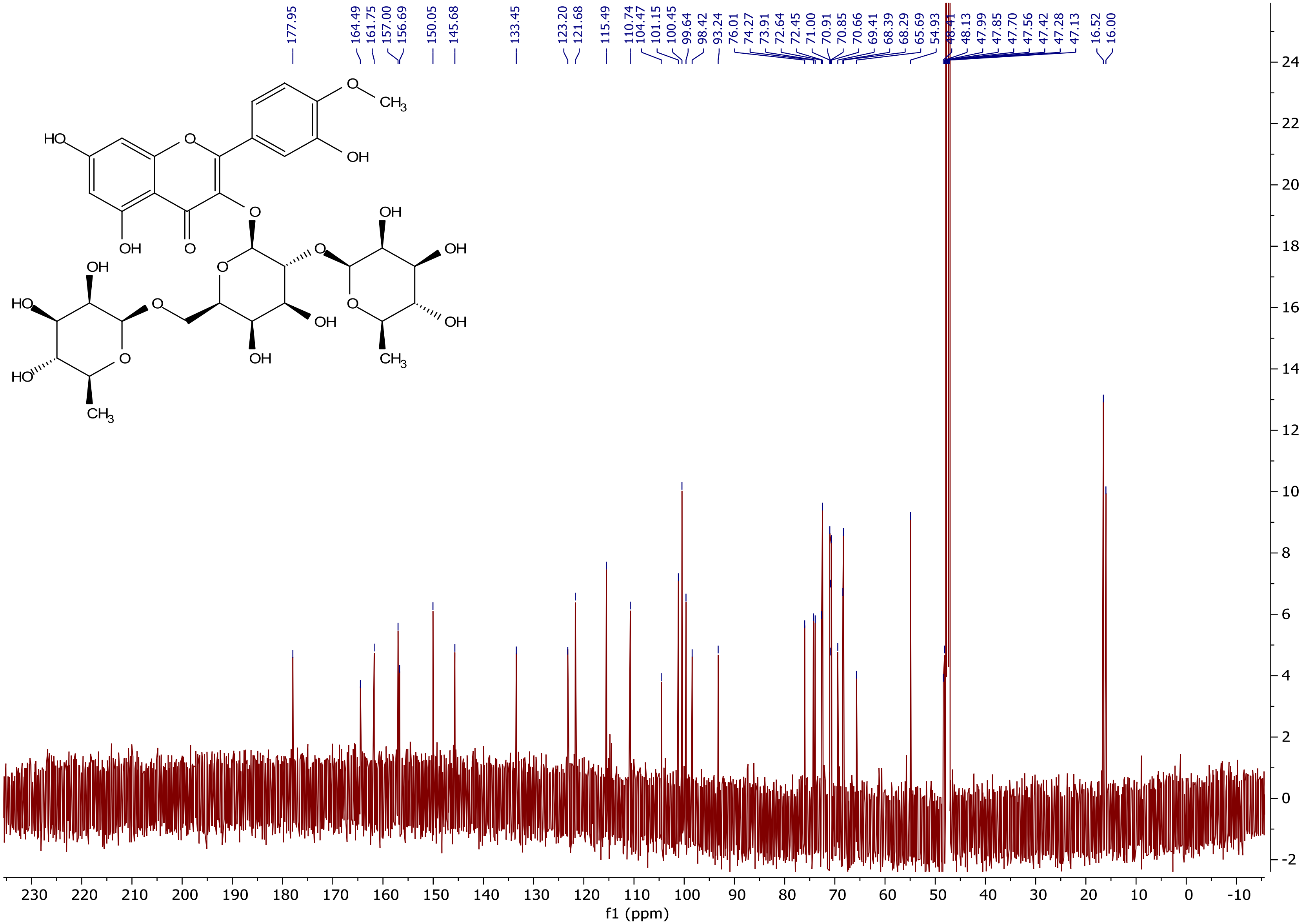

Figure S22: ^{13}C NMR (150 MHz, CD_3OD; 299 °K) spectrum of compound **3**

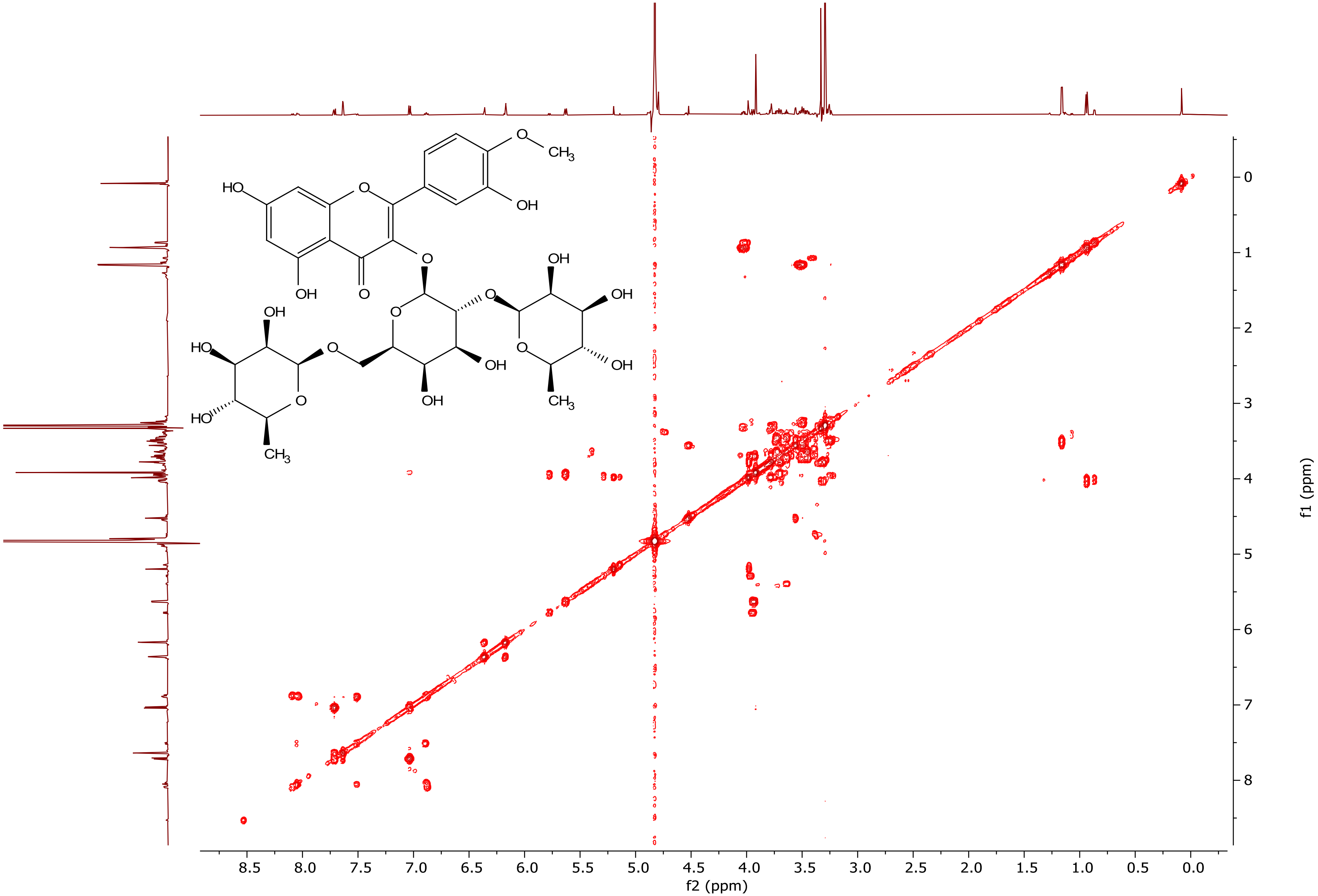

Figure S23: COSY spectrum of compound **3**

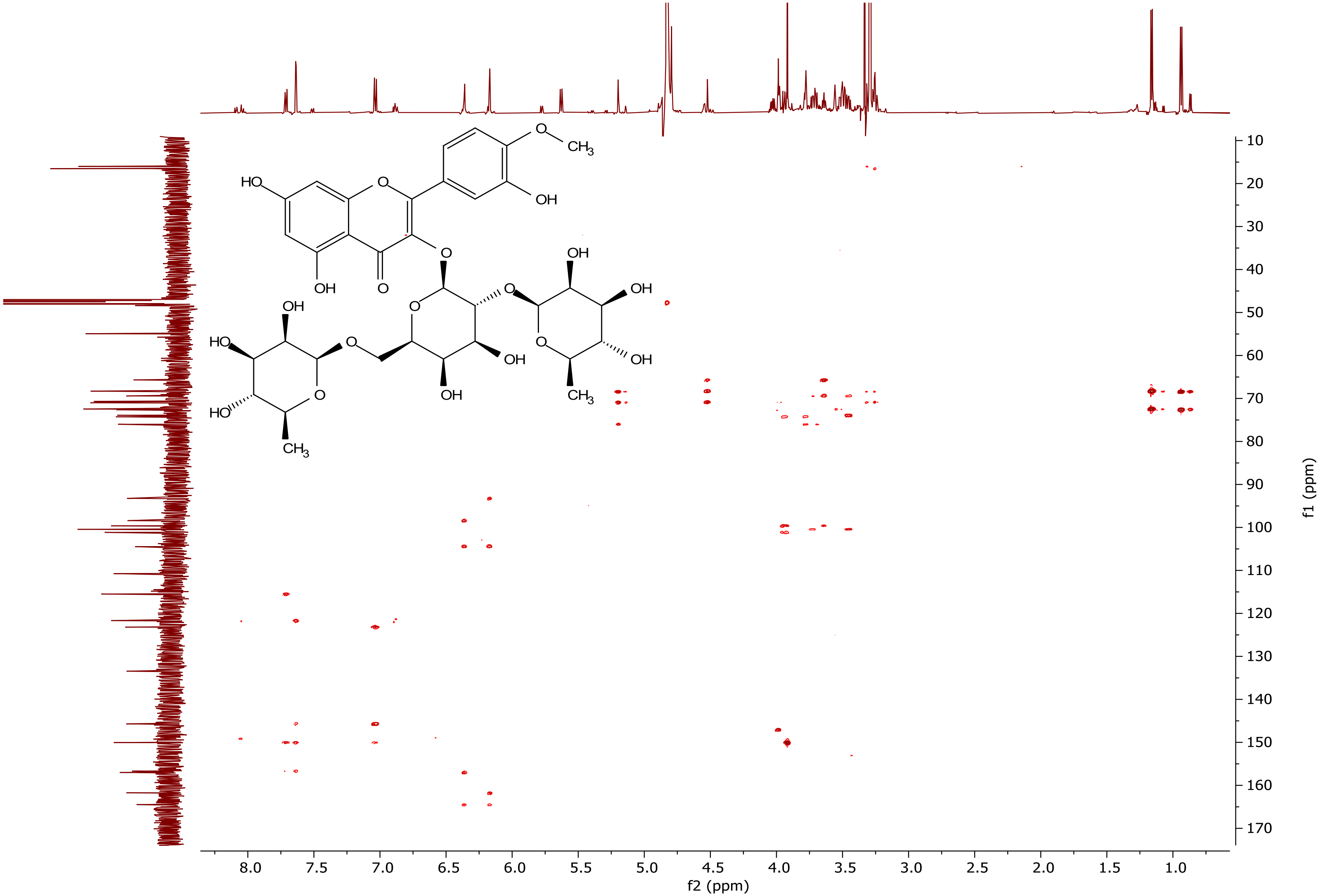

Figure S24: HMBC Spectrum of compound **3**

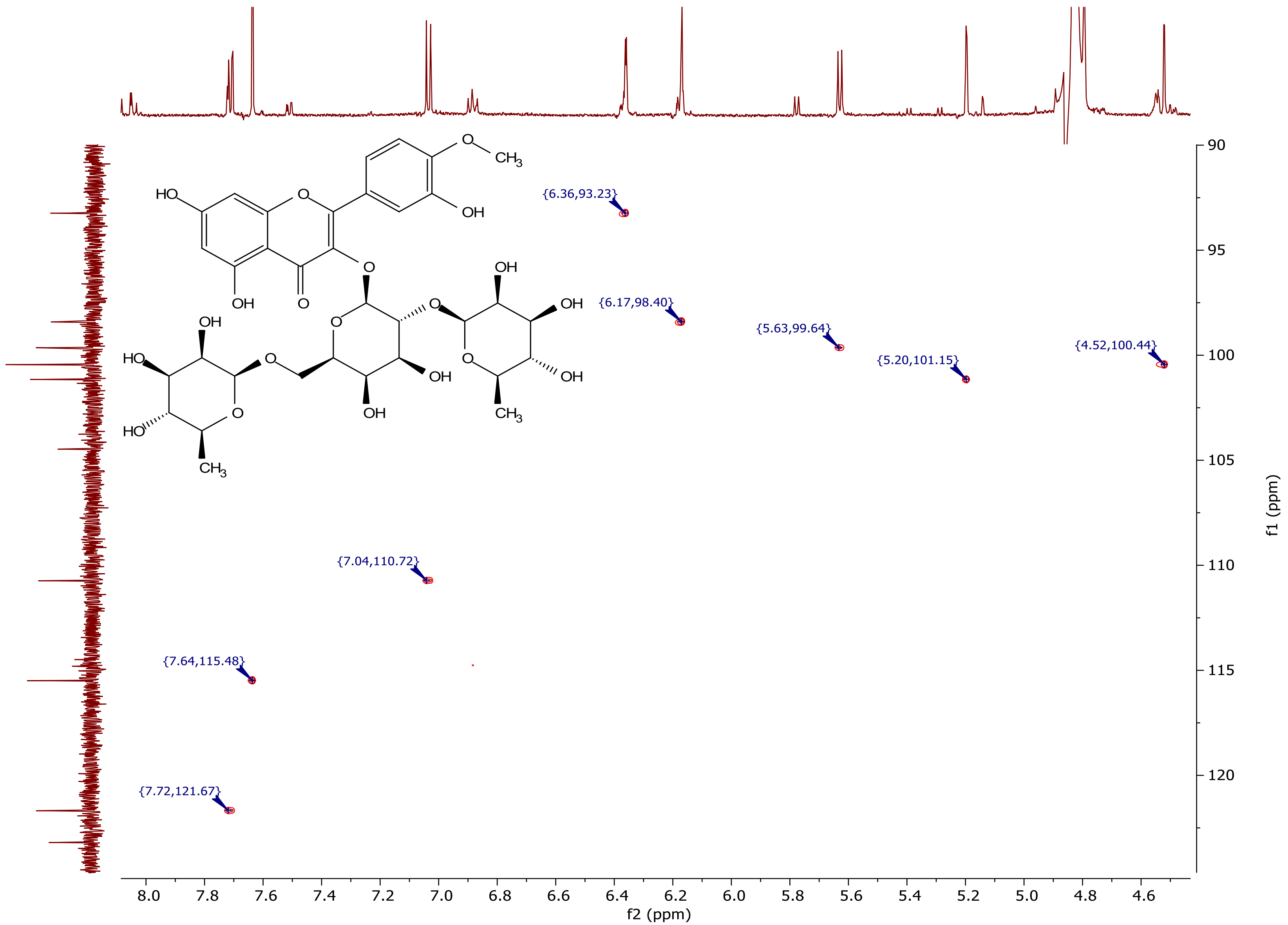

Figure S25: HSQC spectrum of aglycon and anomeric region of compound **3**

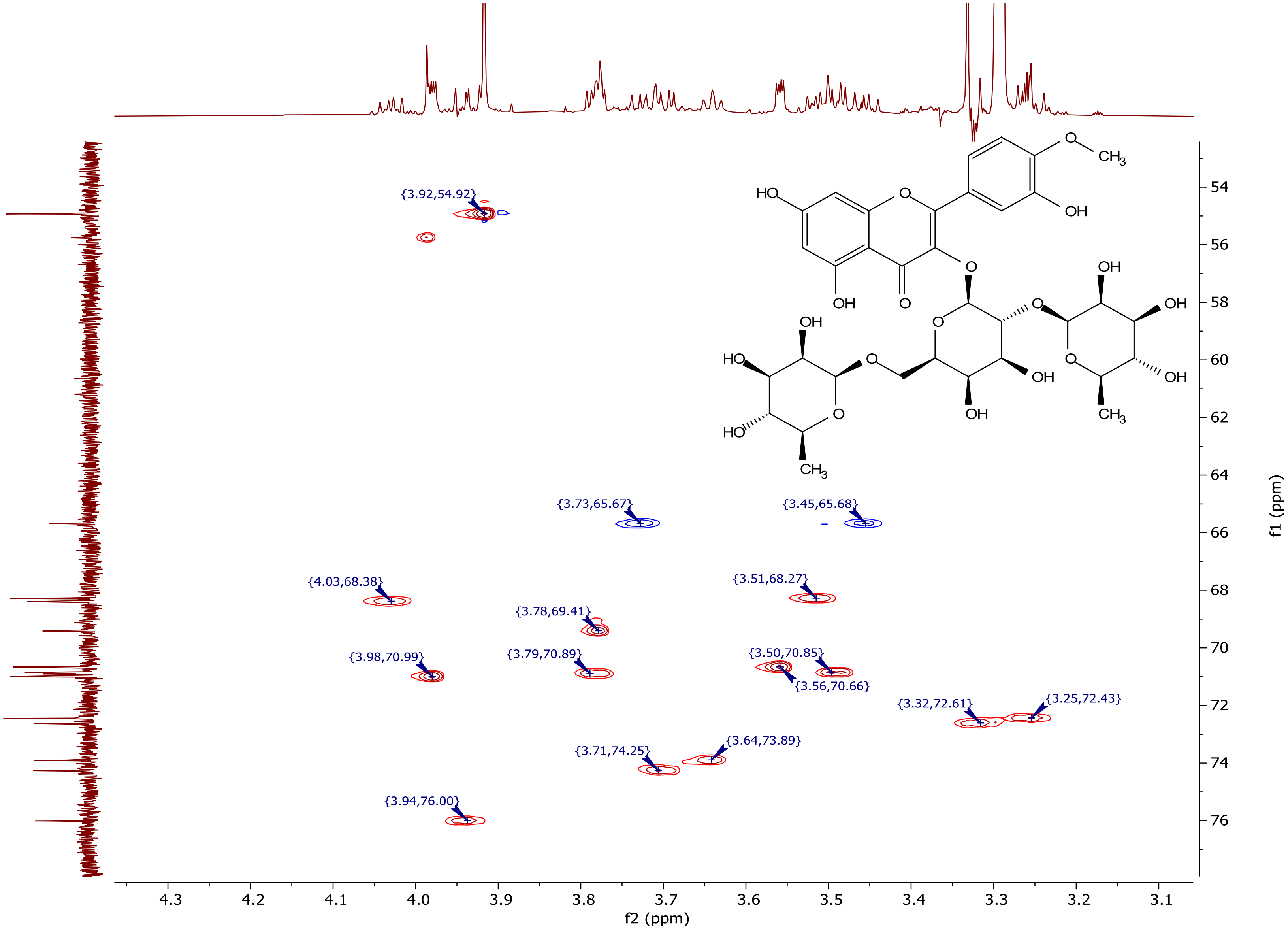

Figure S26: HSQC spectrum of sugars region of compound **3**

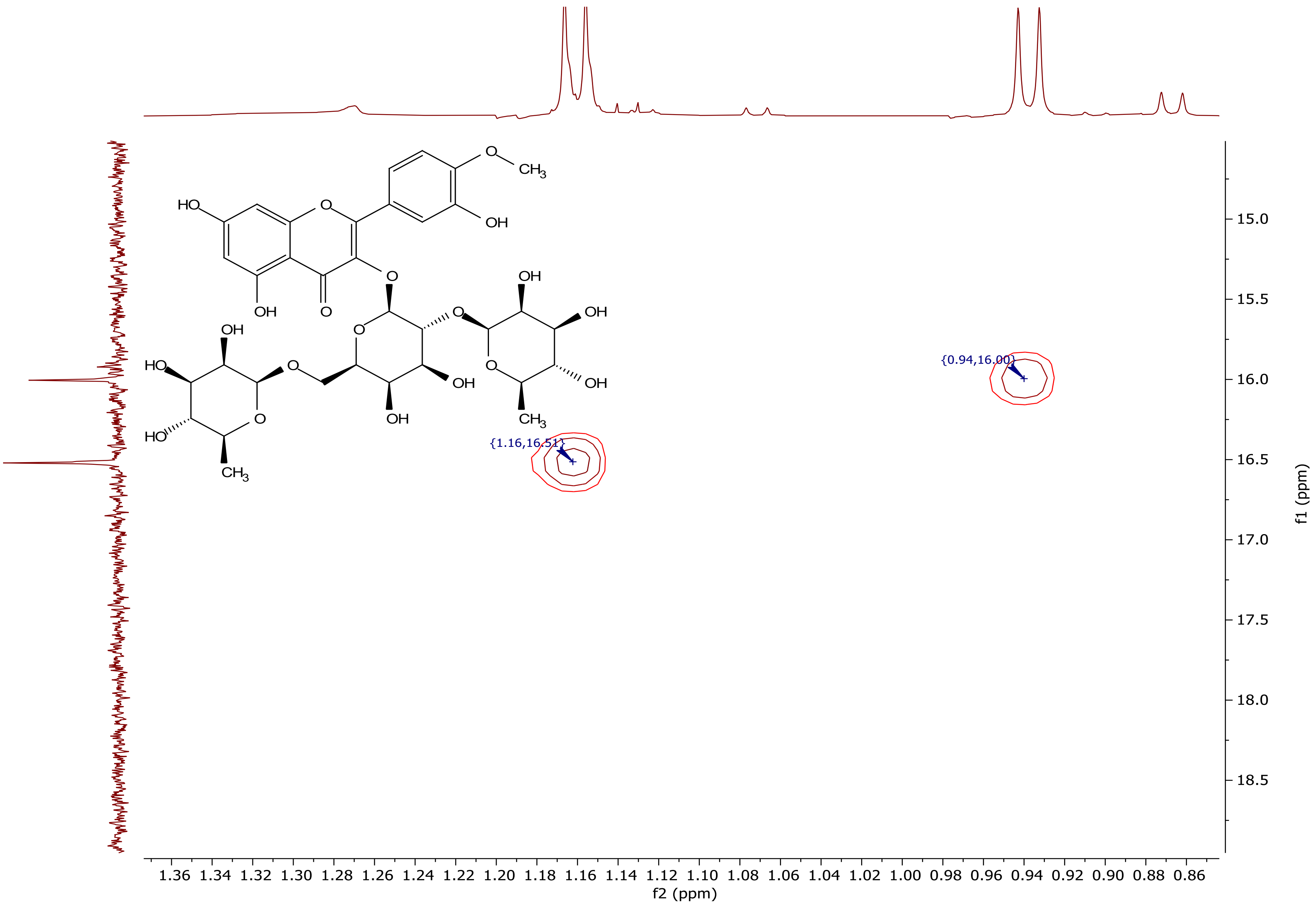

Figure S27: HSQC spectrum CH_3 of rhamnose groups of compound **3**

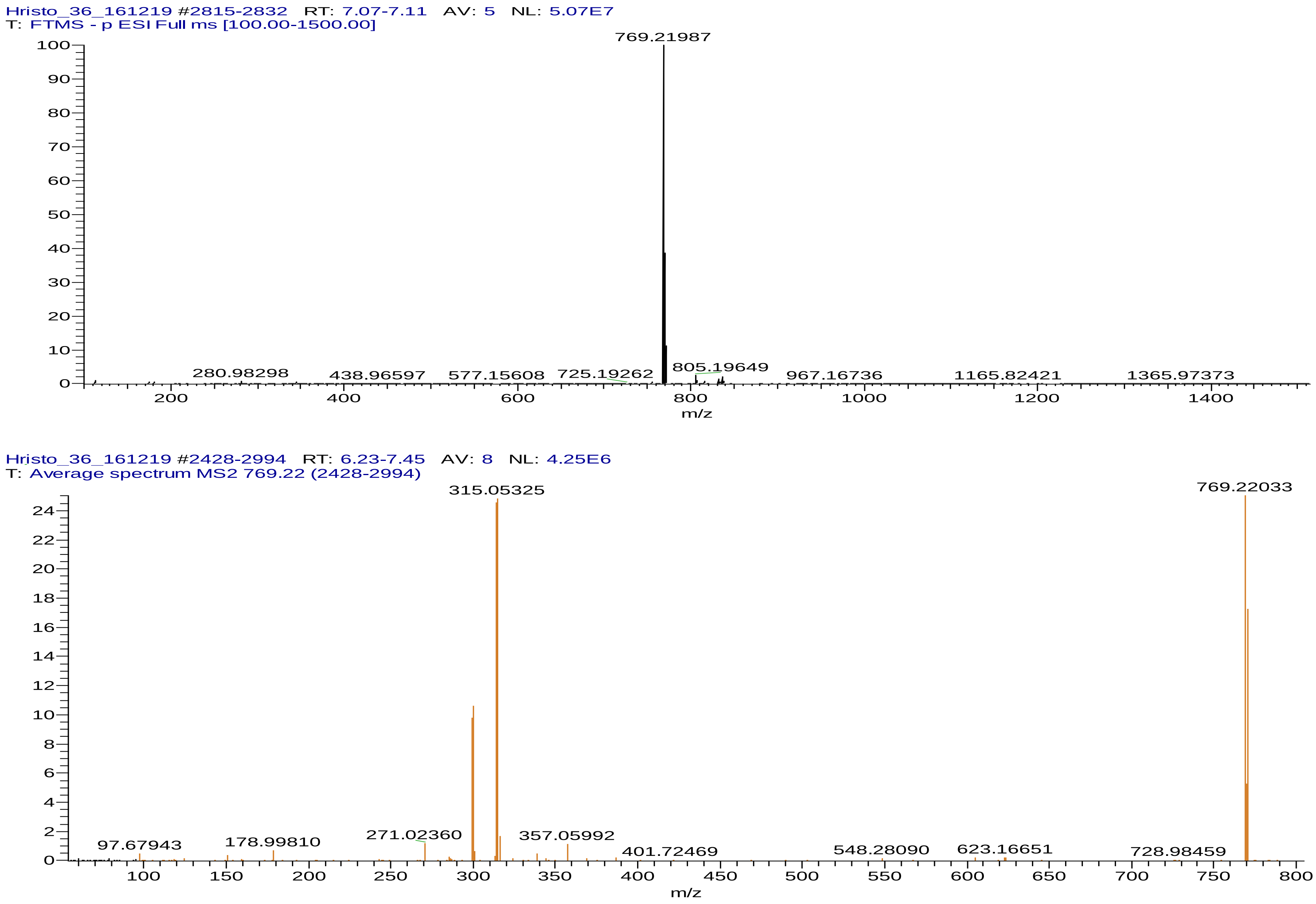

Figure S28: HR-ESI-MS (ESI negative mode) spectrum of compound **3**

Up: full-scan

Down: MS2 of 769.21987 m/z

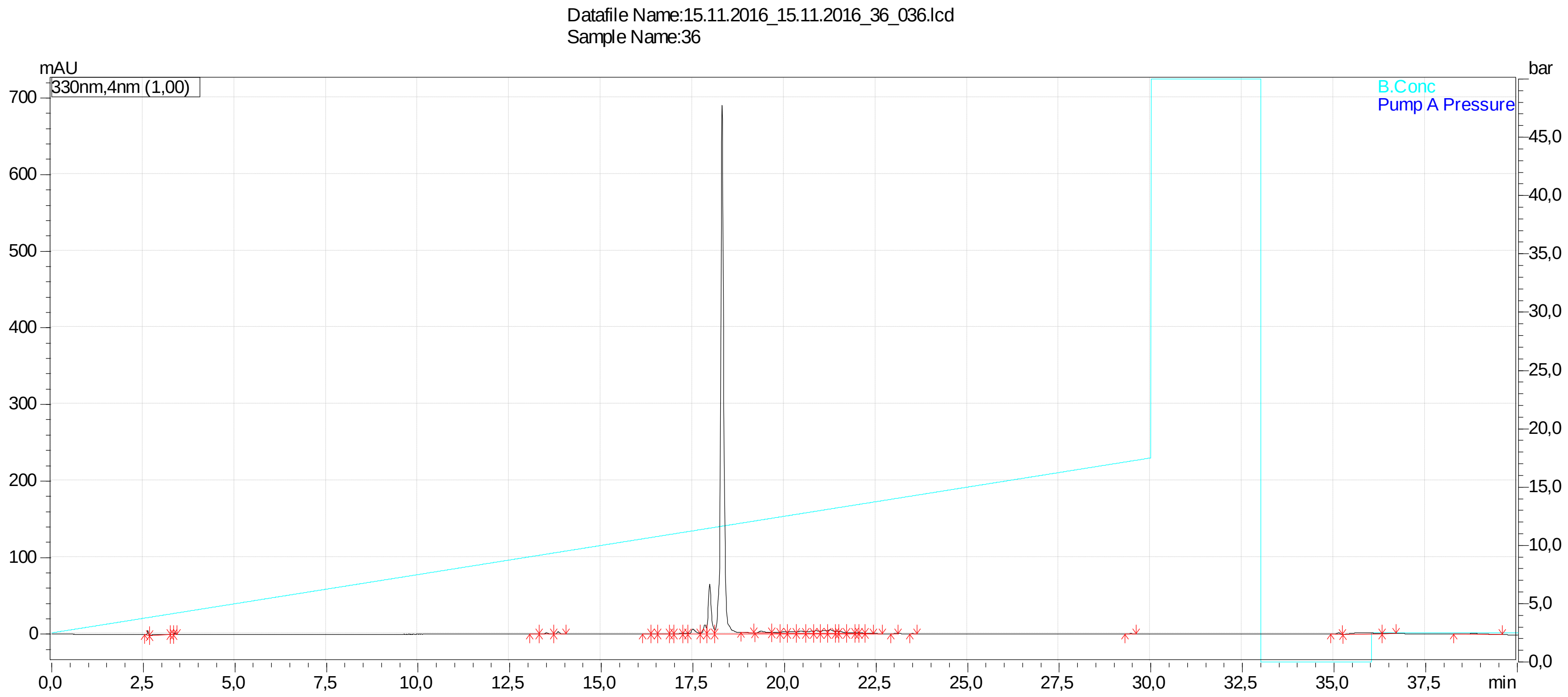

Figure S29: HPLC chromatogram of compound **3**

Gradient 5 → 35%; Solvent A (CH_3CN); Solvent B (H_2O + 0.02% HCOOH);

Column: Kinetex® PFP 100 A, 250 x 4.6 mm I.D., 5 µm (Phenomenex, USA); Flow: 1 ml/min

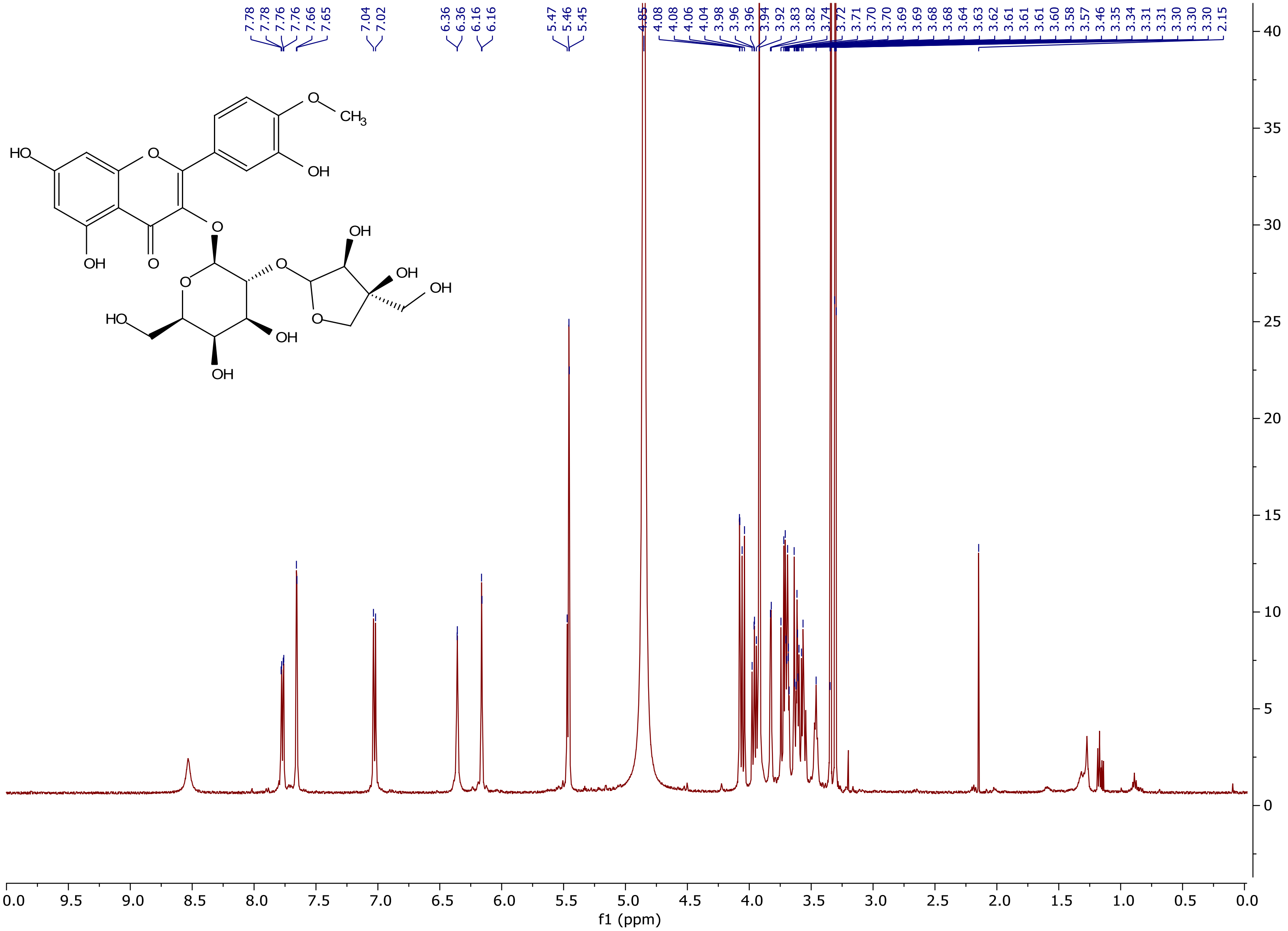

Figure S30: ^{1}H NMR (600 MHz, CD_3OD; 299 °K) spectrum of compound **4**

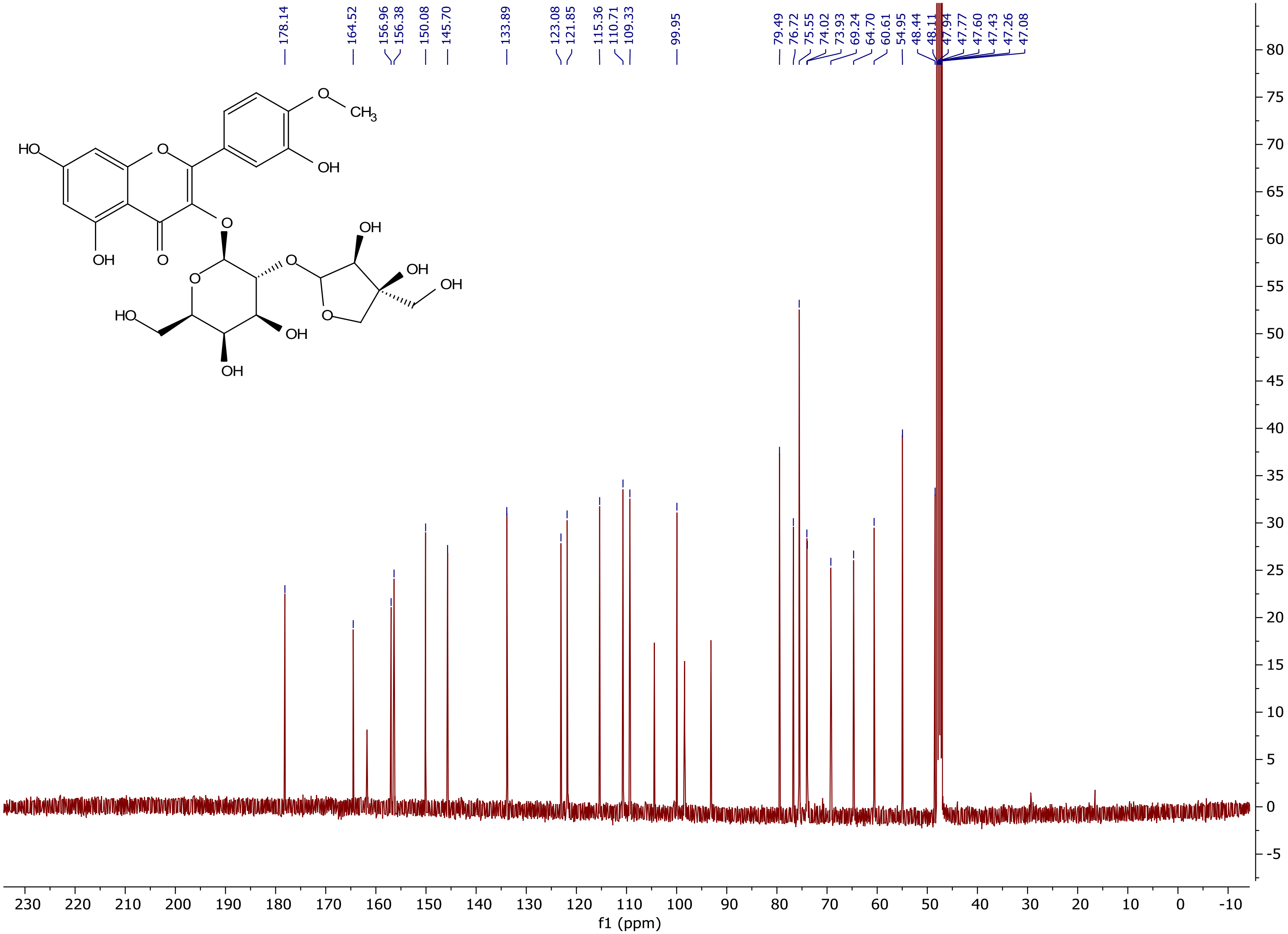

Figure S31: ^{13}C NMR (150 MHz, CD_3OD; 299 °K) spectrum of compound **4**

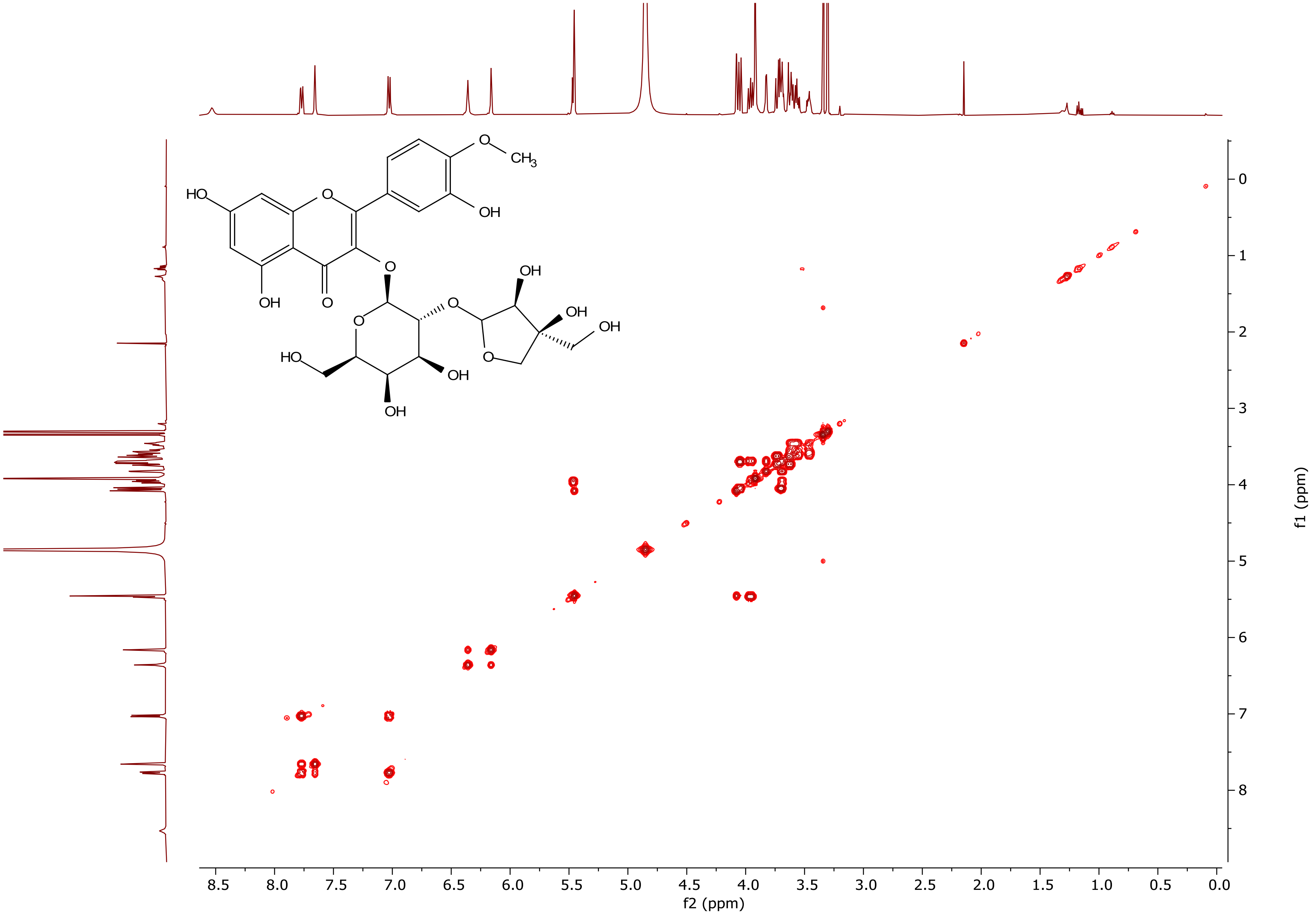

Figure S32: COSY spectrum of compound **4**

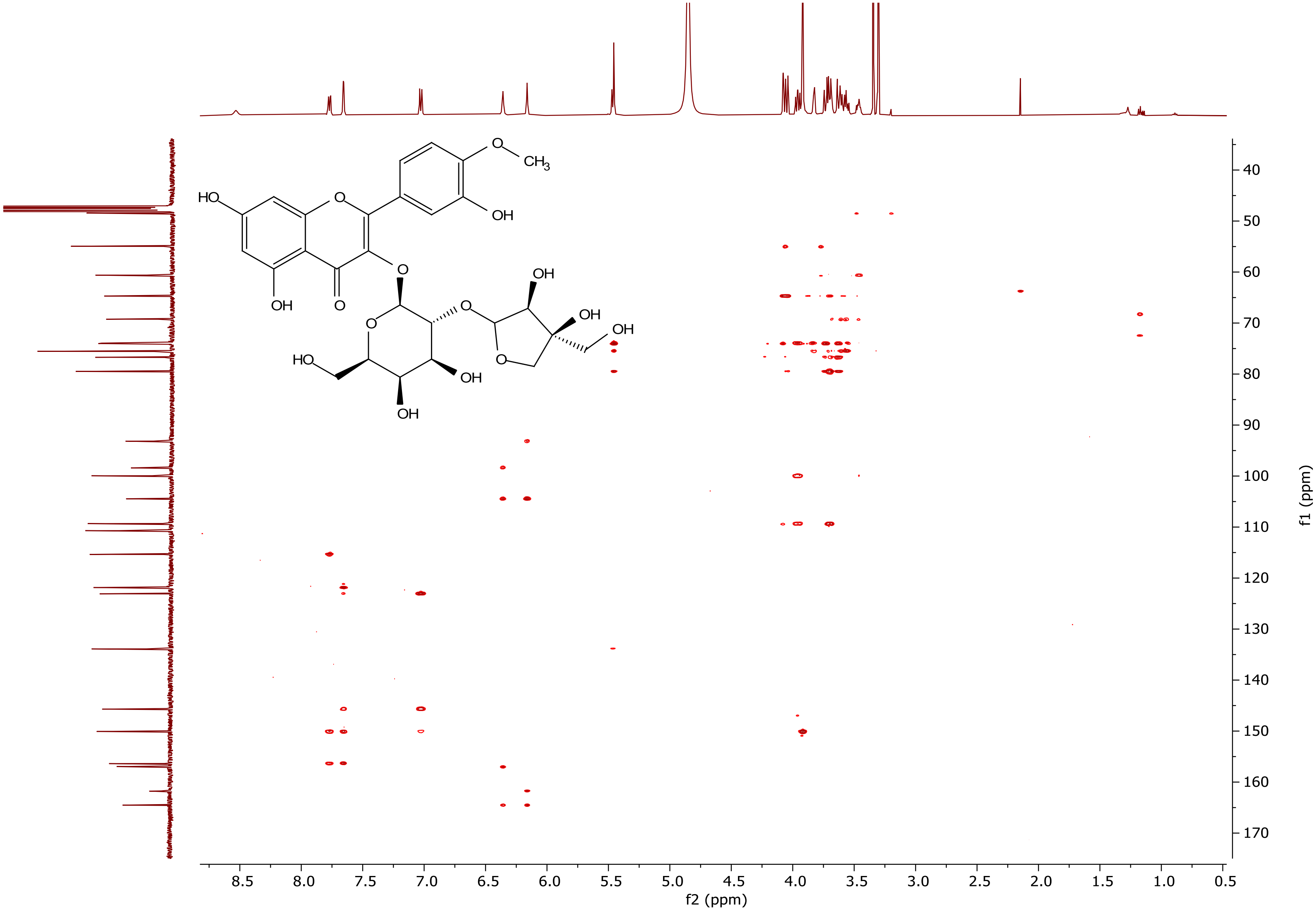

Figure S33: HMBC Spectrum of compound **4**

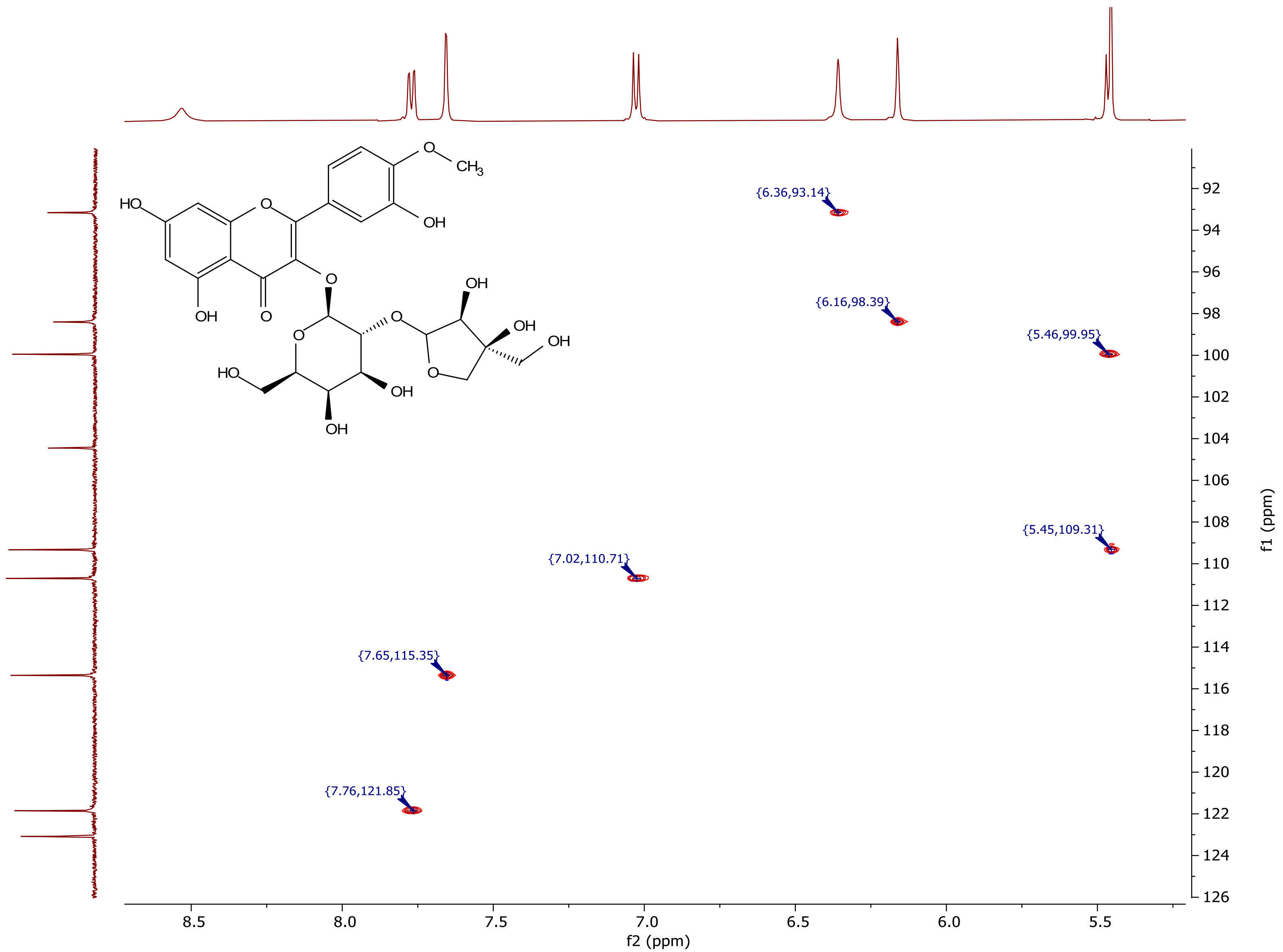

Figure S34: HSQC spectrum of aglycon and anomeric region of compound **4**

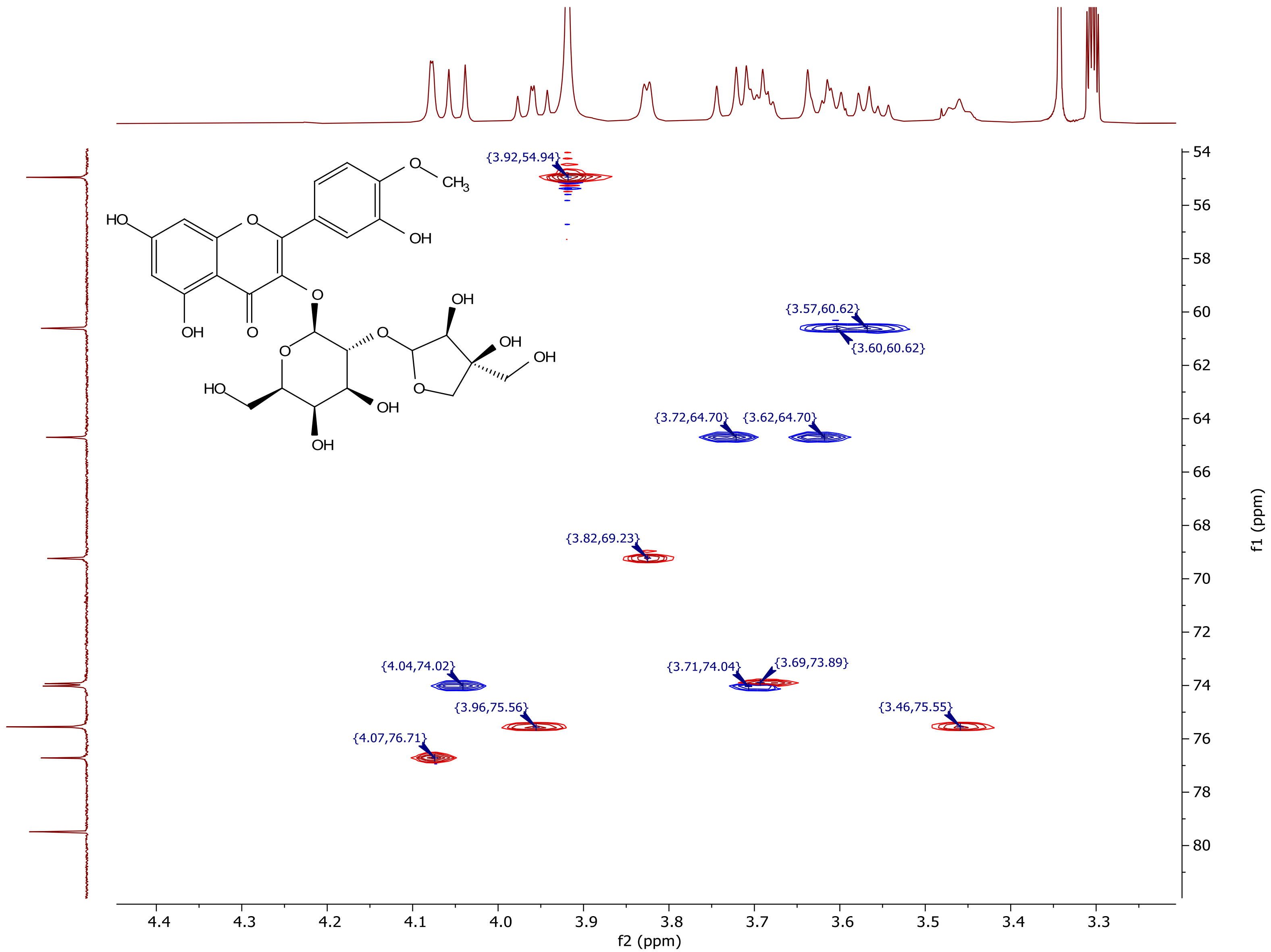

Figure S35: HSQC spectrum of sugars region of compound **4**

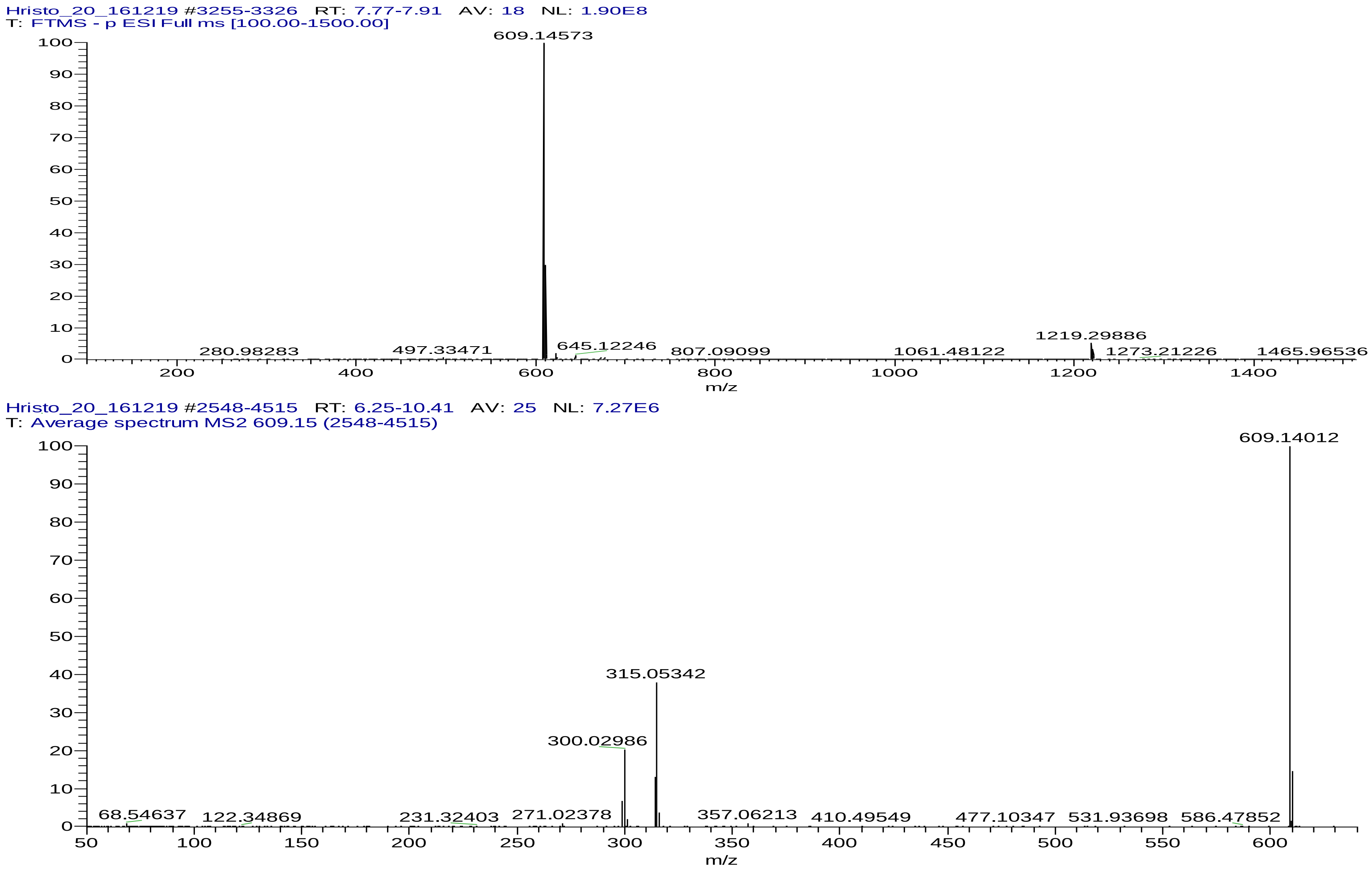

Figure S36: HR-ESI-MS (ESI negative mode) spectrum of compound **4**

Up: full-scan

Down: MS2 of 609.14573 m/z

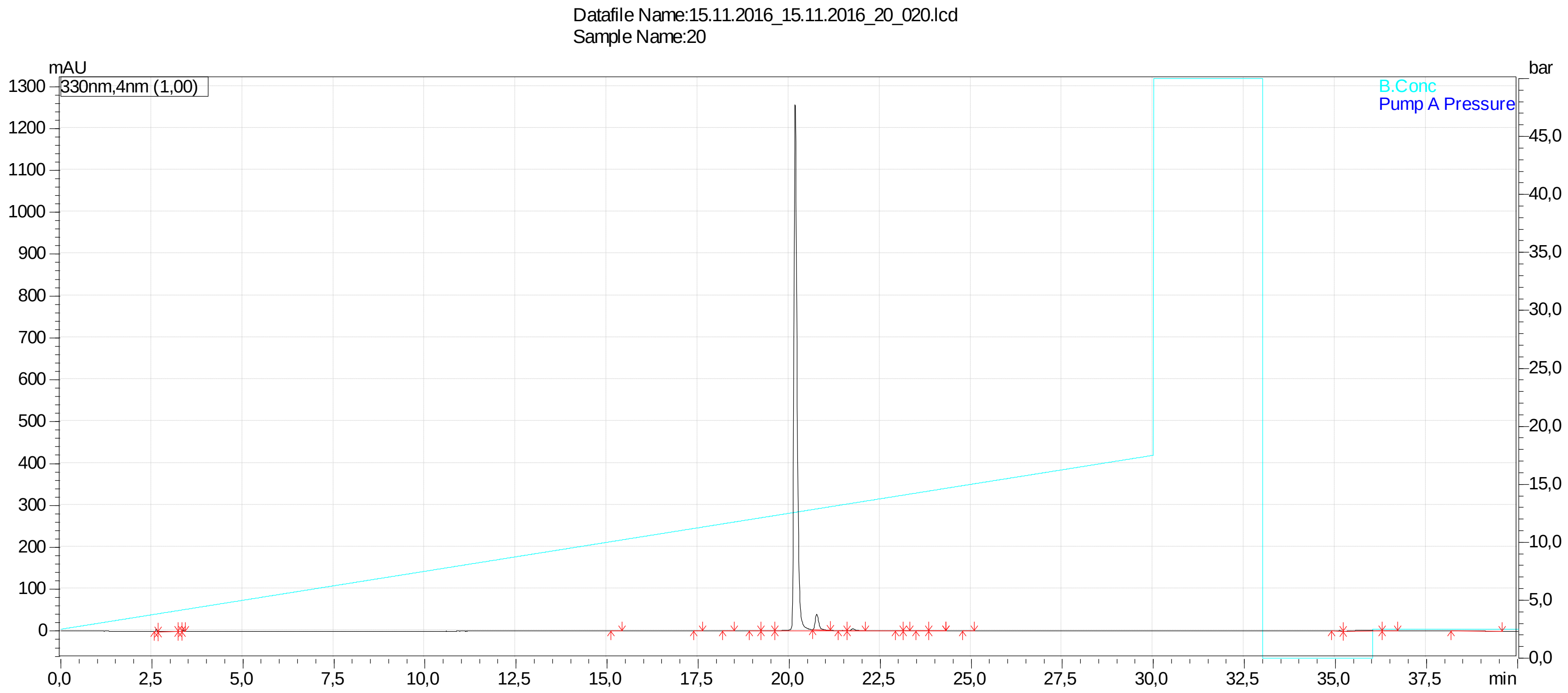

Figure S37: HPLC chromatogram of compound **4**

Gradient 5 → 35%; Solvent A (CH_3CN); Solvent B (H_2O + 0.02% HCOOH);

Column: Kinetex® PFP 100 A, 250 x 4.6 mm I.D., 5 µm (Phenomenex, USA); Flow: 1 ml/min

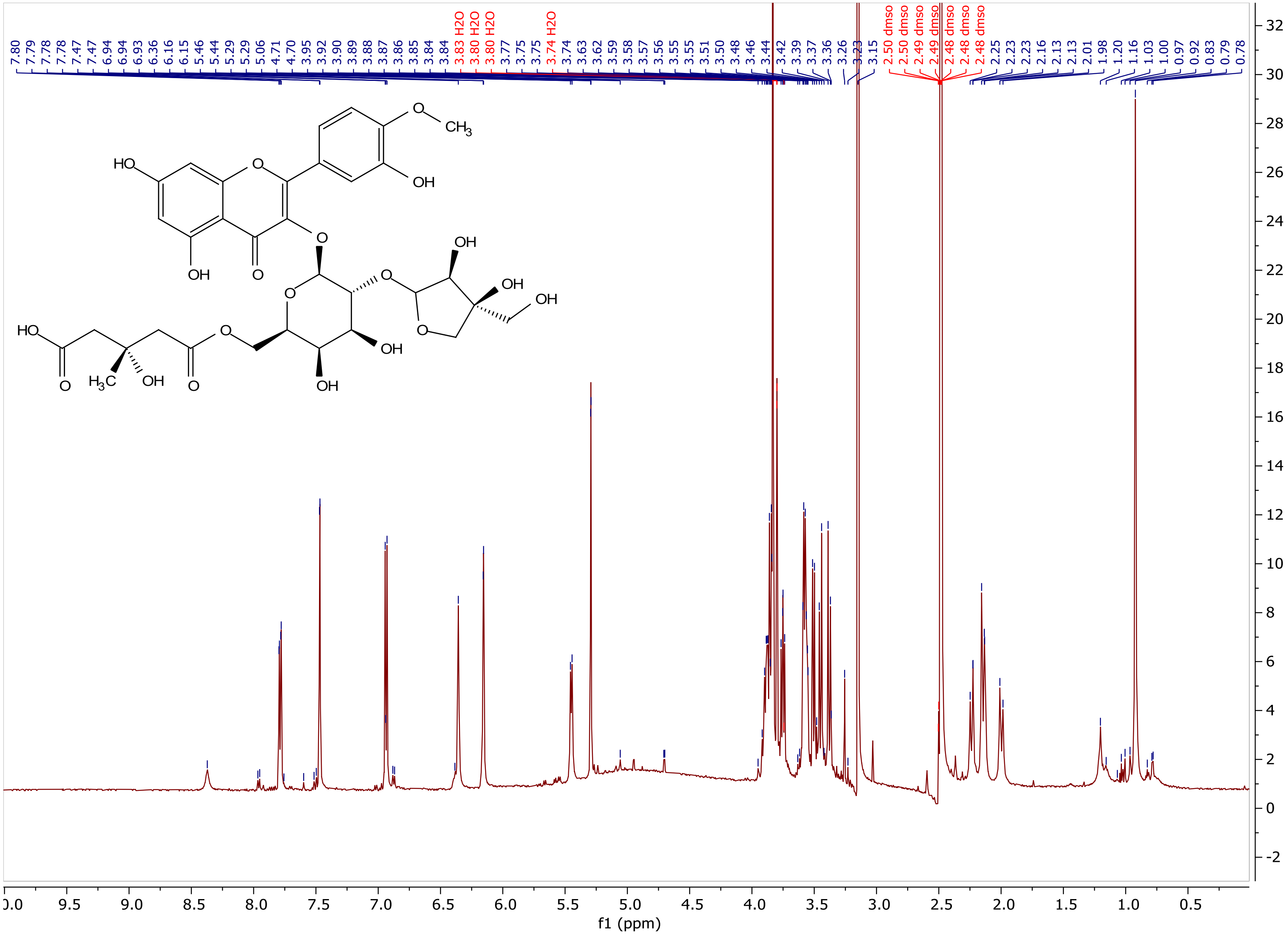

Figure S38: ^{1}H NMR (600 MHz, DMSO; 299 °K) spectrum of compound **5**

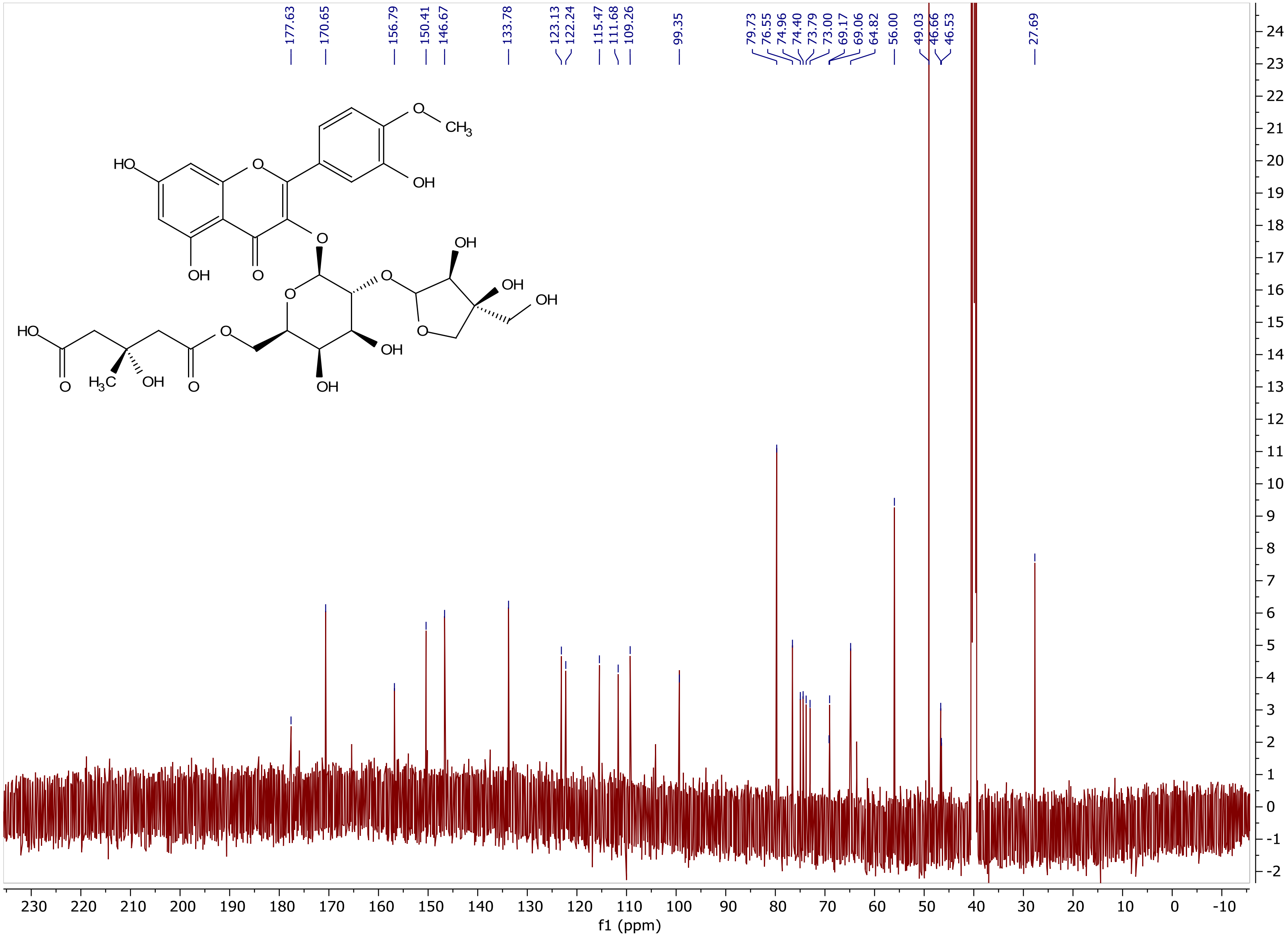

Figure S39: ^{13}C NMR (150 MHz, DMSO; 299 °K) spectrum of compound **5**

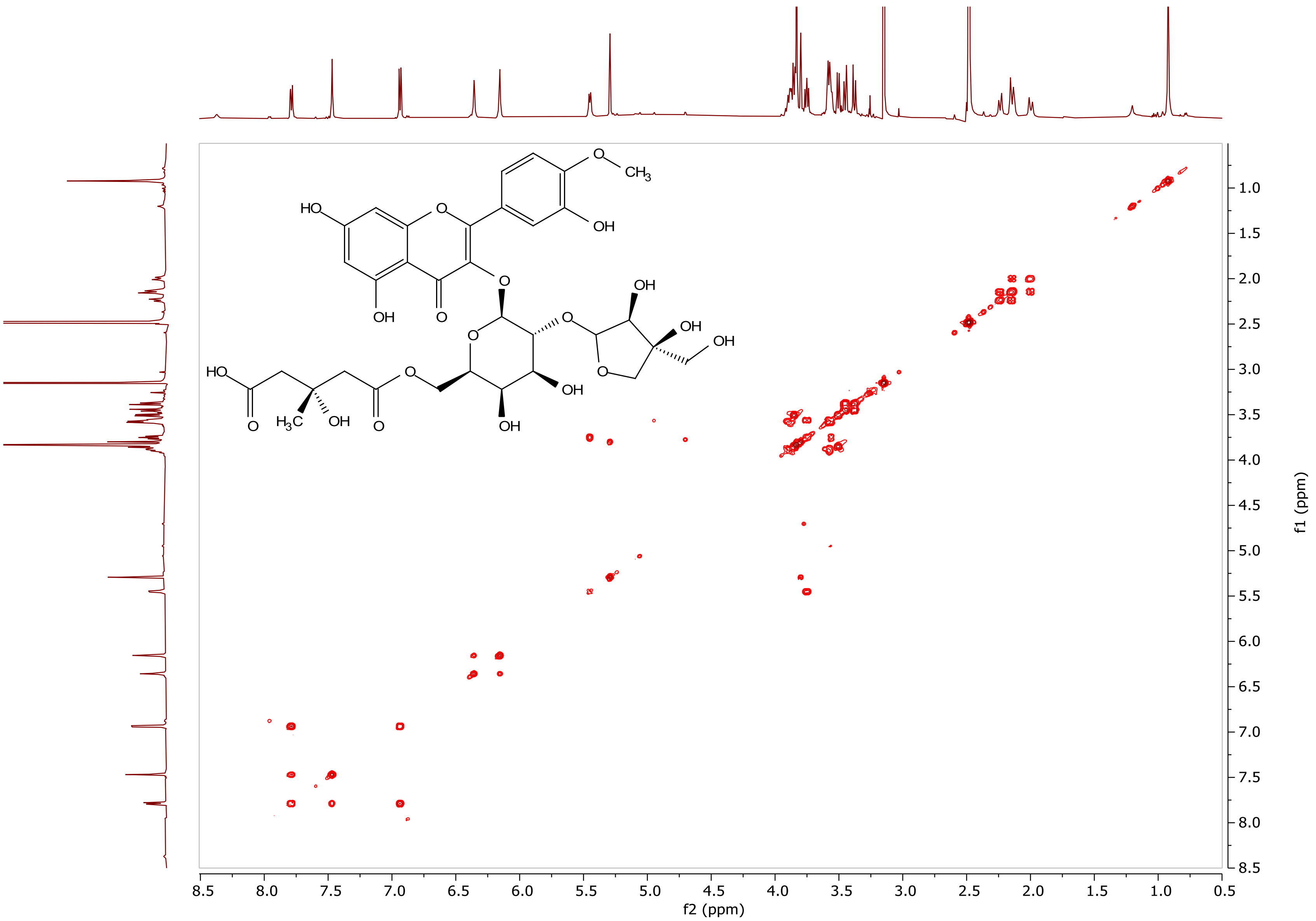

Figure S40: COSY spectrum of compound **5**

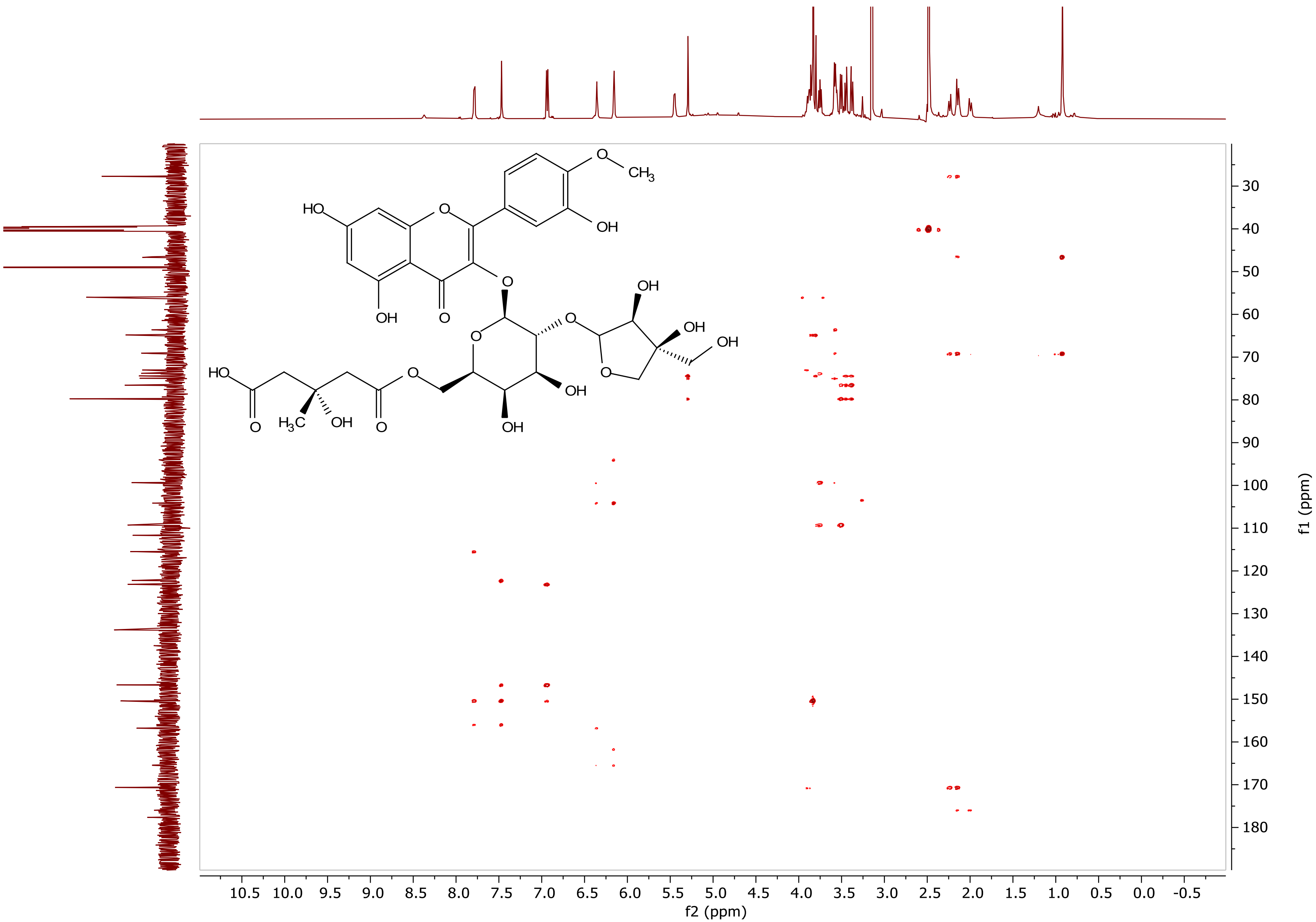

Figure S41: HMBC Spectrum of compound **5**

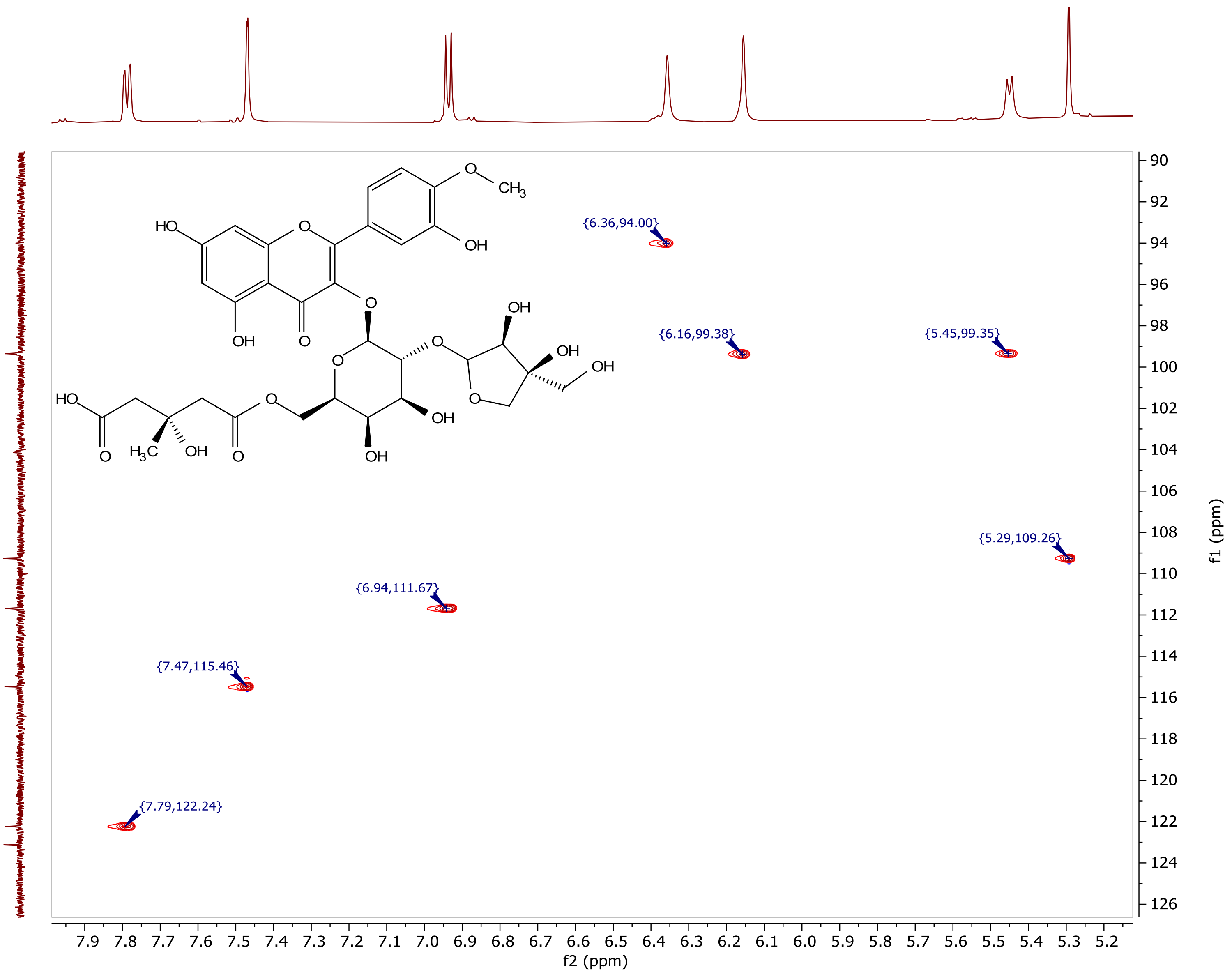

Figure S42: HSQC spectrum of aglycon and anomeric region of compound **5**

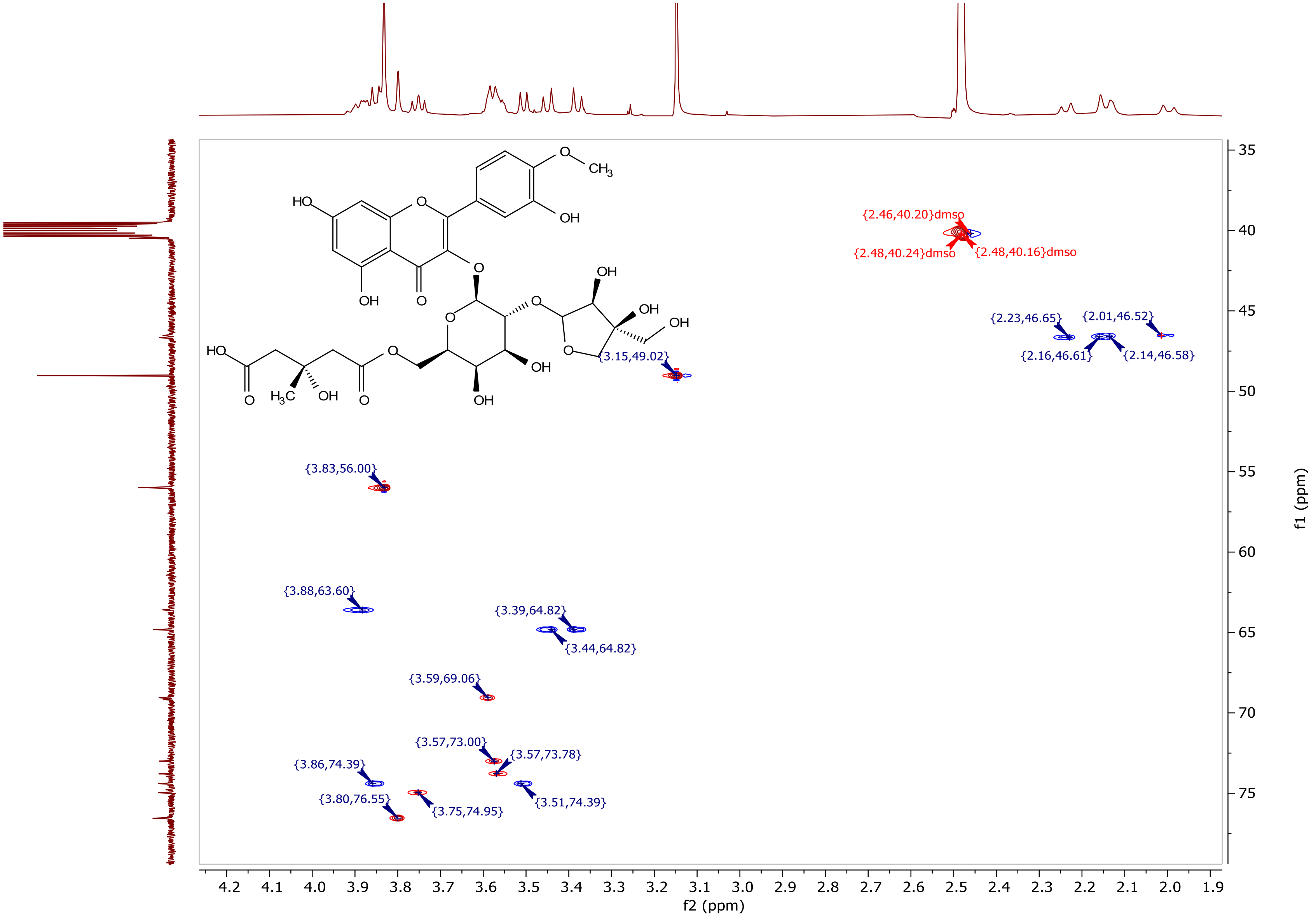

Figure S43: HSQC spectrum of sugars region of compound **5**

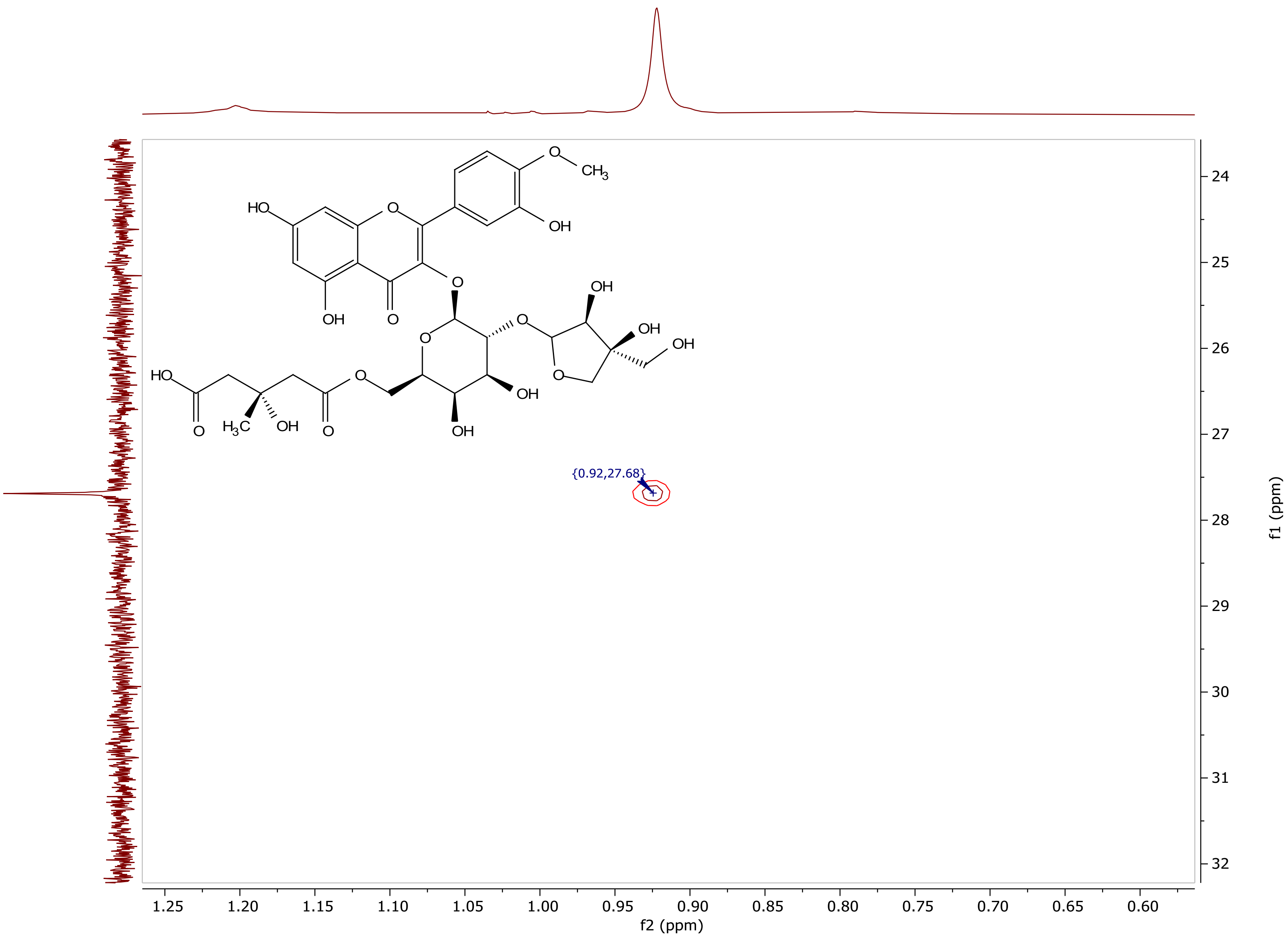

Figure S44: HSQC spectrum CH_3 group on C-4' of compound **5**

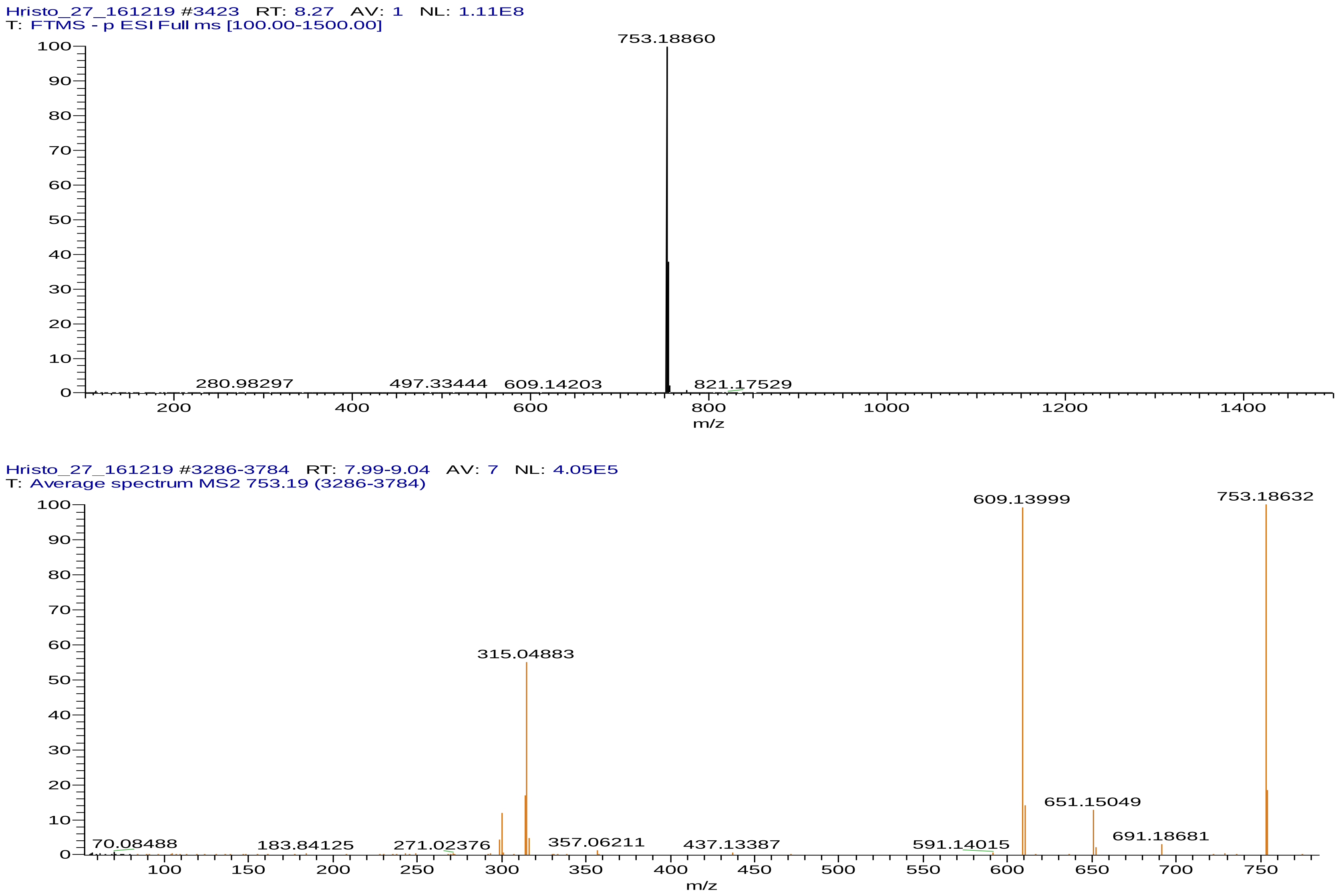

Figure S45: HR-ESI-MS (ESI negative mode) spectrum of compound **5**

Up: full-scan

Down: MS2 of 753.18860 m/z

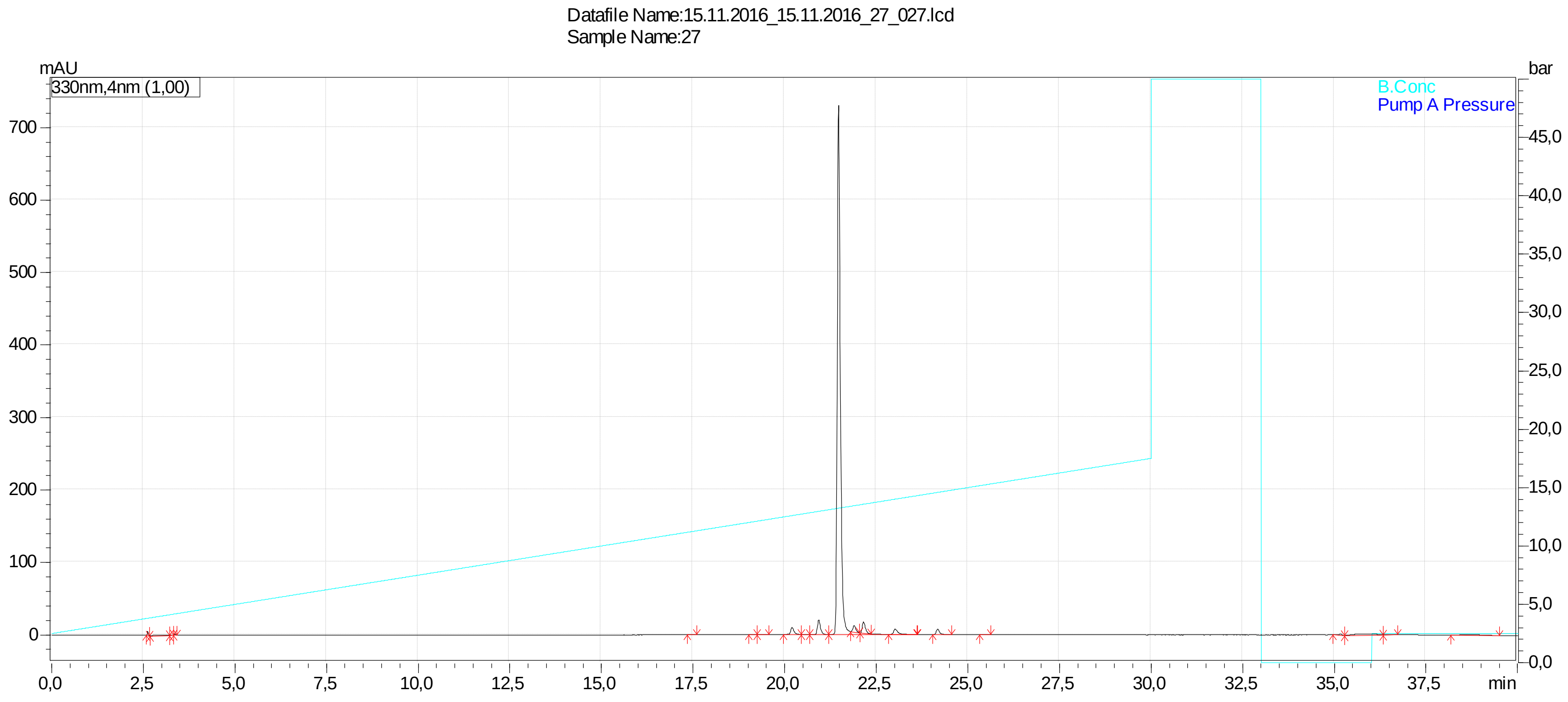

Figure S46: HPLC chromatogram of compound **5**

Gradient 5 → 35%; Solvent A (CH_3CN); Solvent B (H_2O + 0.02% HCOOH);

Column: Kinetex® PFP 100 A, 250 x 4.6 mm I.D., 5 µm (Phenomenex, USA); Flow: 1 ml/min

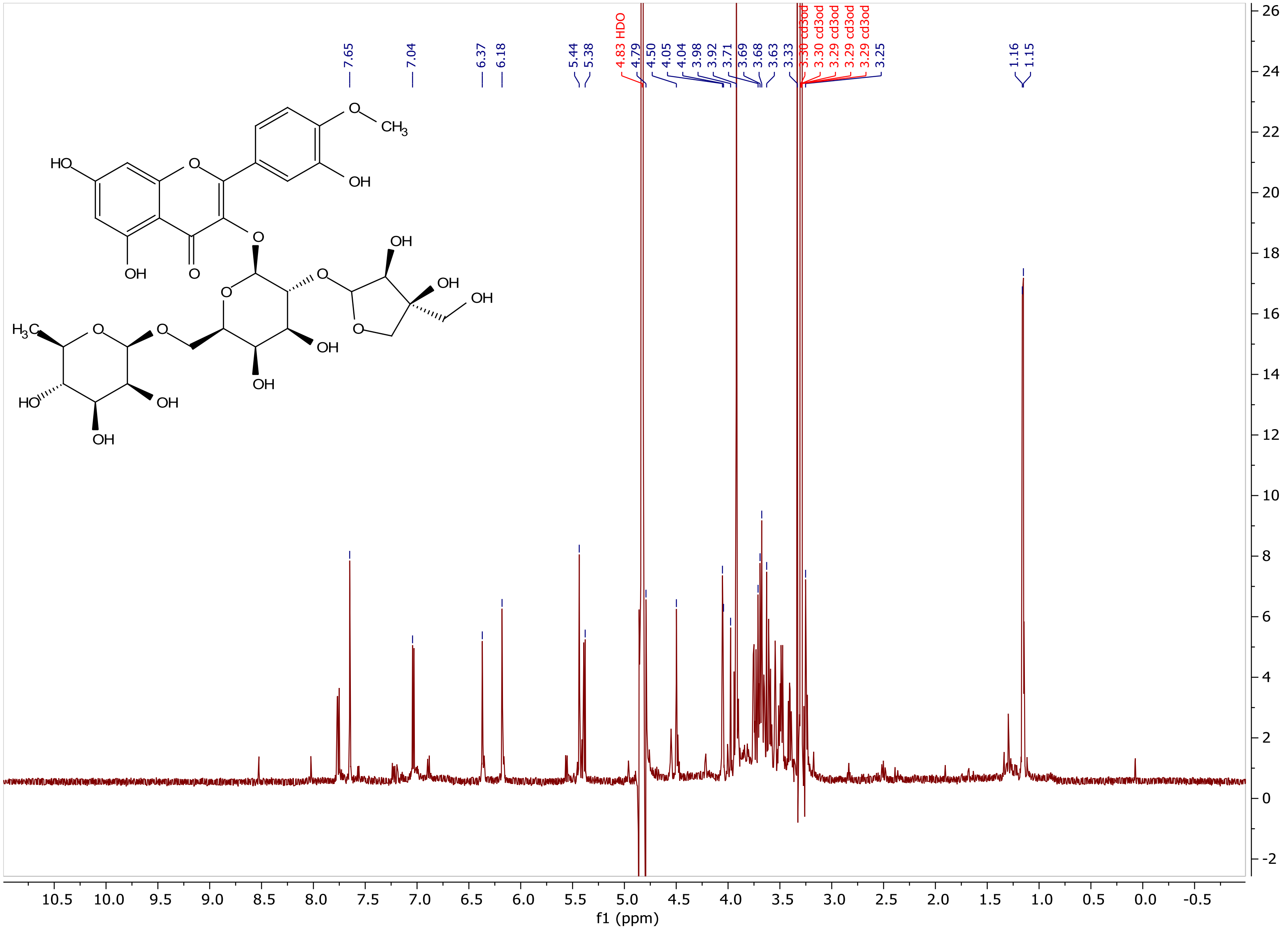

Figure S47: ^{1}H NMR (600 MHz, CD_3OD; 299 °K) spectrum of compound **6**

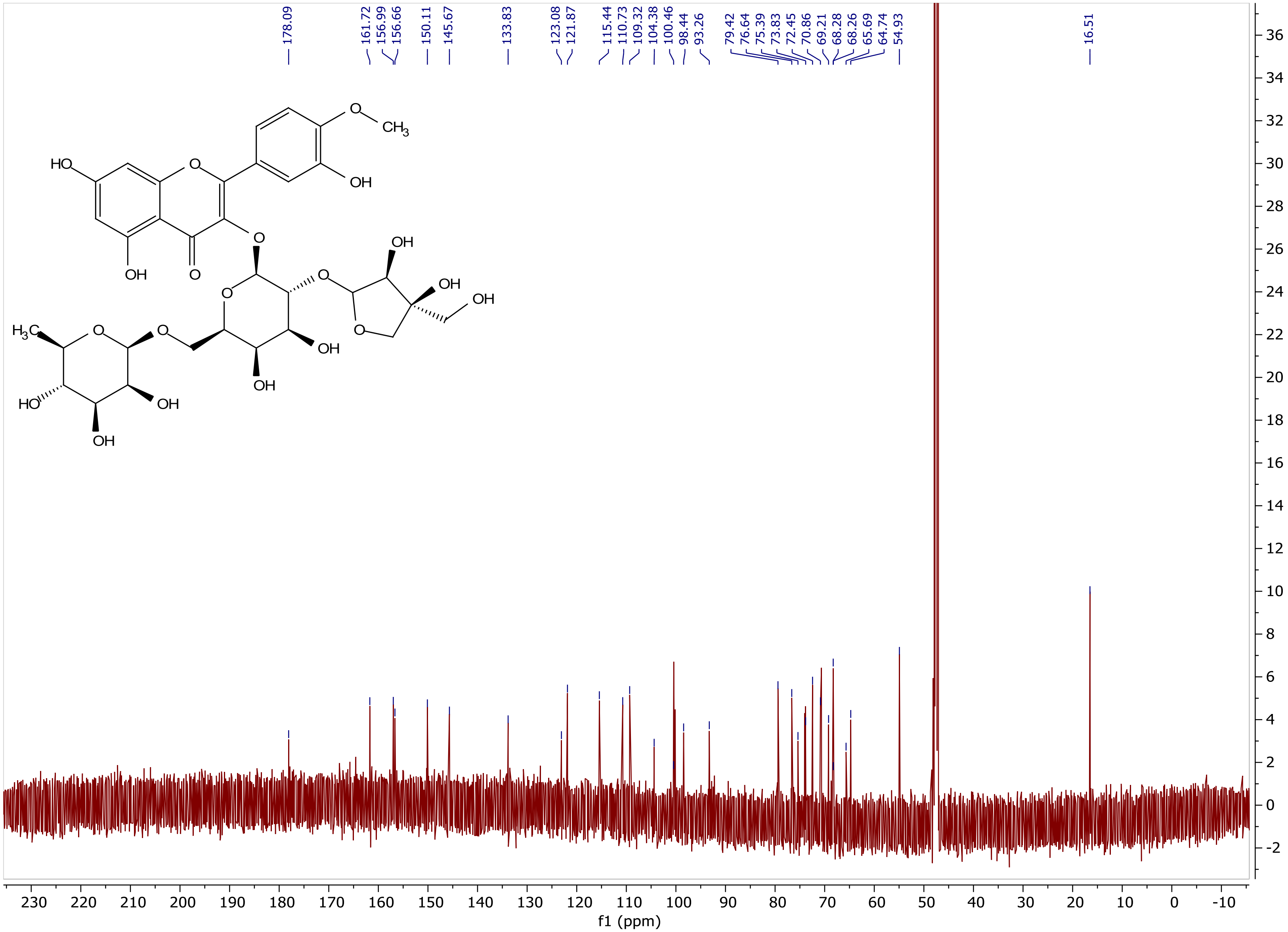

Figure S48: ^{13}C NMR (150 MHz, CD_3OD; 299 °K) spectrum of compound **6**

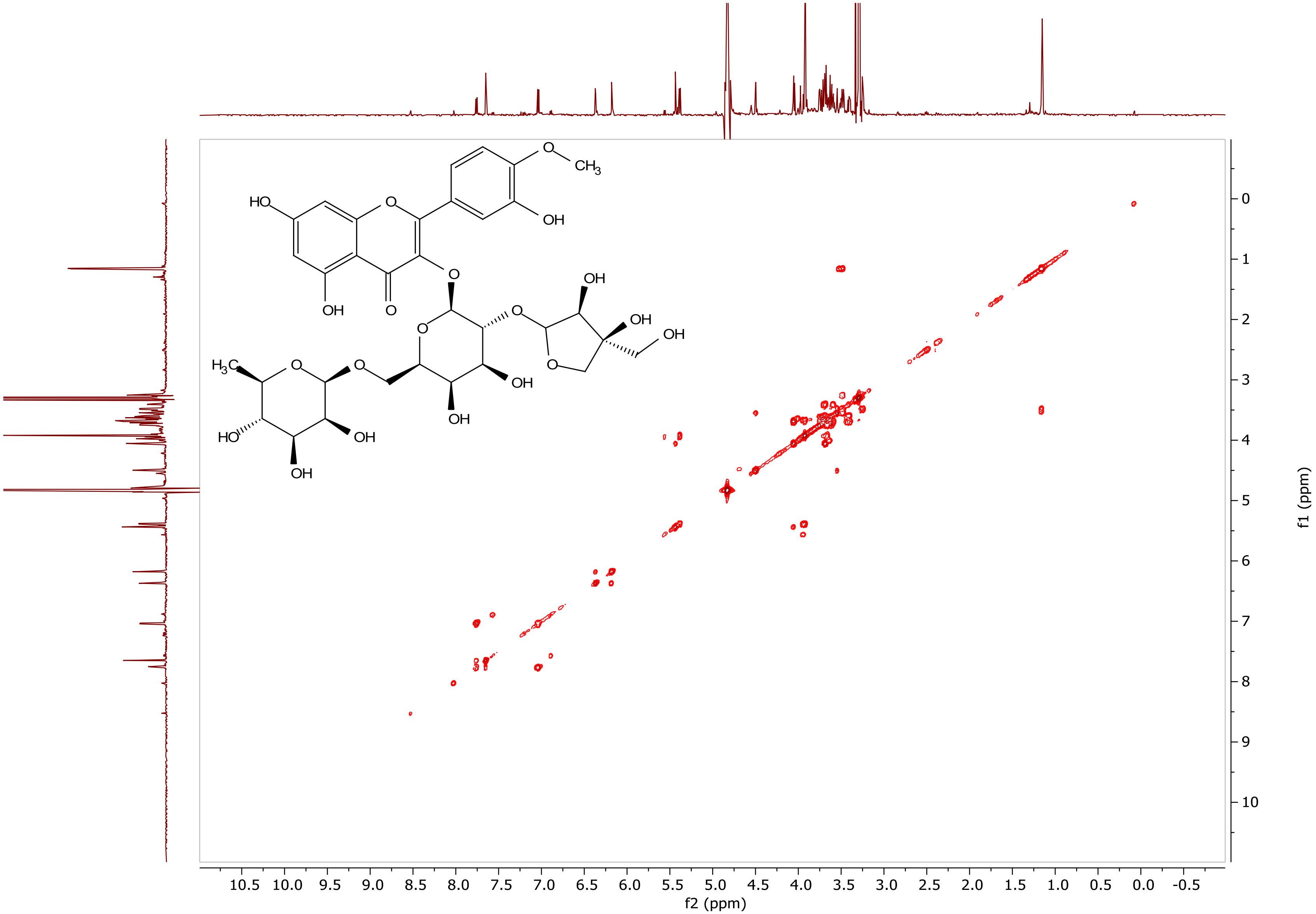

Figure S49: COSY spectrum of compound **6**

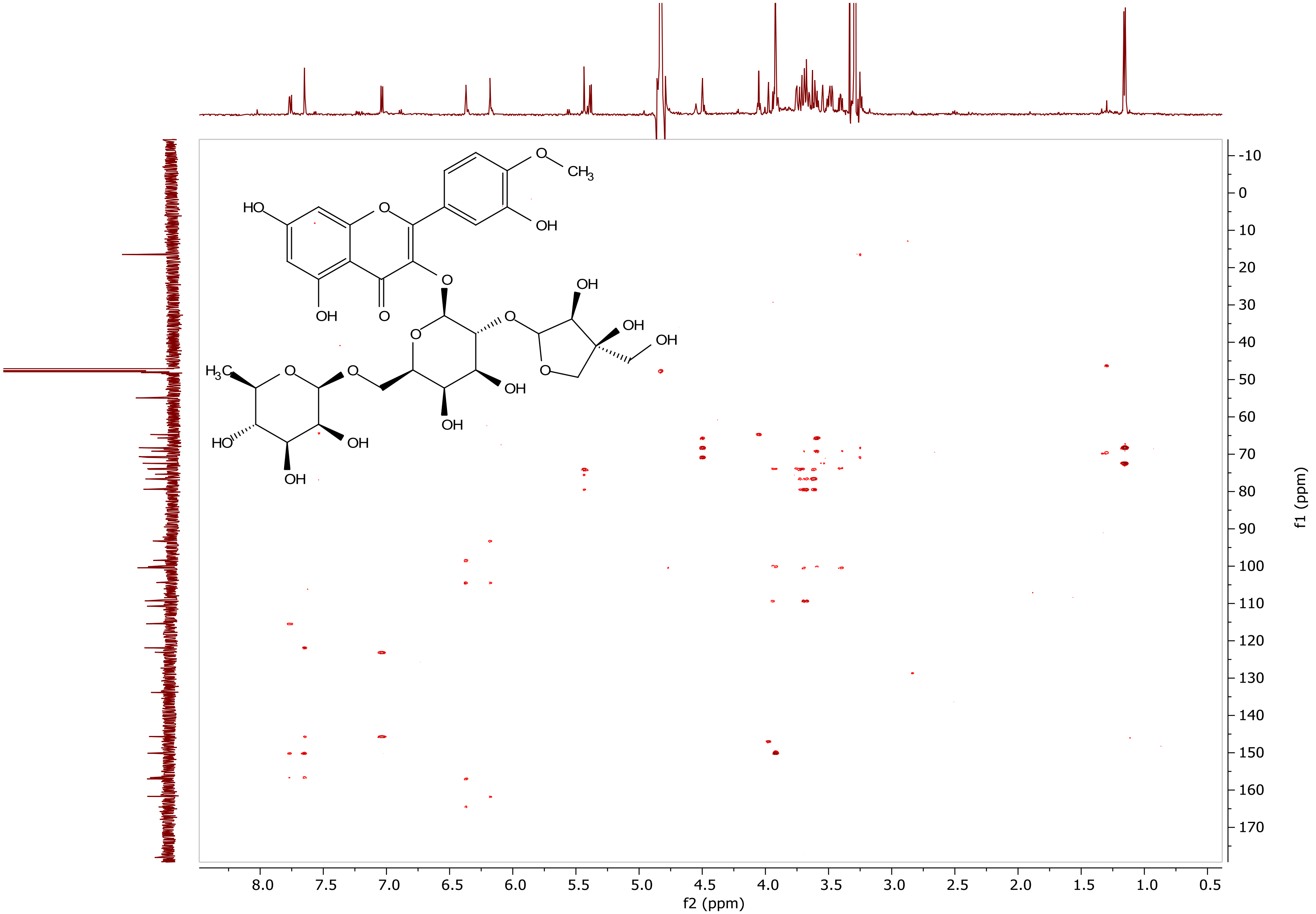

Figure S50: HMBC Spectrum of compound **6**

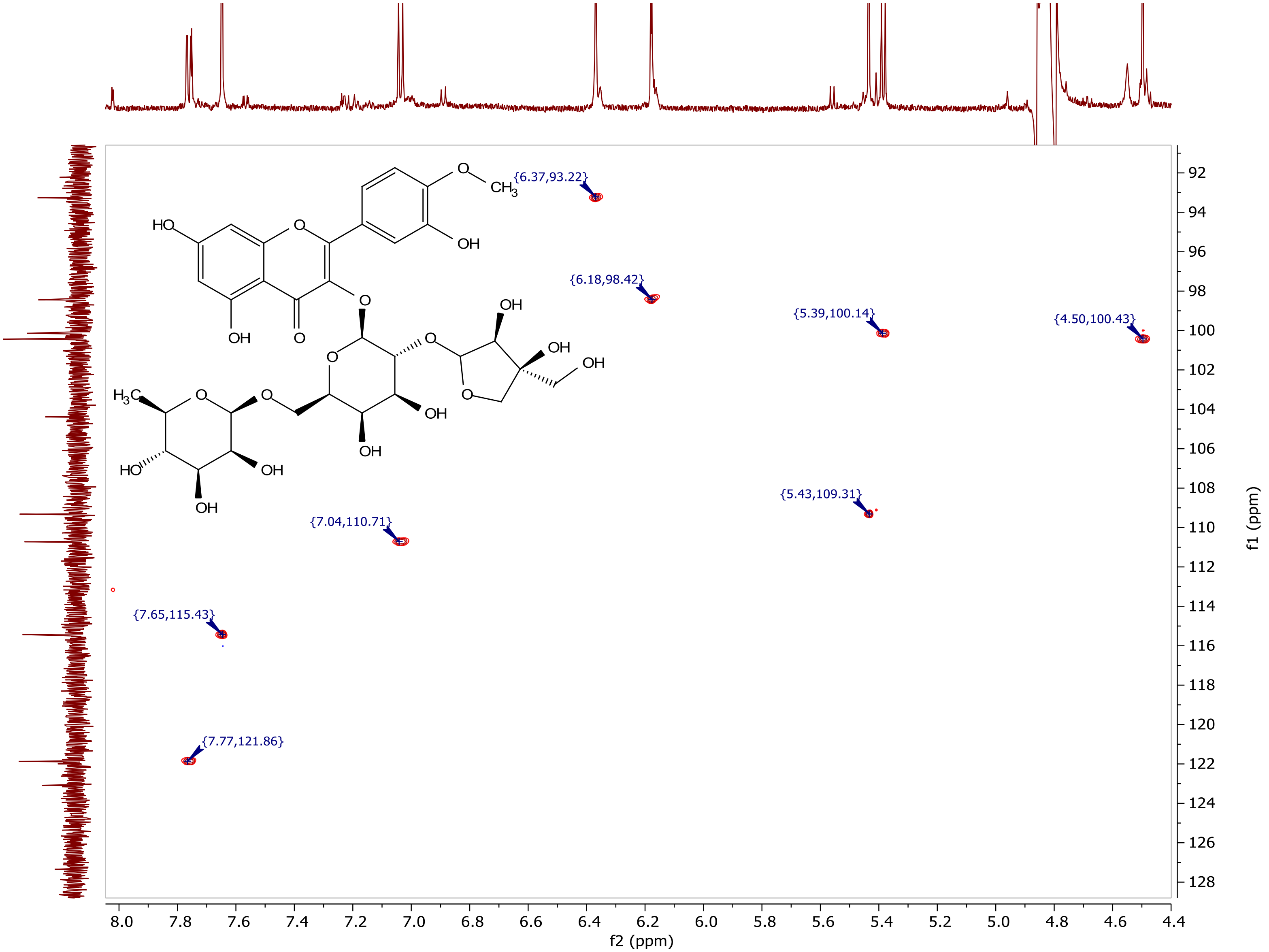

Figure S51: HSQC spectrum of aglycon and anomeric region of compound **6**

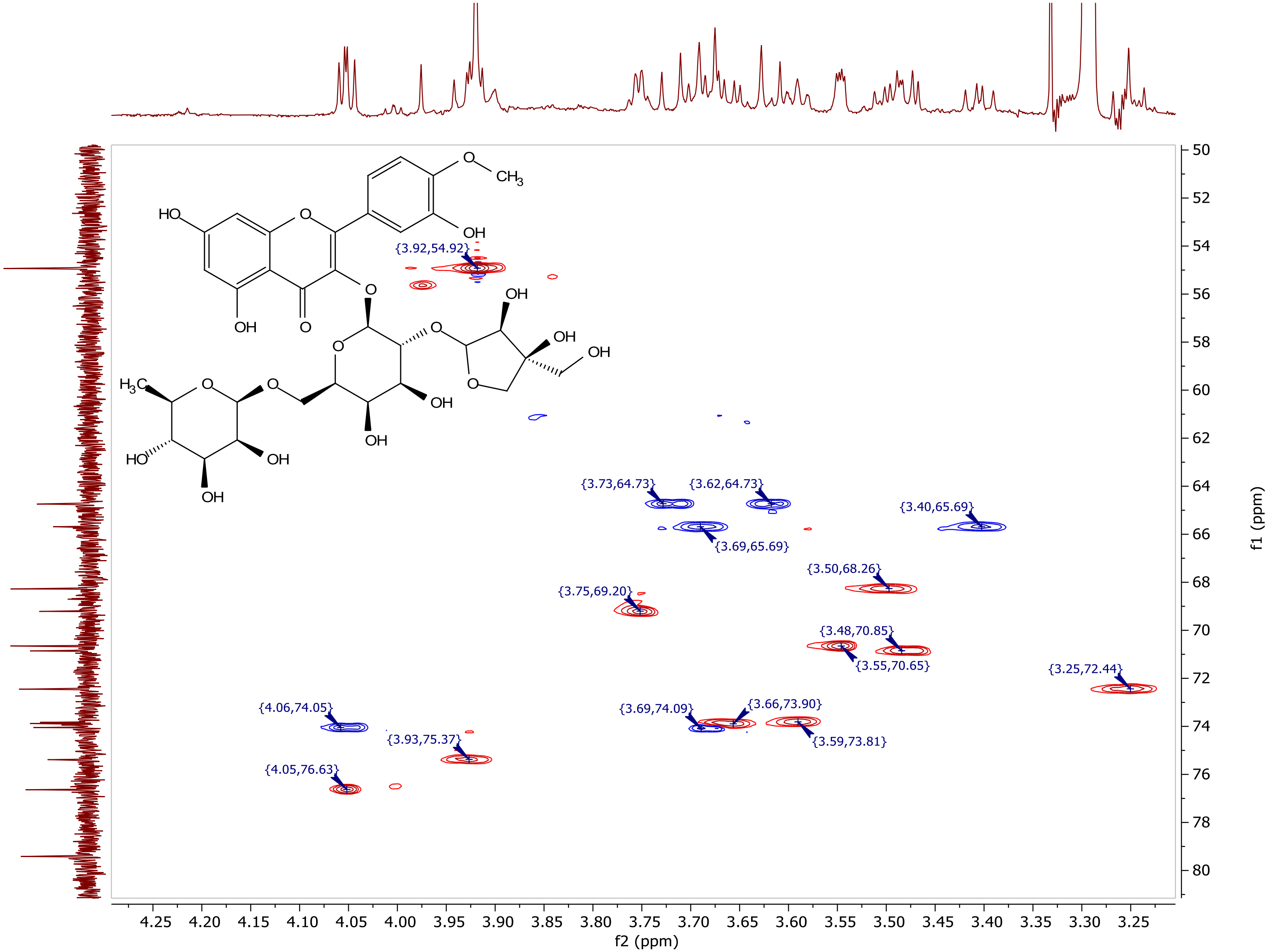

Figure S52: HSQC spectrum of sugars region of compound **6**

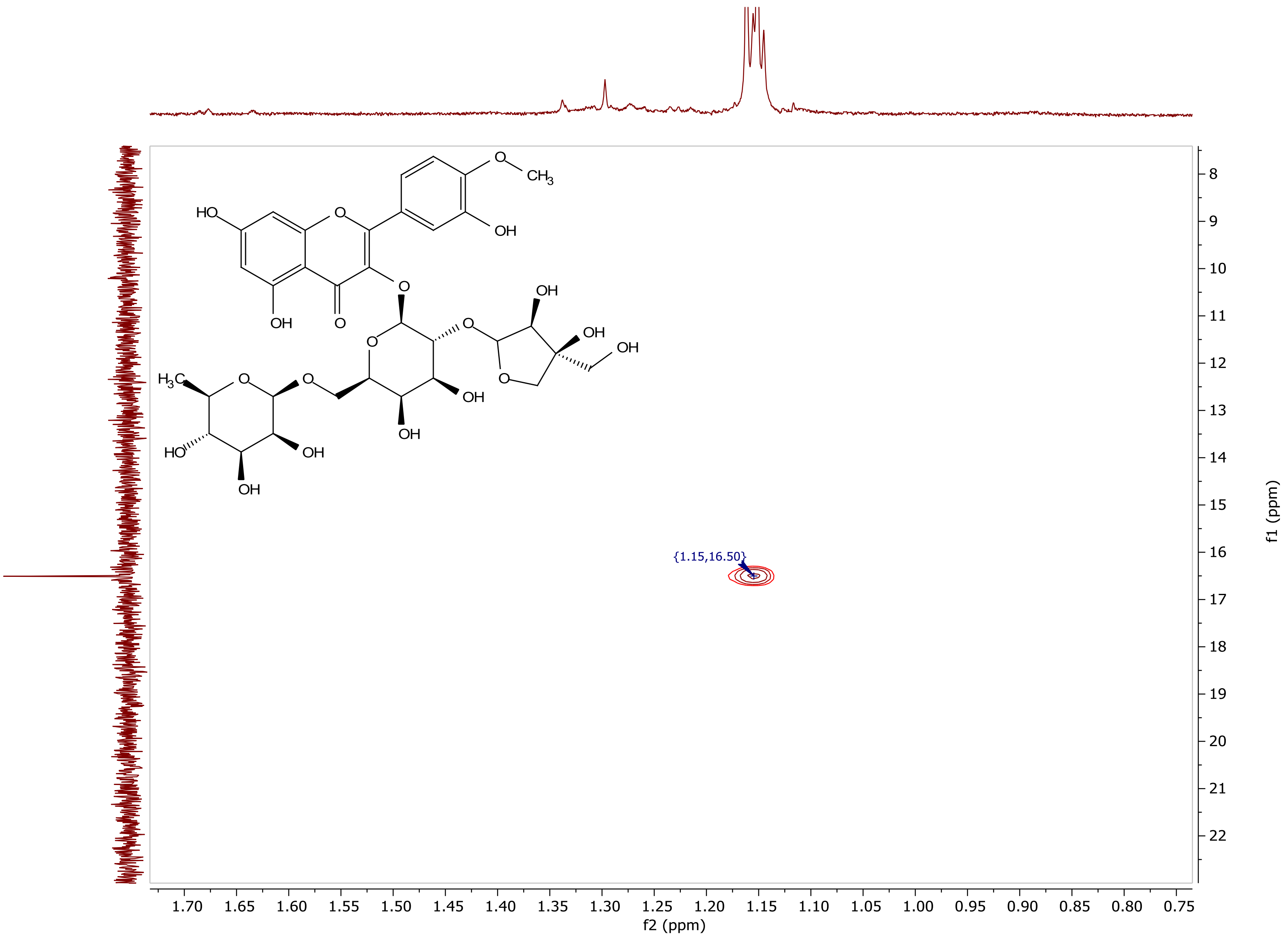

Figure S53: HSQC spectrum CH_3 of rhamnose group of compound **6**

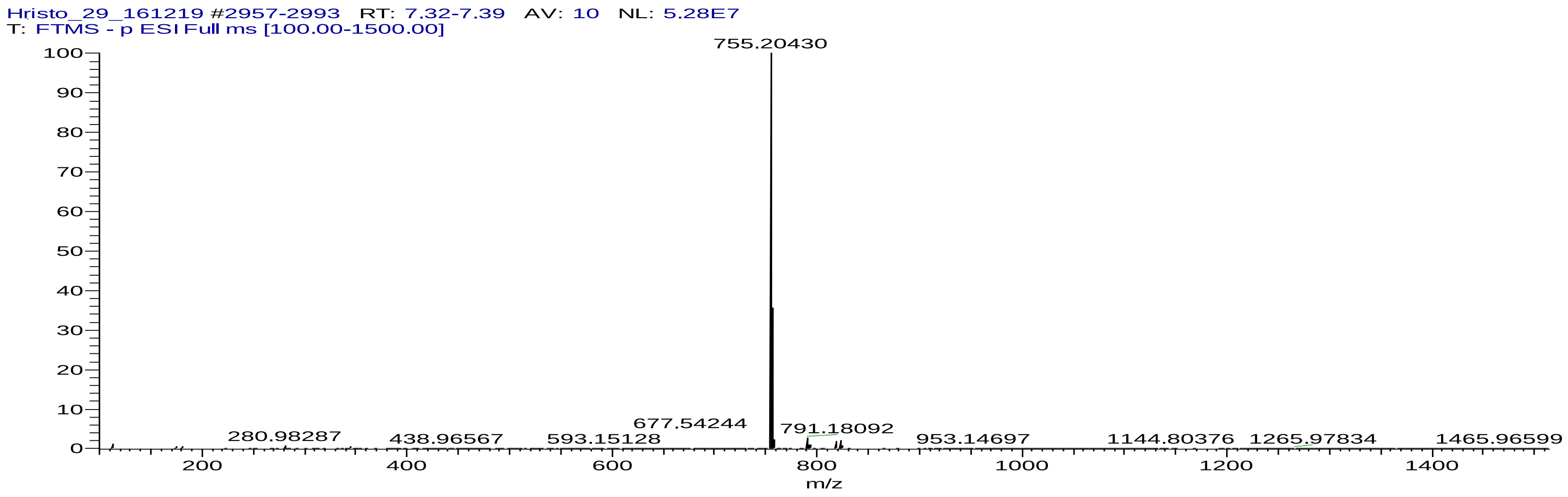

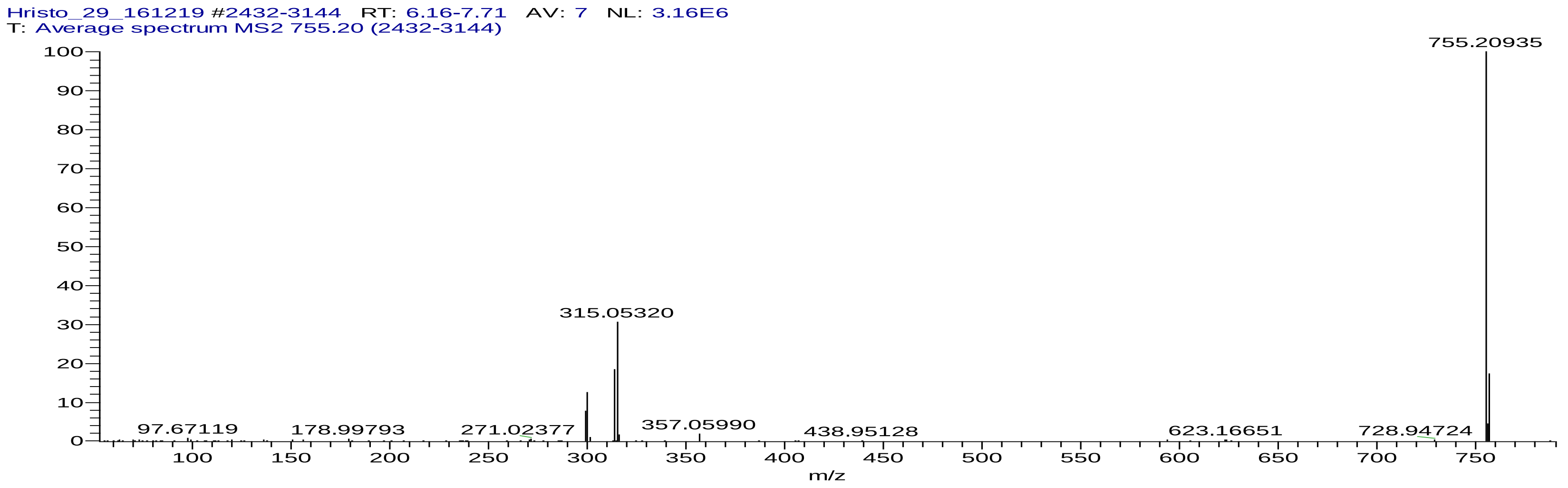

Figure S54: HR-ESI-MS (ESI negative mode) spectrum of compound **6**

Up: full-scan

Down: MS2 of 755.20430 m/z

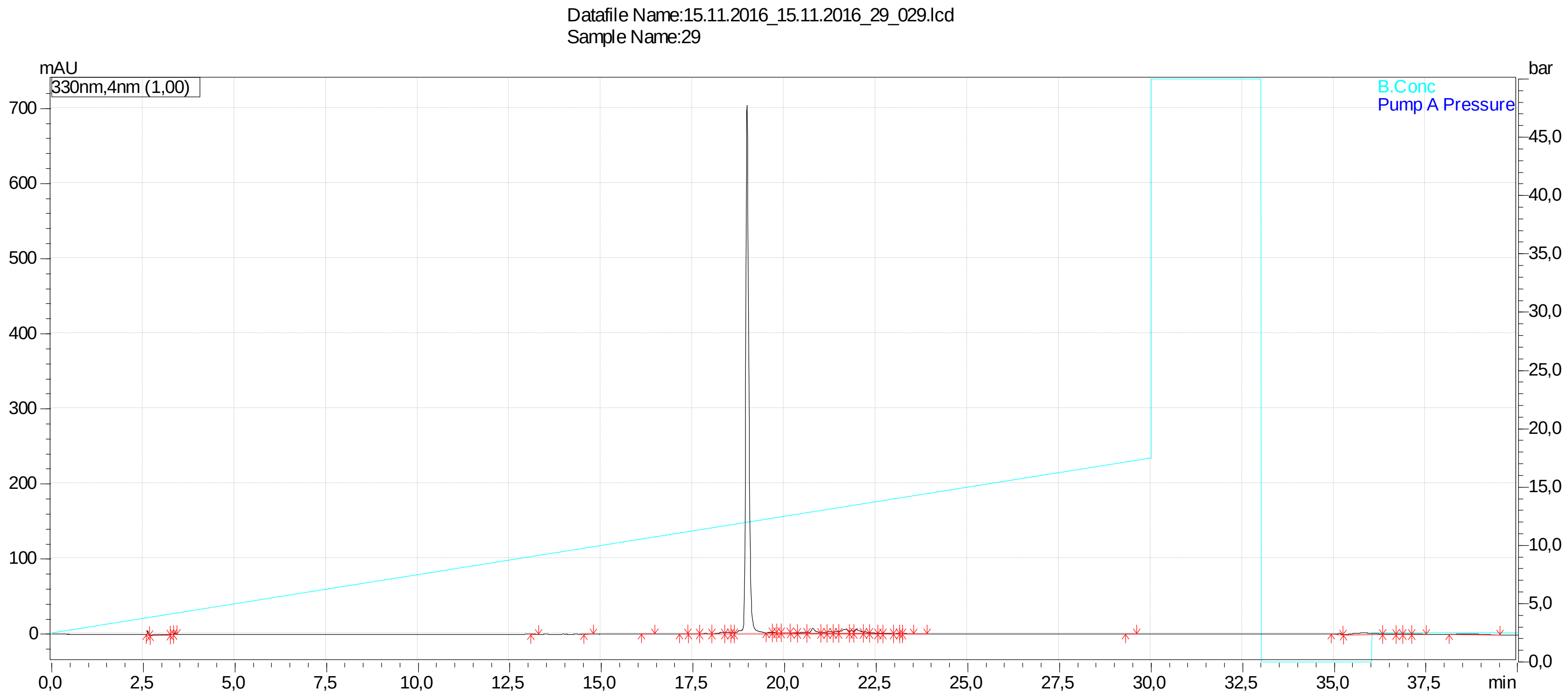

Figure S55: HPLC chromatogram of compound **6**
Gradient 5 → 35%; Solvent A (CH_3CN); Solvent B (H_2O + 0.02% HCOOH);
Column: Kinetex® PFP 100 A, 250 x 4.6 mm I.D., 5 µm (Phenomenex, USA); Flow: 1 ml/min

Tab. S1. Binding affinities of tested ligands with collagenase

Plant Source	Ligand	Binding afinity (kcal/mol)
A. aitosensis	Isorhamnetin-3-O-[β-D-apiofuranosyl-(1→2)-[α-L-rhamnopyranosyl-(1→6)]-β-D-galactopyranosyl]-7-O-β-D-glucopyranoside	-8.0
	Isorhamnetin-3-O-[β-D-glucopyranosyl-(1→5)-β-D-apiofuranosyl]-(1→2)-robinobioside or [α-L-rhamnopyranosyl-(1→6)]-β-D-galactopyranosyl]	-8.4
	Isorhamnetin-3-O-(2,6-di-O-α-rhamnopyranosyl-β-D-galactopyranoside)-7-O-β-D-glucopyranoside	-8.3
	Isorhamnetin-3-O-robinobioside-7-O-glucoside	-7.7
	Isorhamnetin-3-O-robinobioside	-7.8
	Isorhamnetin-3-O-(2,6-di-O-α-rhamno-pyranosyl-β-galactopyranoside)	-7.9
	Alangiflavosid	-8.6
A. thracicus	Tamarixetin-3-*O*-(2,6-di-*O*-α-L-rhamnopyranosyl)-β-D-galactopyranoside (**3**)	-7.5
	Tamarixetin-3-*O*-α-L-rhamnopyranosyl-(1→2)-[6-*O*-(3-hydroxy-3-methylglutaryl)]-β-D-galactopyranoside (**2**)	-7.7
	tamarixetin-3-*O*-β-D-apiofuranosyl-(1→2)-[α-L-rhamnopyranosyl-(1→6)]-β-D-galactopyranoside (**6**)	-7.5
	Tamarixetin-3-*O*-β-D-apiofuranosyl-(1→2)-[6-*O*-(3-hydroxy-3-methylglutaryl)]-β-D-galactopyranoside (**5**)	-7.6
	Tamarixetin 3-*O*-β-D-apiofuranosyl-(1→2)-β-D-galactopyranoside (**4**)	-6.6
	Kaempferol-3-*O*-α-L-rhamnopyranosyl-(1→2)-[6-*O*-(3-hydroxy-3-methylglutaryl)]-β-D-galactopyranoside (**1**)	-8.0
	Mauritianin	-8.1
	Kaempferol-3-*O*-α-L-rhamnopyranosyl-(1→2)-β-galactopyranoside	-7.7
	Kaempferol-3-*O*-β-apiofuranosyl-(1→2)-β-galactopyranoside	-7.5
	Quercetin-3-*O*-β-apiofuranosyl-(1→2)-β-galactopyranoside	-7.3
A. gombiformis	Kaempferol-3-O-α-L-rha-(1→2)[6-O-(3-hydroxy-3-methylglutaryl)]-β-D-gal	-8.7
-	Isoamylphosphonyl-Gly-Pro-Ala	-6.4
-	Chlorogenic acid	-6.6

Tab. S2. Binding affinities of tested ligands with elastase

Plant Source	Ligand	Binding afinity (kcal/mol)
A. aitosensis	Isorhamnetin-3-O-[β-D-apiofuranosyl-(1→2)-[α-L-rhamnopyranosyl-(1→6)]-β-D-galactopyranosyl]-7-O-β-D-glucopyranoside	-8.9
	Isorhamnetin-3-O-[β-D-glucopyranosyl-(1→5)-β-D-apiofuranosyl]-(1→2)-robinobioside or [α-L-rhamnopyranosyl-(1→6)]-β-D-galactopyranosyl]	-8.3
	Isorhamnetin-3-O-(2,6-di-O-α-rhamnopyranosyl-β-D-galactopyranoside)-7-O-β-D-glucopyranoside	-8.5
	Isorhamnetin-3-O-robinobioside-7-O-glucoside	-7.5
	Isorhamnetin-3-O-robinobioside	-8.1
	Isorhamnetin-3-O-(2,6-di-O-α-rhamno-pyranosyl-β-galactopyranoside)	-7.7
	Alangiflavosid	-8.3
A. thracicus	Tamarixetin-3-O-(2,6-di-O-α-L-rhamnopyranosyl)-β-D-galactopyranoside (**3**)	-8.1
	Tamarixetin-3-O-α-L-rhamnopyranosyl-(1→2)-[6-O-(3-hydroxy-3-methylglutaryl)]-β-D-galactopyranoside (**2**)	-7.4
	tamarixetin-3-O-β-D-apiofuranosyl-(1→2)-[α-L-rhamnopyranosyl-(1→6)]-β-D-galactopyranoside (**6**)	-7.6
	Tamarixetin-3-O-β-D-apiofuranosyl-(1→2)-[6-O-(3-hydroxy-3-methylglutaryl)]-β-D-galactopyranoside (**5**)	-7.9
	Tamarixetin 3-O-β-D-apiofuranosyl-(1→2)-β-D-galactopyranoside (**4**)	-6.7
	Kaempferol-3-O-α-L-rhamnopyranosyl-(1→2)-[6-O-(3-hydroxy-3-methylglutaryl)]-β-D-galactopyranoside (**1**)	-8.1
	Mauritianin	-8.2
	Kaempferol-3-O-α-L-rhamnopyranosyl-(1→2)-β-galactopyranoside	-7.8
	Kaempferol-3-O-β-apiofuranosyl-(1→2)-β-galactopyranoside	-8.1
	Quercetin-3-O-β-apiofuranosyl-(1→2)-β-galactopyranoside	-7.6
A. gombiformis	Kaempferol-3-O-α-L-rha-(1→2)[6-O-(3-hydroxy-3-methylglutaryl)]-β-D-gal	-7.7
-	2-(2-Hxydroxy-cyclopentenyl)-pent-4-enal	-4.8
-	Chlorogenic acid	-6.6
